# Short, Tin‐Free Synthesis of All Three Inthomycins

**DOI:** 10.1002/chem.201803794

**Published:** 2018-10-19

**Authors:** Manjeet Kumar, Liam Bromhead, Zoe Anderson, Alistair Overy, Jonathan W. Burton

**Affiliations:** ^1^ Department of Chemistry University of Oxford, Chemistry Research Laboratory Mansfield Road Oxford OX1 3TA UK

**Keywords:** inthomycin, phthoxazolin, total synthesis

## Abstract

The inthomycins are a family of structurally and biologically rich natural products isolated from *Streptomyces* species. Herein the implementation of a modular synthetic route is reported that has enabled the enantioselective synthesis of all three inthomycins. Key steps include Suzuki and Sonogashira cross‐couplings and an enantioselective Kiyooka aldol reaction.

The inthomycins A–C (**1**–**3**, Figure 1), also known as the phthoxazolins, are a family of oxazole triene natural products isolated from *Streptomyces* culture that display both interesting structures and a wide range of biological activities. The isolation of inthomycin A was first reported by Ōmura in 1990^1^ who subsequently reported the isolation of inthomycins B and C in 1995.^2^ Between these dates, Zeek had reported the reisolation of inthomycin A and the first isolation of inthomycins B and C.^3^ Inthomycin A was discovered in a screen for inhibitors of cellulose biosynthesis, however, not only does inthomycin A inhibit cellulose biosynthesis,^1^, ^4^ but it also shows both herbicidal,^4^, ^5^ and antifungal^4^ activity, and both inthomycin A^6^ and inthomycin B6b inhibit prostate cancer cell growth. Very recently the cytotoxicity of inthomycin C against a range of human cancer cell lines has been investigated but the natural product showed little biological activity. However, a close analogue (**23**) was found to have proteasome inhibition activity.^7^ Apart from their biological significance, the structures of the inthomycins are particularly striking in that they contain a methylene interrupted oxazolyl‐triene moiety including a tri‐substituted alkene and a chiral allylic β‐hydroxy carbonyl moiety. Moreover, the full structural motif of the inthomycins is found within a number of more complex natural products including the oxazolomycins, 16‐methyloxazolomycin, curromycin A and B, and KSM 2690.

**Figure 1:**
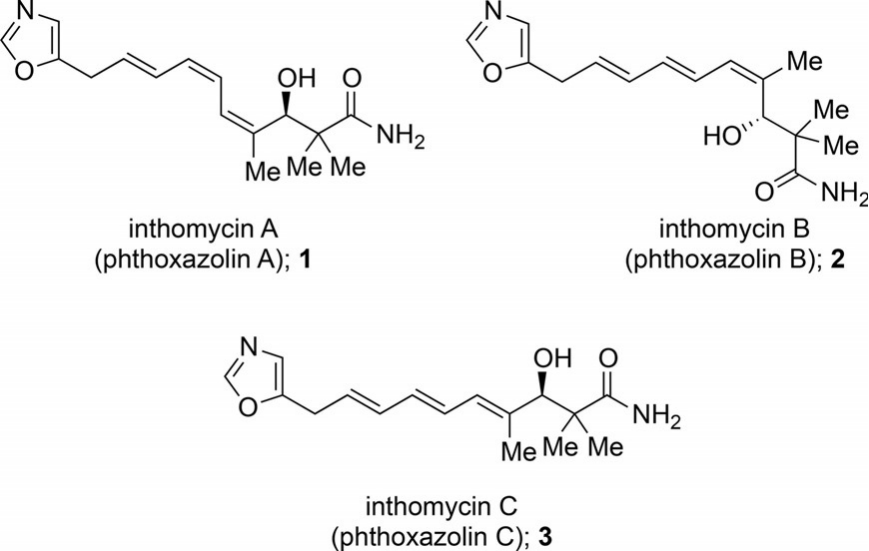
Inthomycin natural products.

Given their wide ranging biological activities and interesting structures, the inthomycins have attracted considerable attention from the synthetic community, although their deceptively simple structures belie the challenge associated with their synthesis. To date, synthetic effort has primarily focused on inthomycin C with only one report on the enantioselective synthesis of inthomycin A and only two on the enantioselective synthesis of inthomycin B. The first synthesis of inthomycin A (**1**) in racemic form was disclosed by Whiting in 1999^8^ with the only enantioselective synthesis of **1** being reported by Hatakeyama in 2012^9^ who disclosed the enantioselective synthesis of inthomycins B **2** and C **3** in the same publication. In 2006 Taylor reported the first enantioselective synthesis of inthomycin B^10^ **2** followed by a report of the enantioselective synthesis of inthomycin C **3** and of racemic inthomycin A **1** in 2008.^11^ In 2010, Ryu reported an enantioselective synthesis of inthomycin C **3** 12 which was followed by Hale's reports on the enantioselective synthesis of **3**.^13^ Very recently the Donohoe group published an enantioselective synthesis of inthomycin C **3**.^7^ All of the syntheses of the inthomycins bar one,^7^ feature a Stille cross‐coupling as a key step with the inherent problems associated with the toxicity and disposal of stoichiometric organotin waste. Herein we report short (9/10 steps, longest linear sequence), tin‐free syntheses of all three inthomycins using Suzuki or Sonogashira couplings as key steps and a Kiyooka aldol to set the necessary asymmetry.

We envisaged that inthomycins A–C (**1**–**3**), could all be prepared through cross‐coupling of the (*E*)‐ or (*Z*)‐alkenyl iodides **5** with the (*E*,*E*)‐ or (*E*,*Z*)‐dienylboronic esters **4** (Figure 2). The dienylboronic esters **4** were to be prepared by *syn* or *anti* hydroboration of the enyne oxazole **6** with the enyne oxazole **6** being prepared from alkylation of oxazole **8** with an electrophile derived from commercially available (*E*)‐pent‐2‐en‐4‐yn‐1‐ol (**7**). The iodides **5** were to be prepared using an enantioselective Kiyooka aldol reaction^14^ between the silylketene acetal **9** and the known (*E*)‐ or (*Z*)‐iodoaldehydes **10**, which can be readily prepared from propargyl alcohol **11**. This modular route allowed the ready synthesis of all three inthomycins A–C **1**–**3**.

**Figure 2:**
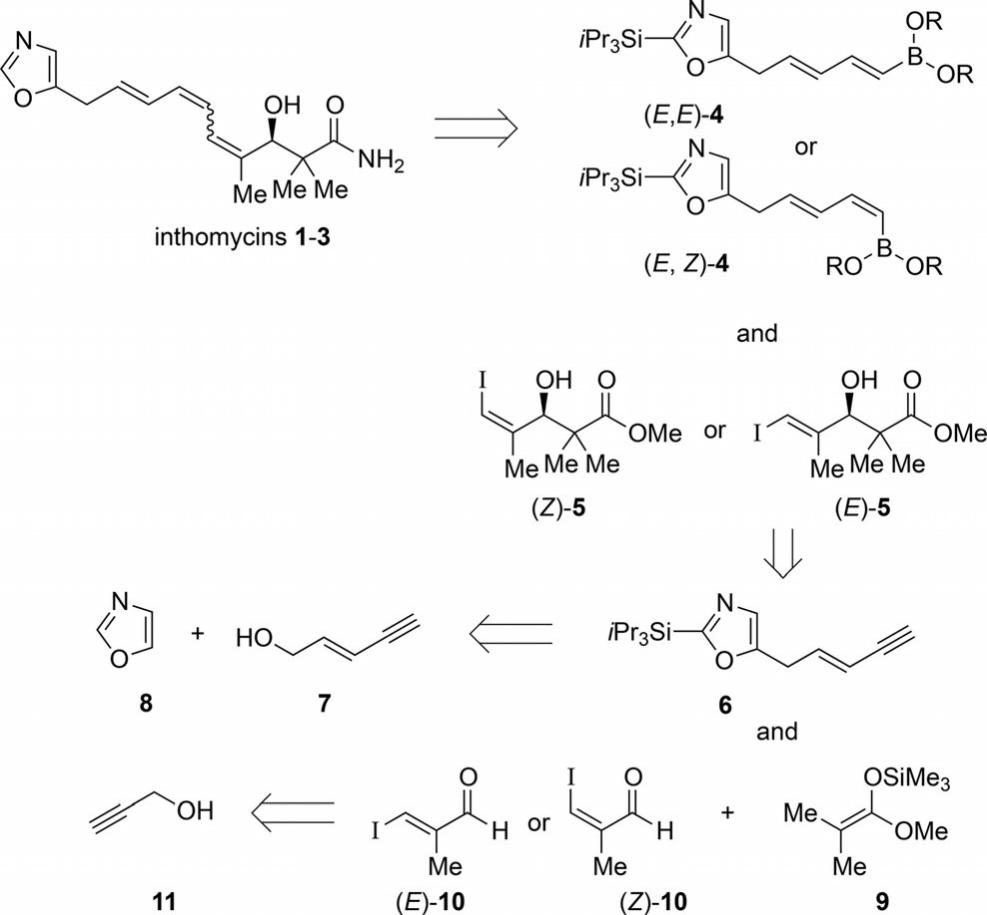
Retrosynthesis of the inthomycins **1**.

Our synthesis commenced with the preparation of the enyne oxazole **6** (Scheme 1). Commercially available (*E*)‐pent‐2‐en‐4‐yn‐1‐ol **7** 15 was readily converted into the known bromide **12** 16 in two simple steps. After careful optimization we found that lithiation of the known silyl‐protected oxazole **13** 17 with *n‐*butyllithium followed by addition to CuCN⋅LiCl and addition of the bromide **12** gave the desired coupled product **14** in 81 % yield.^18^

**Scheme 1:**
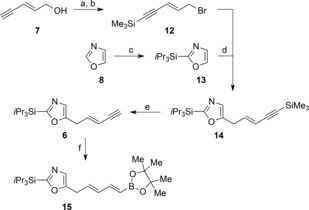
Synthesis of dienylboronic ester **15**. a) *n*BuLi, Me_3_SiCl, THF, −78 °C then 2 m HCl, 0 °C to RT, 62 %; b) PPh_3_, *N*‐bromosuccinimide, CH_2_Cl_2_, −30 °C, 81 %; c) *n*BuLi, *i*Pr_3_SiOTf, THF, −78 °C to RT, 82 %; d) **13**, *n*BuLi, CuCN⋅2LiCl, THF, −78 °C then add **12**, 81 %; e) Na_2_S, THF:H_2_O (1:1), 0 °C to RT, 85 %, after one recycle.; f) pinacol borane, (C_5_H_5_)_2_ZrHCl, Et_3_N, 60 °C, 84 %. RT=room temperature, Tf=SO_2_CF_3_, THF=tetrahydrofuran.

The next stage in the synthesis involved the seemingly simple selective silyl group removal from **14** which proved unexpectedly challenging. Commonly used basic conditions for trimethylsilyl group deprotection failed to give the desired product with allene formation being the major reaction pathway.^19^ The use of silver(I) salts to promote acetylene deprotection resulted in the formation of mixtures of starting material **14**, the desired product **6**, and fully desilylated material. Ultimately, we found that modifying Basak's procedure^20^ by using sodium sulfide in a mixture of THF and water, gave the desired mono‐desilylated product **6** without allene formation although the reaction did not reach completion; the product **6** could be obtained in 85 % yield after one recycle. Zirconium catalyzed hydroboration of the terminal acetylene in **6** gave the desired (*E*,*E*)‐dienylboronic ester **15** in good yield and with complete stereocontrol.^21^

The necessary Suzuki coupling partners for the dienylboronic ester **15** were prepared from the known (*Z*)‐ and (*E*)‐iodoalkenes (*Z*)‐**16** and (*E*)‐**16** (Scheme 2). Thus, propargyl alcohol **11** was readily converted into the (*Z*)‐ and (*E*)‐alkenyl iodides (*Z*)‐**16** 8, 22 and (*E*)‐**16** 23 using Negishi's protocols. The (*Z*)‐ and (*E*)‐alkenyl iodides (*Z*)‐**16** and (*E*)‐**16** were individually oxidized with manganese dioxide to the corresponding aldehydes **10** and subjected to the enantioselective Mukaiyama aldol reaction developed by Kiyooka^14^ using the ketene acetal **9** in the presence of l‐*N*‐tosylvaline (**18**).^24^ This gave the corresponding aldols (*Z*)‐**5** (68 % yield, 94.5:5.5 er) and (*E*)‐**5** (61 % yield, 94.5:5.5 er) which were converted into the corresponding silyl ethers (*Z*)‐**17** and (*E*)‐**17** under standard conditions.

**Scheme 2:**
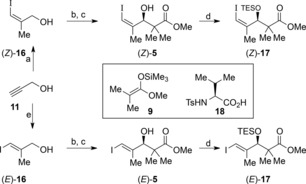
Synthesis of the alkenyl iodides (*Z*)‐ and (*E*)‐**17**. a) MeMgBr, Cu^I^I, THF, −10 °C then I_2_, −10 to −5 °C, 66 %; b) MnO_2_, CH_2_Cl_2_; c) BH_3_⋅THF, **18**, **9**, CH_2_Cl_2_, −78 °C then HCl, (*Z*)‐**5**, 68 % (2 steps), 89 % *ee*, (*E*)‐**5**, 61 % (2 steps), 89 % *ee*; d) Et_3_SiCl, imidazole, CH_2_Cl_2_, 0 °C to RT, (*Z*)‐**17** 93 %, (*E*)‐**17** 91 %; e) Me_3_Al, (C_5_H_5_)_2_ZrCl_2_, CH_2_Cl_2_, 0 °C to RT then I_2_, −78 ° to RT, 61 %.

Having established reliable routes to both the dienylboronic ester **15** and the two alkenyl iodides (*Z*)‐**17** and (*E*)‐**17**, we next addressed the key Suzuki coupling reaction (Scheme 3).^25^ After extensive experimentation we found that the use of palladium(II) acetate and triphenylphosphine in the presence of 1 m aqueous sodium bicarbonate allowed the union of the dienylboronic ester **15** with the (*Z*)‐alkenyl iodide (*Z*)‐**17** to proceed with complete stereochemical fidelity to give the corresponding coupled product **19** in 64 % yield. Double deprotection of the triene **19** with HF in acetonitrile^9^ gave the alcohol **20** which was converted into inthomycin B **2** via aminolysis of the corresponding pentaflurorophenyl ester **21**. Our synthetic inthomycin B **2** had spectroscopic properties in accord with that of both natural and synthetic inthomycin B **2**.

**Scheme 3:**
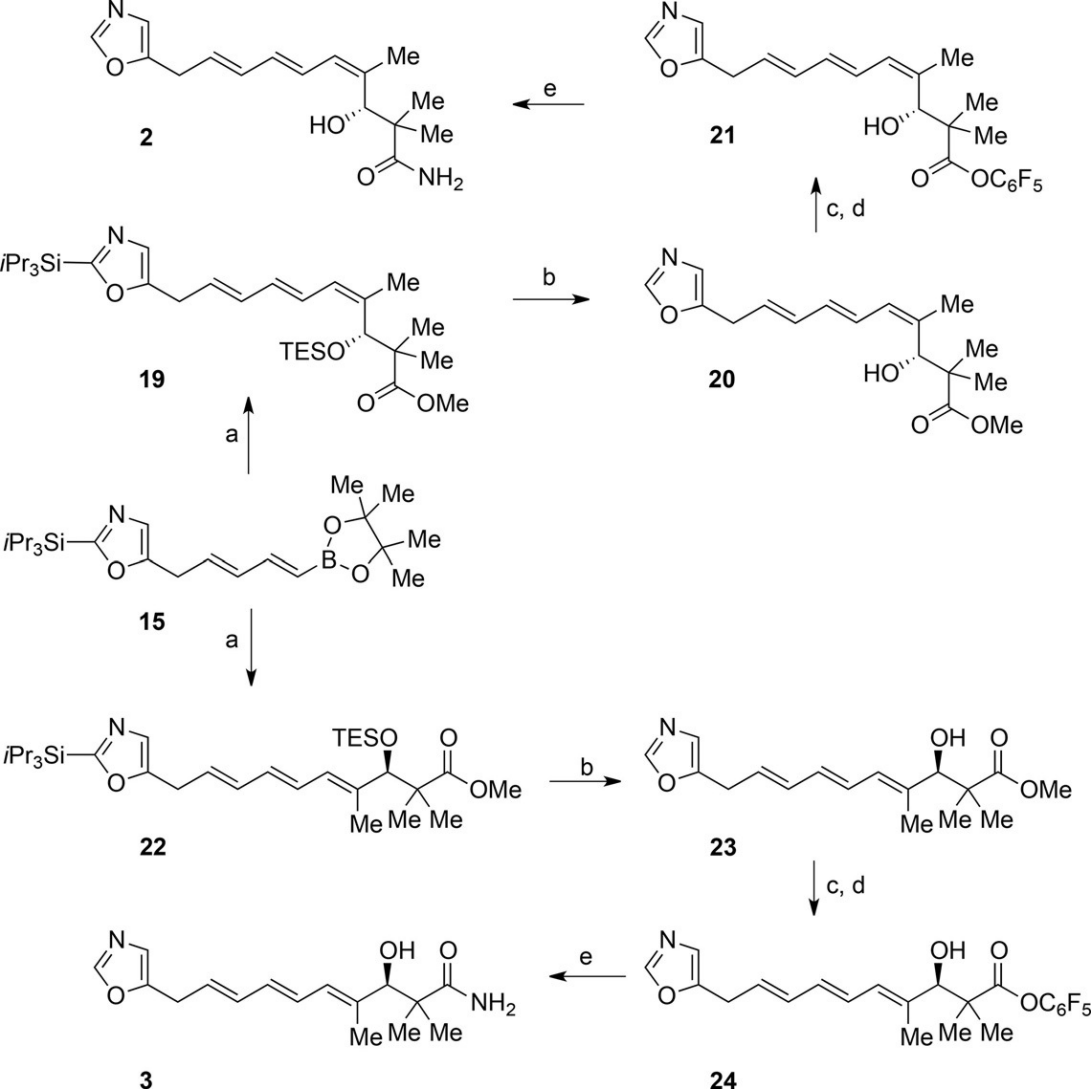
Synthesis of inthomycins B and C. a) (*Z*)‐**17** or (*E*)‐**17**, Pd(OAc)_2_, PPh_3_, Na_2_CO_3_, THF, H_2_O, **19** 64 %, **22** 65 %; b) HF⋅pyridine, CH_3_CN, 0 °C to RT, **20** 80 %, **23** 97 %; c) LiOH, H_2_O, THF, MeOH, 0 °C to RT; d) C_6_H_5_OH, EDCI⋅HCl, DMAP, CH_2_Cl_2_, **21** 80 % (2 steps), **24** 87 % (2 steps); e) NH_4_OH, THF, 0 °C to RT, **2** 95 %, **3** 94 %. EDCI=1‐ethyl‐3‐(3‐dimethylaminopropyl)carbodiimide).

For the synthesis of inthomycin C **3**, the Suzuki coupling between the (*Z*,*Z*)‐dienyl boronate **15** and the (*E*)‐alkenyl iodide (*E*)‐**17** required further optimization. Ultimately, we found that the concentration of aqueous base proved crucial with the use of 0.25 m sodium bicarbonate giving the coupled product **22** in 65 % yield. In a similar manner to the synthesis of inthomycin B **2**, inthomycin C **3** was prepared from the triene **22** by the same reaction sequence. Our synthetic inthomycin C **3** had spectroscopic properties in accord with that of both natural and synthetic inthomycin C **3**.

Having successfully synthesized inthomycins B **2** and C **3** we turned our attention to inthomycin A **1** (Scheme 4). We had originally aimed to prepare inthomycin A **1** by the same strategy namely Suzuki cross‐coupling of a (*Z*,*E*)‐dienylboronic acid (*Z*,*E*)‐**15**, however, rhodium(I) catalyzed anti‐selective hydroboration^26^ of the enyne **6** gave the corresponding (*Z*,*E*)‐dienylboronic ester (*Z*,*E*)‐**15** in low yields (<40 %) under a number of conditions. We therefore altered our synthetic strategy and investigated a Sonogashira/semi‐hydrogenation sequence. Pleasingly, the Sonogashira reaction of the alkenyl iodide (*Z*)‐**17** with the enyne **6** proceeded smoothly under standard conditions to give the coupled product **25** in 62 % yield. The next challenge was the semi‐hydrogenation of the alkyne to give the (*Z*,*Z*,*E*)‐triene required for completion of the synthesis of inthomycin A. Semi‐hydrogenation of **25** under a variety of conditions [Pd, CaCO_3_, quinoline; Pd, CaCO_3_; Pd, BaSO_4_; nickel boride; Zn (Cu/Ag)] gave mixtures of the desired product, over reduced products and starting material and we were unable to isolate the desired triene in synthetically useful yields. We therefore investigated the semi‐hydrogenation of the alcohol **26** formed by double deprotection of **25**. Pleasingly the use of Zn(Cu/Ag) couple in methanol at above room temperature gave the desired (*Z*,*Z*,*E*)‐triene **27** in 80 % yield.^27^ As before, the methyl ester **27** was readily transformed into the corresponding amide target inthomycin A **1** via the pentafluorophenyl ester **28**. Careful analysis of the ^1^H and ^13^C NMR spectra of **1** indicated that it was contaminated with a small amount (<10 %) of inthomycin B **2** which appears to arise during conversion of the ester **27** into inthomycin A **1**.

**Scheme 4:**
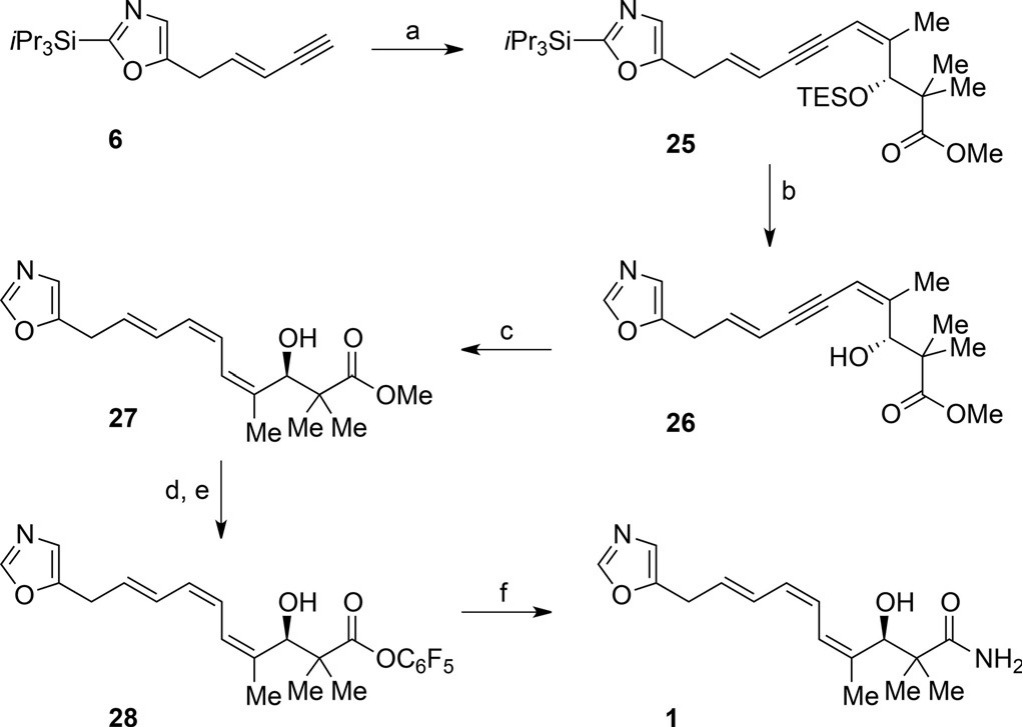
Synthesis of inthomycin A. a) (*Z*)‐**17**, Pd(PPh_3_)_4_, Cu^I^I, Et_3_N, 62 %; b) HF⋅pyridine, CH_3_CN, 0 °C to RT, 91 %; c) Zn‐Cu‐Ag couple, MeOH, 35 °C, 80 %; d) LiOH, H_2_O, THF, MeOH, 0 °C to RT; e) C_6_H_5_OH, EDCI⋅HCl, DMAP, CH_2_Cl_2_, 78 % (2 steps); f) NH_4_OH, THF, 0 °C to RT, 89 %.

All of our synthesized inthomycins had spectroscopic properties in accord with the natural and previously synthesized compounds. Importantly, the absolute configuration of inthomycin C **1** has been the subject of much confusion and debate in the literature. However, recently these ambiguities have been laid to rest by Hale and Hatakeyama13b with the absolute configuration of inthomycin C **1** being firmly established as (*R*) confirming the original assignment by Henkel and Zeek.^3^ We had assigned the absolute configuration of the alkenyl iodides (*Z*)‐**5** and (*E*)‐**5** as (*S*) using Kakisawa's extension of Mosher's method^28^ which translates into the absolute configuration of all of the inthomycins being (*R*), and our optical rotation for **3** was in agreement with the recently remeasured values.13b

In summary, we have developed efficient modular enantioselective total syntheses of all three inthomycins, which proceeds in only 9/10 steps from commercially available materials. The key steps include Suzuki and Sonogashira cross‐couplings, and an enantioselective Kiyooka aldol reaction. Our modular route has allowed the efficient syntheses of these biologically active natural products and we will use this synthetic sequence in our assault on the synthesis of the oxazolomycins.###

## Conflict of interest

The authors declare no conflict of interest.

## Supporting information

As a service to our authors and readers, this journal provides supporting information supplied by the authors. Such materials are peer reviewed and may be re‐organized for online delivery, but are not copy‐edited or typeset. Technical support issues arising from supporting information (other than missing files) should be addressed to the authors.

Supplementary
