# Supplementary material for: Short, Tin‐Free Synthesis of All Three Inthomycins
Source: Chemistry. 2018 Oct 19;24(63):16753–6. doi: 10.1002/chem.201803794 (PMC6348375; doi:10.1002/chem.201803794)
Supplement: Supplementary file 1 — Supplementary [file CHEM-24-16753-s001.pdf]

CHEMISTRY
A European Journal

Supporting Information

Short, Tin-Free Synthesis of All Three Inthomycins

Manjeet Kumar, Liam Bromhead, Zoe Anderson, Alistair Overy, and Jonathan W. Burton*[a]

chem_201803794_sm_miscellaneous_information.pdf

Experimental Procedures

General Remarks

NMR spectra ^{1}H and ^{13}C NMR spectra were recorded on a Bruker AV-500 (500/125 MHz) and Bruker AV-400 (400/100 MHz) spectrometers. Proton (δ_H) and carbon (δ_C) chemical shifts are quoted in ppm and internally referenced to the residual protonated solvent signal. Assignments were made on the basis of chemical shifts, coupling constants, COSY, DEPT, HSQC data and comparison with spectra of related compounds. Resonances are described as s (singlet), d (doublet), t (triplet), q (quartet), sept (septet), m (multiplet), dd (double doublet) and so on. Coupling constants (*J*) are given in Hz and are rounded to the nearest 0.1 Hz. H and H' refer to diastereotopic protons attached to the same carbon and imply no particular stereochemistry.

Mass Spectra High resolution mass spectra were recorded by the mass spectrometry service at the University of Oxford Chemical Research Laboratory. *m/z* values are reported in Daltons. High-resolution values are calculated to four decimal places from the molecular formula.

Infrared spectra were recorded using a Bruker Tensor 27 Fourier Transform spectrophotometer using thin films on NaCl plates using diamond ATR.

Optical rotations were measured using a UniPol L2000 polarimeter in a cell of 1 dm path length.

Melting points were determined using a Leica Galen III Compound Microscope and are uncorrected.

Analytical TLC was performed on Merck DC-Alufolien 60 F254 0.2 mm precoated plates retention factors (R_f) are reported with the solvent system used in parentheses.

Flash column chromatography was performed on Merck 60 silica (particle size 40–63 µm, pore diameter 60 Å) and the solvent system used is recorded in parentheses.

HPLC was carried out on an Agilent 1200 Series fitted with a Chiralcel OD column measuring 4.6 × 250 mm (packed with 10 µm beads) with flow rate, solvents and retention times recorded in parentheses.

All non-aqueous reactions were carried out in oven-dried glassware sealed with rubber septa under a positive pressure of dry nitrogen or argon from a manifold or balloon during the course of the reaction. Reactions were stirred using Teflon-coated magnetic stir bars. Elevated temperature reactions were maintained using a Thermowatch-controlled DrySyn heating block. Reagents were purchased from commercial suppliers and used without further purification, unless otherwise stated. Dry solvents were purified using standard techniques. Water used experimentally was deionised. Organic solutions were concentrated using a rotary evaporator. 'petrol' refers to the fraction of light petroleum ether boiling in the range of 40-60 °C as stated.

Compound names are as generated by ChemDraw Professional 16.0, NMR assignments based on either the proton/carbon environments or denoted in the structures.

Compounds not explicitly numbered in the main text are denoted **SI-1**, **SI-2**, etc. Novel compounds are denoted by the use of *italics*.

Synthesis and Compound Characterisation

Scheme 1. Synthesis of (E)-5-(pent-2-en-4-yn-1-yl)-2-(triisopropylsilyl)oxazole for Suzuki coupling

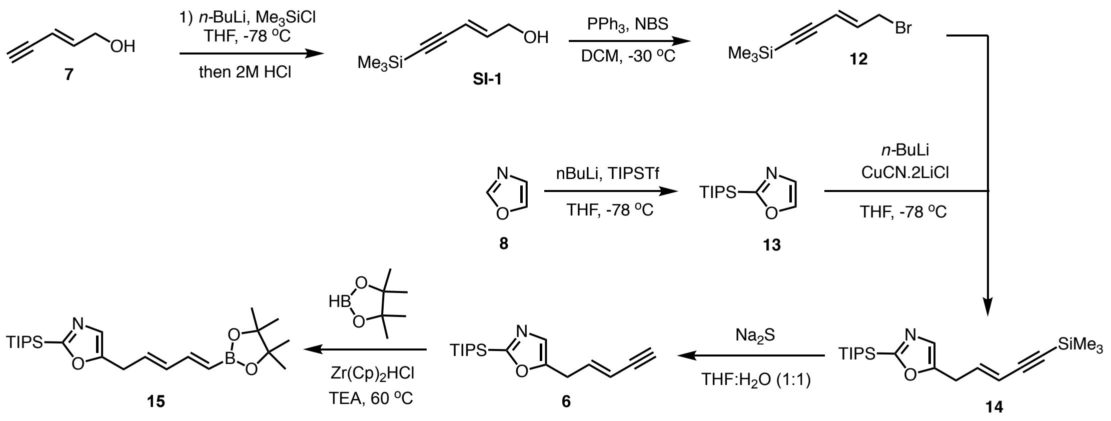

Experimental Procedures:

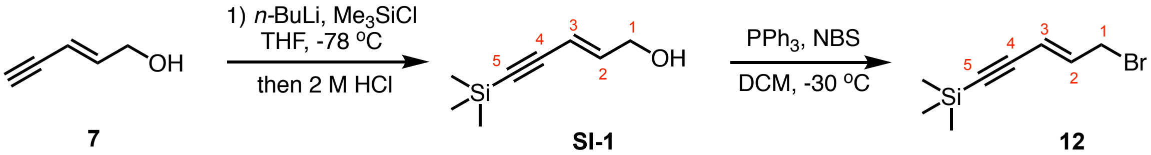

(*E*)-5-(Trimethylsilyl)pent-2-en-4-yn-1-ol (SI-1):[1] Commercial (*E*)-pent-2-en-4-yn-1-ol (**7**, 1.23 g, 15 mmol) was dissolved in dry THF (75 mL) in a 250 mL round bottom flask. The reaction flask was cooled to -78 °C, and *n*BuLi (13.2 mL, 2.5 M in hexane, 33 mmol) was added dropwise. After complete addition, the reaction mixture was warmed to -10 °C and stirred for 15 min. The reaction flask was recooled to -78 °C and trimethylsilyl chloride (4.16 mL, 3.56 g, 33 mmol) was added dropwise. After complete addition, the reaction was warmed to the room temperature over 1 h and stirring was continued for a further 2 h. The reaction flask was then cooled to 0 °C in an ice-bath and 2 M HCl (50 mL) was added. The cooling bath was removed and stirring was continued for 1 h at room temperature. The reaction mixture was diluted with Et_2O and water (50 mL). The aqueous phase was extracted with Et_2O (3 x 50 mL). The combined organic phases were washed with brine (100 mL), dried (Na_2SO_4) and concentrated *in vacuo*. Flash chromatography gave the known alcohol (**SI-1**) as pale yellow oil (1.43 g, 9.3 mmol, 62%).

R_f = 0.5 (20% EtOAc/petrol 40-60)

^{1}H NMR (400 MHz, $CDCl_3$) δ 6.30 (dt, *J* = 15.9, 5.1 Hz, 1H, **H2**), 5.76 (dt, *J* = 15.9, 1.9 Hz, 1H, **H3**), 4.19 (dd, *J* = 5.1, 1.9 Hz, 2H, **H1**), 0.18 (s, 9H, **3** x **CH_3-TMS**)

^{13}C NMR (100 MHz, $CDCl_3$) δ 142.9 (**C2**), 110.3 (**C3**), 103.0 (**C4**), 95.3 (**C5**), 62.8 (**C1**), 0.1 (**C-TMS**)

IR (neat): 3358 (br) (O-H), 2960 (C-H), 2178 (C≡C), 1682 (C=C), 1250 (CH_3-Si)

FIMS: Calcd. for $C_8H_{14}OSi$ $[M]^+$: 154.0814; found: 154.0814

Data in accord with literature values.[2]

(*E*)-(5-Bromopent-3-en-1-yn-1-yl)trimethylsilane (12):[1] (*E*)-5-(Trimethyl silyl)pent-2-en-4-yn-1-ol (**SI-1**, 0.63 g, 4.1 mmol) was dissolved in dry CH_2Cl_2 (25 mL) in a 50 mL round bottom flask. The reaction flask was cooled to -30 °C and *N*-bromosuccinimide (1.79 g, 10.1 mmol) and PPh_3 (2.65 g, 10.1 mmol) were added. The reaction mixture was stirred at this temperature for 10 min, then the cooling bath was removed and stirring was continued for 30 min. The reaction mixture was concentrated *in vacuo* and purified by flash chromatography to afford bromide (**12**) as pale yellow oil (0.71 g, 3.3 mmol, 81 %).

R_f = 0.6 (2% EtOAc/petrol 40-60)

^{1}H NMR (400 MHz, $CDCl_3$) δ 6.31 (dt, *J* = 15.6, 7.8 Hz, 1H, **H2**), 5.76 (dt, *J* = 15.6, 1.2 Hz, 1H, **H3**), 3.97 (dd, *J* = 7.8, 1.1 Hz, 2H, **H1**), 0.19 (s, 9H, **3** x **CH_3-TMS**)

^{13}C NMR (100 MHz, $CDCl_3$) δ 138.9 (**C2**), 114.3 (**C3**), 101.9 (**C4**), 97.5 (**C5**), 31.4 (**C1**), -0.3 (**C-TMS**)

IR: 2960 (C-H), 1250 (CH_3-Si), 1091, 1047

FIMS: Calcd. for $C_8H_{13}{}^{79/81}BrSi$ $[M]^+$: 215.9970 and 217.9950; found: 215.9965 and 217.9957.

Data in accord with literature values.[1]

2-(Triisopropylsilyl)oxazole (13):[3]

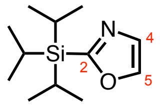

Oxazole (**8**, 1.12 g, 17 mmol) was dissolved in dry THF (50 mL) in a 100 mL round bottom flask. The reaction flask was cooled to -78 °C and *n*BuLi (7.52 mL, 2.5 M in hexane, 18.8 mmol) was added dropwise. The mixture was stirred for 10 min and warmed to -45 °C and further stirred for 15 min at this temperature. The reaction mixture was cooled to -78 °C and TIPSOTf (5.04 mL, 5.73 g, 18.8 mmol) was added dropwise. Following the complete addition, the reaction mixture was stirred at -78 °C for 2 h, and then at room temperature for 4 h. The mixture was concentrated *in vacuo* and absorbed onto silica. Purification by flash chromatography gave TIPS-oxazole (**13**) as colourless oil (3.15 g, 13.9 mmol, 82%).

R_f = 0.7 (3% EtOAc/petrol 40-60)

^{1}H NMR (400 MHz, $CDCl_3$) δ 7.78 (d, *J* = 0.7 Hz, 1H, **H5**), 7.17 (d, *J* = 0.7 Hz, 1H, **H4**), 1.48 – 1.32 (m, 3H, **3** x **CH-TIPS**), 1.10 (d, *J* = 7.5 Hz, 18H, **6** x **CH_3-TIPS**);

^{13}C NMR (100 MHz, $CDCl_3$) δ 168.5 (**C2**), 140.4 (**C5**), 126.5 (**C4**), 18.3 (**C-TIPS**), 10.9 (**C-TIPS**)

IR: 2945 (C-H), 2868, 1463, 1386, 1130 (C-O), 1069, 1019, 998, 914, 883, 757, 675, 656

HRMS ES: Calcd. for $C_{12}H_{24}ONSi$ $[M+H]^+$: 226.1622; found: 226.1622

Data in accord with literature values.[3]

(E)-2-(Triisopropylsilyl)-5-(5-(trimethylsilyl)pent-2-en-4-yn-1-yl)oxazole (14):

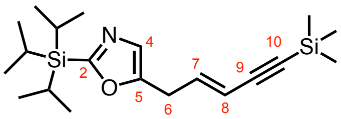

A Schlenk tube was charged with copper(I) cyanide (0.2 g, 2.3 mmol) and lithium chloride (0.2 g, 4.5 mmol), and flame dried under vacuum (three cycles of 1 min and purging with N_2 between each cycle). After cooling to room temperature, dry THF (10 mL) was added and the mixture was stirred until a clear solution was obtained. A solution of *n*BuLi (2.16 mL, 2.5 M in hexane, 5.4 mmol) was added dropwise to a solution of TIPS-oxazole (**13**, 1.01 g, 4.5 mmol) in dry THF (70 mL) at - 78 °C under N_2. After complete addition, the mixture was stirred at -30 °C for 30 min, then cooled to -78 °C. After 10 min of stirring at this temperature, the above solution of CuCN and LiCl was added dropwise via cannula. After complete addition, the reaction mixture was stirred for further 1 h at -78 °C, and bromoenyne (**12**, 1.1 g, 4.9 mmol) was added dropwise. The reaction mixture was stirred for 4 h at -78 °C and then allowed to come at room temperature and stirred overnight. The mixture was diluted with Et_2O (50 mL) and aqueous NH_4OH solution (2 M, 50 mL) was added. The mixture was stirred vigorously for 1 h to give a dark blue biphasic solution. The phases were separated and the aqueous phase was extracted with Et_2O (3 x 50 mL). The combined organic extracts were dried (Na_2SO_4) and concentrated *in vacuo*. Purification through silica chromatography gave the oxazole enyne (**14**) as pale yellow oil (1.3 g, 3.65 mmol, 81%).

R_f = 0.5 (5% EtOAc/petrol 40-60)

^{1}H NMR (400 MHz, $CDCl_3$) δ 6.86 (s, 1H, **H4**), 6.25 (dt, *J* = 15.8, 6.7 Hz, 1H, **H7**), 5.59 (dt, *J* = 15.9, 1.7 Hz, 1H, **H8**), 3.50 (ddd, *J* = 6.7, 1.6, 1.1 Hz, 2H, **H6**), 1.39 (m, 3H, **3** x **CH-TIPS**), 1.12 (d, *J* = 7.4 Hz, 18H, **6** x **CH_3-TIPS**), 0.18 (s, 9H, **3** x **CH_3-TMS**)

^{13}C NMR (100 MHz, $CDCl_3$) δ 168.1 (**C2**), 151.0 (**C5**), 139.0 (**C7**), 123.3 (**C4**), 112.4 (**C8**), 103.0 (**C9**), 94.5 (**C10**), 29.2 (**C6**), 18.4 (**C-TIPS**), 11.0 (**C-TIPS**), -0.1 (**C-TMS**)

IR: 2945 (C-H), 2868 (C-H), 2173 (C≡C), 2129, 1593, 1465 (C=C), 1250 (CH_3-Si), 1078 (C-O), 955, 883, 842, 760, 657

HRMS ES: Calcd. for $C_{20}H_{36}ONSi_2$ $[M+H]^+$: 362.2330; found: 362.2321

(E)-5-(Pent-2-en-4-yn-1-yl)-2-(triisopropylsilyl)oxazole (6):

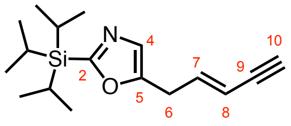

Oxazole enyne **14** (0.5 g, 1.39 mmol) was dissolved in THF:H_2O (1:1, 7 mL). The reaction mixture was cooled to 0 °C and sodium sulphide nonahydrate (0.33 g, 1.39 mmol) was added portion wise. The reaction mixture was allowed to come to room temperature and stirred for 24 h. Water was added and the aqueous phase was extracted with Et_2O (3 x 30 mL). The combined organic extracts were dried (Na_2SO_4) and concentrated *in vacuo*. Column chromatography gave the TMS-deprotected enyne (**6**, 0.26 g, 0.88 mmol, 63%) as clear colourless oil and starting material (**14**, 0.20 g, 0.55 mmol, 40 %). The starting material was subjected one more time to the same reaction conditions to give the required product in (**6**) in 85% overall yield (0.34 g, 1.18 mmol).

R_f = 0.45 (5% EtOAc/petrol 40-60)

^{1}H NMR (400 MHz, $CDCl_3$) δ 6.86 (m, 1H, **H4**), 6.30 (dtd, *J* = 16.0, 6.6, 1.0 Hz, 1H, **H7**), 5.55 (dtt, *J* = 15.9, 2.3, 1.2 Hz, 1H, **H8**), 3.52 (ddt, *J* = 6.7, 1.7, 0.9 Hz, 2H, **H6**), 2.93 – 2.81 (m, 1H, **H10**), 1.46 – 1.31 (m, 3H, **3** x **CH-TIPS**), 1.12 (d, *J* = 7.5 Hz, 18H, **6** x **CH_3-TIPS**)

^{13}C NMR (100 MHz, $CDCl_3$) δ 168.2 (**C2**), 150.9 (**C5**), 139.8 (**C7**), 123.3 (**C4**), 111.4 (**C8**), 81.5 (**C9**), 77.3 (**C10**), 29.1 (**C6**), 18.3 (**C-TIPS**), 10.9 (**C-TIPS**)

IR: 3656, 3308 (**C-H**, alkyne), 2948 (C-H), 2868, 1595, 1466 (C=C), 1386, 1252, 1156, 1075 (C-O), 958, 884, 829, 678, 657

HRMS ES: Calcd. for $C_{18}H_{28}ONSi$ $[M+H]^+$: 290.1935; found: 290.1927

5-((2E,4E)-5-(4,4,5,5-Tetramethyl-1,3,2-dioxaborolan-2-yl)penta-2,4-dien-1-yl)-2 (triisopropylsilyl)oxazole (15):

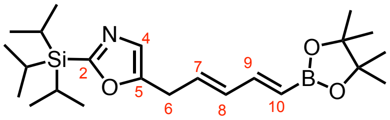

In a flame dried round bottom flask, TIPS-oxazole substrate (**6**, 200 mg, 0.69 mmol) and pinacol borane (165 µl, 145 mg, 1.3 mmol) were added at room temperature. To the stirred reaction mixture, triethylamine (21 µl, 15 mg, 0.15 mmol) and bis(cyclopentadienyl)zirconium(IV) chloride hydride (23 mg, 0.069 mmol) were added. The reaction mixture was heated to 60 °C and stirred for 4 h after which TLC indicated full consumption of starting material. The crude reaction mixture was directly purified by flash chromatography to afford the vinylboronic ester (**15**) as a colourless oil (240 mg, 0.57 mmol, 84 %).

R_f = 0.3 (5% EtOAc/petrol 40-60)

^{1}H NMR (400 MHz, $CDCl_3$) δ 7.00 (dd, *J* = 17.7, 10.4 Hz, 1H, **H9**), 6.91 – 6.78 (m, 1H, **H4**), 6.20 (ddq, *J* = 14.0, 10.4, 1.3 Hz, 1H, **H8**), 5.95 (dt, *J* = 15.2, 6.6 Hz, 1H, **H7**), 5.49 (d, *J* = 17.7 Hz, 1H, **H10**),

3.51 (d, *J* = 6.6 Hz, 2H, **H6**), 1.39 (m, 3H, **3** x **CH-TIPS**), 1.27 (s, 12H, **4** x **CH_3-OC(CH_3)$_2$**), 1.12 (d, *J* = 7.5 Hz, 18H, **6** x **CH_3-TIPS**)

^{13}C NMR (100 MHz, $CDCl_3$) δ 167.9 (**C2**), 151.9 (**C5**), 149.1 (**C9**), 134.7 (**C8**), 132.1 (**C7**), 123.0 (**C4**), 83.2 (**O<u>C</u>(CH_3)$_2$**), 28.9 (**C6**), 24.8 (**OC(<u>C</u>H$_3$)$_2$**), 18.4 (**C-TIPS**), 11.0 (**C-TIPS**). *Note*: C-10 does not appear due to quadrupolar relaxation of adjacent boron nucleus.

IR: 2925 (C-H), 2868, 1607 (C=C), 1465, 1363, 1324, 1145, 904, 725, 650

HRMS ES: Calcd. for $C_{23}H_{41}BNO_3Si$ $[M+H]^+$: 418.2943; found 418.2943

Scheme 2. Synthesis of (S,Z)-methyl-5-iodo-2,2,4-trimethyl-3-((triethylsilyl)oxy)pent-4-enoate for Suzuki Coupling:

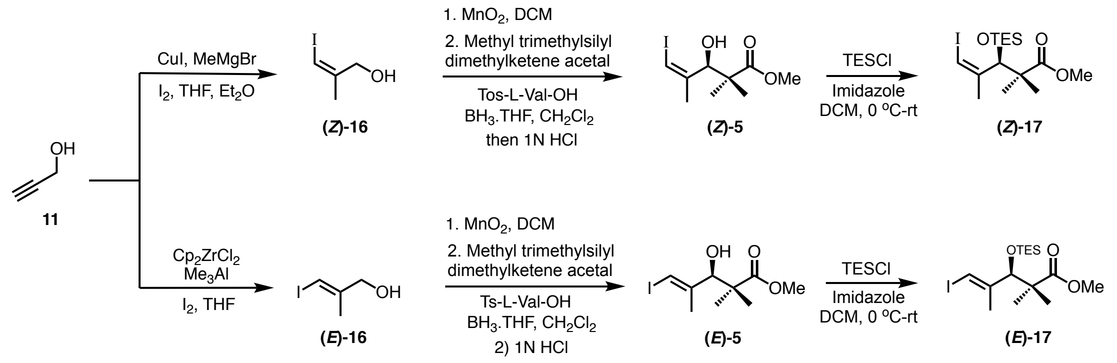

(*Z*)-3-Iodo-2-methylprop-2-en-1-ol ((*Z*)-16):[4]

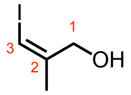

Propargyl alcohol (**11**, 3.5 mL, 60 mmol) was dissolved in THF (70 mL) in a 250 mL two neck round bottom flask. Copper(I) iodide (1.14 g, 6.0 mmol) was added and the reaction flask was cooled to -10 °C. Methylmagnesium bromide solution (3.0 M in Et_2O, 105 mmol) was added dropwise and the solution stirred for 30 min at the same temperature. Iodine (16.7 g, 66 mmol) in THF (15 mL) and Et_2O (15 mL) was added slowly via dropping funnel. The solution was stirred at -5 °C for 4 h. The solution was diluted with Et_2O (50 mL) and washed with saturated aqueous ammonium chloride (50 mL). The aqueous phase was extracted with Et_2O (3 x 50 mL). The combined organic extracts were washed with saturated aqueous $Na_2S_2O_3$ (2 x 50 mL) and brine, dried (Na_2SO_4), and the solvent removed under the vacuum to give an orange residue. The residue was purified by column chromatography to give the required product ***(Z)*-16** as an orange oil (7.8 g, 40 mmol, 66 %).

R_f = 0.6 (20% EtOAc/petrol 40-60)

^{1}H NMR (400 MHz, CDCl$_3$) δ 5.94 (s, 1H, **H3**), 4.19 (d, *J* = 0.7 Hz, 2H, **H1**), 1.94 (d, *J* = 1.5 Hz, 3H, **C2-CH$_3$**)

^{13}C NMR (100 MHz, CDCl$_3$) δ 145.9 (**C2**), 74.7 (**C3**), 67.8 (**C1**), 21.5 (**C2-<u>C</u>H$_3$**)

IR: 3300 (br, O-H), 2913 (C-H), 1621 (C=C), 1067 (C-O)

HRMS ES: Calcd. for C_4H_7IO $[M]^+$: 197.9542; found: 197.9544

Data in accord with literature values.[5]

N-Tosyl-L-valine (18):[6]

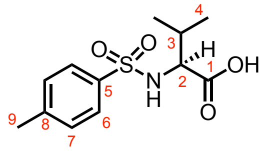

A solution of tosyl chloride (13.6 g, 71.1 mmol) in THF (140 mL) was added slowly to a solution of L-valine (10.0 g, 85.4 mmol), K_2CO_3 (11.8 g, 85.4 mmol) and KOH (4.80 g, 85.4 mmol) in water (170 mL) at room temperature. The reaction mixture was vigorously stirred for 18 h. The mixture was acidified to pH 2, and Et_2O (75 mL) was added. The phases were separated and the aqueous phase was further extracted with Et_2O (3 x 50 mL). The combined organic phases were dried over Na_2SO_4 and concentrated in vacuo. Recrystallisation (EtOH/H_2O) gave the tosylated product (**18**) as a bright white crystalline solid (14.2 g, 52.3 mmol, 61 %)

$[\alpha]_D^{25}$ = +27.5 (c=1.08 in EtOH); lit.[6] $[\alpha]_D^{24}$= +27.6 (c=1.0 in EtOH)

m.p. = 148.1 - 150.3 °C; lit.[6] m.p. = 153-154 °C

^{1}H NMR (400 MHz, CDCl$_3$) δ 10.28 (s, 1H, **OH**), 7.65 (d, *J* = 8.0 Hz, 2H, **H6**), 7.21 (d, *J* = 8.0 Hz, 2H, **H7**), 5.18 (d, *J* = 9.8 Hz, 1H, **NH**), 3.71 (dd, *J* = 10.0, 4.6 Hz, 1H, **H2)**, 2.34 (s, 3H, **H9**), 2.12 – 1.89 (m, 1H, **H3**), 0.88 (d, *J* = 6.7 Hz, 3H, **H4/4'**), 0.79 (d, *J* = 6.8 Hz, 3H, **H4/4'**)

^{13}C NMR (100 MHz, CDCl$_3$) δ 176.6 (**C1**), 143.9 (**C5**), 136.5 (**C8**), 129.7 (**2 x C6**), 127.3 (**2 x C7**), 60.6 (**C2**), 31.4 (**C3**), 21.6 (**C9**), 19.0 (**C4/4'**), 17.1 (**C4/4'**)

Data in accord with literature values.[6]

Methyl (*S*,*Z*)-3-hydroxy-5-iodo-2,2,4-trimethylpent-4-enoate ((*Z*)-5):

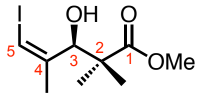

The entire reaction, including filtrations and concentrations, until the final quench was performed with exclusion of light.

MnO_2 (6.4 g, 73 mmol) was added to a vigorously stirred solution of the vinyl iodide **(*Z*)-16** (1.45 g, 7.33 mmol) in dry CH_2Cl_2 (30 mL) under N_2. The reaction mixture was stirred for 1.5 h, and then a further 10 eq. of MnO_2 (6.4 g, 73 mmol) was added. The reaction mixture was stirred for a further 1 h until TLC indicated complete consumption of starting material. The reaction was filtered through a short plug of Celite™, eluting with CH_2Cl_2 (3 x 5 mL) and the filtrate was concentrated *in vacuo* to give the crude aldehyde. This was dissolved in dry CH_2Cl_2 (5 mL) and kept in the dark under nitrogen before use.

A solution of BH_3•THF (1 M, 6.7 mL, 6.7 mmol) was added dropwise to a solution of *N*-tosyl-L-valine (1.98 g, 7.33 mmol) in dry CH_2Cl_2 (75 mL) at 0 °C under nitrogen, and the mixture was stirred for 30 minutes at this temperature. After this, the reaction mixture was cooled to -78 °C and the solution of the above aldehyde (assumed to be 7.33 mmol) in CH_2Cl_2 (5 mL) was added dropwise. After complete addition, a solution of methyl trimethylsilyl dimethylketene acetal (**9**, ex-Aldrich 1.80 mL, 8.65 mmol) was added dropwise and the reaction mixture was stirred at -78 °C for 4 h. Subsequently, 1 M aqueous HCl (100 mL) was added and the reaction mixture was warmed to room temperature and stirred for 1 h. The phases were separated and the aqueous phase was extracted with EtOAc (3 x 50 mL). The combined organic extracts were evaporated under reduced pressure and the crude was again dissolved in THF. Further 1 M HCl was added to the reaction mixture and stirred for 30 min. The phases were separated and the aqueous phase was extracted with EtOAc (3 x 50 mL). The combined organic extracts were washed with H_2O (50 mL), saturated aqueous $NaHCO_3$ solution (50 mL) and brine (50 mL), dried (Na_2SO_4) and concentrated *in vacuo*. Flash chromatography gave the free alcohol product **(*Z*)-5** as a clear colourless oil (1.48 g, 4.95 mmol, 68 %).

$[\alpha]_D^{25}$ -48.0 (*c* = 1.7 in $CHCl_3$).

HPLC: HPLC: e.e. 89% IPA:Hexane (2:98); 0.4 mL/min; OD chiral column; 21.54 (major) and 23.85 (minor).

R_f = 0.5 (10% EtOAc/petrol 40-60)

^1H NMR (400 MHz, $CDCl_3$) δ 6.17 (m, 1H, **H5**), 4.62 (s, 1H, **H3**), 3.74 (s, 3H, **OCH_3**), 1.80 (d, *J* = 1.5 Hz, 3H, **C4-CH_3**), 1.40 (s, 3H, **C2-CH_3**), 1.21 (s, 3H, **C2-CH_3**);

^{13}C NMR (100 MHz, $CDCl_3$) δ 178.4 (**C1**), 145.3 (**C4**), 80.8 (**C5**), 79.2 (**C3**), 52.3 (**O<u>C</u>H_3**), 46.0 (**C2**), 25.0 (**C2-<u>C</u>H_3**), 20.8 (**C4-<u>C</u>H_3**), 19.3 (**C2-<u>C</u>H_3**)

IR: 3485 (br, O-H), 2951 (C-H), 1721 (C=O), 1143 (C-O, ester), 1082 (C-O, alcohol).

HRMS CI: Calcd. for $C_9H_{15}O_3I$ $[M+H]^+$: 299.0139; found: 299.0138

Methyl (S,Z)-5-iodo-2,2,4-trimethyl-3-((trimethylsilyl)oxy)pent-4-enoate (Z-17):

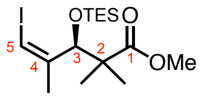

Alcohol substrate **(*Z*)-5** (467 mg, 1.57 mmol) was dissolved in dry CH_2Cl_2 (10 mL) in a 50 mL round bottom flask. The reaction flask was cooled down to 0 °C and imidazole (159 mg, 2.35 mmol) was added and the mixture was stirred for 5 min. Triethylsilyl chloride (392 µl, 2.35 mmol) was added dropwise and stirring was continued for 2 h at the same temperature. The solvent was evaporated *in vacuo* and purification by flash column chromatography gave the required product (***Z*-17**) as pale yellow oil (0.60 g, 1.46 mmol, 93 %).

$[\alpha]_D^{25}$ = +46.8 (c = 0.77 in $CHCl_3$)

R_f = 0.8 (5% EtOAc/petrol 40-60)

^{1}H NMR (400 MHz, $CDCl_3$) δ 6.13 (q, *J* = 1.5 Hz, 1H, **H5**), 4.90 (s, 1H, **H3**), 3.68 (s, 3H, **OCH_3**), 1.89 (d, *J* = 1.4 Hz, 3H, **C4-CH_3**), 1.24 (s, 3H, **C2-CH_3**), 1.22 (s, 3H, **C2-CH_3**), 0.94 (t, *J* = 7.9 Hz, 9H, **3** x **CH_3-TES**), 0.62 – 0.55 (m, 6H, **3** x **CH_2-TES**)

^{13}C NMR (100 MHz, $CDCl_3$) δ 176.7 (**C1**), 146.7 (**C4**), 79.8 (**C5**), 78.3 (**C3**), 51.8 (**O<u>C</u>H_3**), 49.3 (**C2**), 22.8 (**C2-<u>C</u>H_3**), 20.9 (**C4-<u>C</u>H_3**), 20.2 (**C2-<u>C</u>H_3**), 6.8 (**C-TES**), 4.7 (**C-TES**)

IR: 2953 (C-H), 2877, 1734 (C=O), 1460 (w), 1247, 1144, 1127 (C-O, ester), 1085 (C-O, alcohol), 1015, 847, 742

HRMS ES: Calcd. for $C_{15}H_{30}O_3ISi$ $[M+H]^+$: 413.1003; found: 413.1004

(*E*)-3-Iodo-2-methylprop-2-en-1-ol ((*E*)-16):[7]

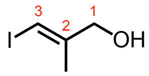

Bis(cyclopentadienyl)zirconium(IV) dichloride (1.02 g, 3.5 mmol) was dissolved in CH_2Cl_2 (40 mL) and cooled to 0 °C. A solution of trimethylaluminium (21 mL, 2 M in toluene, 42 mmol) was added dropwise and the solution stirred for 10 min. at 0 °C. Propargyl alcohol (**11**, 0.79 g, 14 mmol) was dissolved in CH_2Cl_2 (5 mL) and added to the above solution *via* cannula, dropwise at 0 °C. The solution was stirred overnight at room temperature. The solution was cooled to -78 °C and iodine (4.2 g, 16.8 mmol) in THF (5 mL) was added *via* cannula, washing with additional THF (1 mL). The solution was allowed to warm to room temperature and was stirred for 1 h. The reaction was quenched with 2 M HCl solution and extracted with EtOAc (3 x 30 mL). The combined organic phases were washed with brine (30 mL), dried (Na_2SO_4) and concentrated *in vacuo* at 5-10 °C water bath temperature. Flash chromatography gave the required product **(*E*)-16** as pale yellow oil (1.68 g, 8.5 mmol, 61 %).

R_f = 0.4 (20% EtOAc/petrol 40-60)

^{1}H NMR (400 MHz, $CDCl_3$): δ 6.28 (s, 1H, **H3**), 4.13 (d, *J* = 4.5, 2H, **H1**), 1.85 (d, *J* = 1.2, 3H, **C2-CH$_3$**)

^{13}C NMR (100 MHz, $CDCl_3$): δ 147.2 (**C2**), 77.3 (**C3**), 67.2 (**C1**), 21.3 (**C2-<u>C</u>H$_3$**)

IR: 3300 (br, O-H), 2913 (C-H), 1621 (C=C), 1442, 1275, 1067 (C-O)

FIMS: Calcd. for C_4H_7IO $[M]^+$: 197.9542; found: 197.9544

Data in accord with literature values.[8]

Methyl (S,E)-3-hydroxy-5-iodo-2,2,4-trimethylpent-4-enoate ((E)-5):

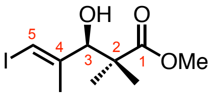

In the same manner as described for the synthesis compound **(*Z*)-5**, substrate (***E*)-16** (0.79 g, 14 mmol) gave the required product (***E*)-5** (1.68 g, 8.53 mmol) as colorless oil in 61% yield after purification by column chromatography.

$[\alpha]_D^{25}$ + 42.7 (c = 0.29 in $CHCl_3$)

HPLC: *e.e.* 89%, IPA:Hexane (2:98); 1 mL/min; OD chiral column; 12.50 min (minor) and 16.92 min (major).

R_f = 0.5 (10% EtOAc/petrol 40-60)

^{1}H NMR (400 MHz, $CDCl_3$): δ 6.25 (s, 1H, **H5**), 4.27 (dd, *J* = 5.9, 0.9 Hz, 1H, **H3**), 3.68 (s, 3H, **OCH$_3$**), 3.41 (d, *J* = 5.9 Hz, 1H, **OH**), 1.75 (d, *J* = 1.1 Hz, 3H, **C4-CH$_3$**), 1.18 (s, 3H, **C2-CH$_3$**), 1.12 (s, 3H **C2-CH$_3$**)

^{13}C NMR (100 MHz, $CDCl_3$): δ 177.7 (**C1**), 146.8 (**C4**), 81.4 (**C5**), 81.4 (**C3**), 52.2 (**OCH$_3$**), 46.3 (**C2**), 23.4 (**C2-CH$_3$**), 21.3 (**C4-CH$_3$**), 20.6 (**C2-CH$_3$**)

IR: 3484 (br, O-H), 2949 (C-H), 1720 (C=O), 1469, 1270, 1134 (C-O, ester), 1059 (C-O, alcohol)

HRMS ES: Calcd. for $C_9H_{15}O_3INa$ $[M+Na]^+$: 320.9958; found $[M+Na]^+$ = 320.9961

Methyl (*S*,*E*)-5-iodo-2,2,4-trimethyl-3-((trimethylsilyl)oxy)pent-4-enoate (*E*-17):

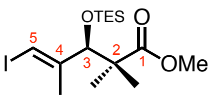

Alcohol substrate **(*E*)-5** (103 mg, 0.34 mmol) was dissolved in dry CH_2Cl_2 (5 mL) in a 25 mL round bottom flask. The reaction flask was cooled to 0 °C and imidazole (37 mg, 0.55 mmol) was added. Stirring was continued for 5 min and then triethylsilyl chloride (83 µl, 74 mg, 0.50 mmol) was added dropwise and stirring was continued for 2 h. The solvent was evaporated *in vacuo* and

purification through a short pad of silica gave the required product **(*E*)-17** as pale yellow oil (0.127 g, 0.31 mmol, 91 %).

$[\alpha]_D^{25}$ - 76.4 (c= 0.74 in $CHCl_3$)

R_f = 0.8 (5% EtOAc/petrol 40-60)

^{1}H NMR (400 MHz, $CDCl_3$): δ 6.22 (s, 1H, **H5**), 4.48 (s, 1H, **H3**), 3.64 (s, 3H, **OCH_3**), 1.76 (d, *J* = 1.1 Hz, 3H, **C4-CH_3**), 1.16 (s, 3H, **C2-CH_3**), 1.04 (s, 3H, **C2-CH_3**), 0.90 (t, *J* = 7.9 Hz, 9H, **3** x **CH_3-TES**), 0.58 – 0.47 (m, 6H, **3** x **CH_2-TES**)

^{13}C NMR (100 MHz, $CDCl_3$): δ 176.7 (**C1**), 147.9 (**C4**), 81.3 (**C5**), 81.0 (**C3**), 51.8 (**C8**), 48.6 (**C2**), 22.0 (**C7**), 21.4 (**C6/6'**), 20.9 (**C6/6'**), 6.7 (**CH_3-TES**), 4.5 (**CH_2-TES**)

IR: 2952 (C-H), 1729 (C=O), 1466, 1270 (Si-C), 1089 (C-O)

HRMS ES: Calcd. for $C_{15}H_{30}O_3ISi$ $[M+H]^+$: 413.1003; found: 413.1003.

Scheme 3. Synthesis of Inthomycin B and C

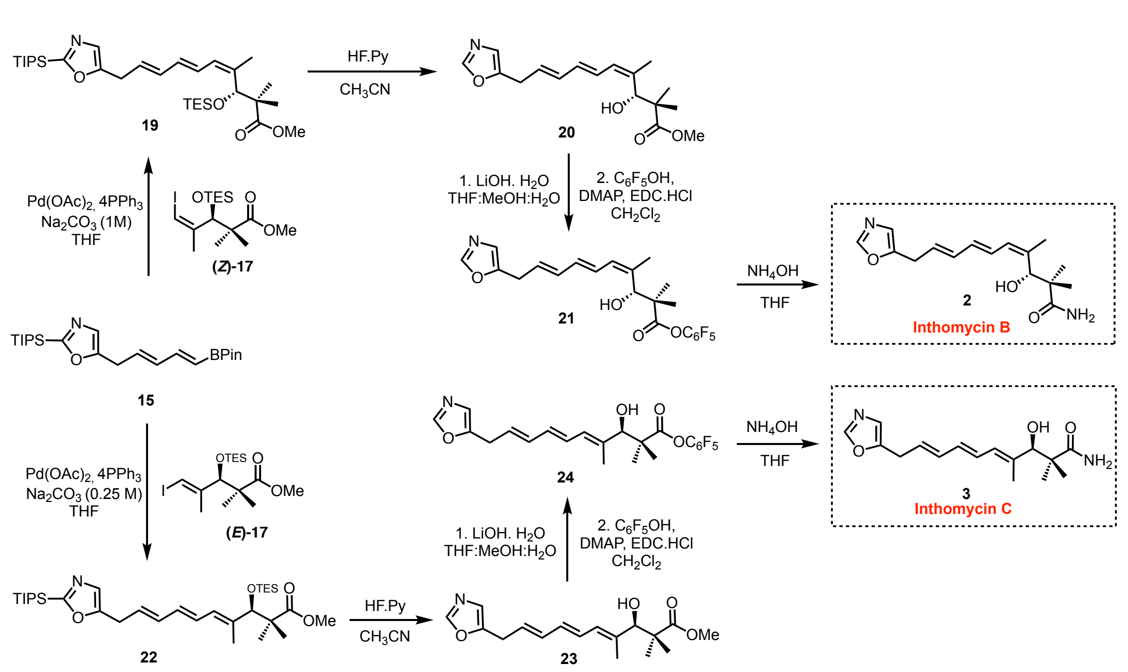

Methyl (R,4Z,6E,8E)-2,2,4-trimethyl-3-((triethylsilyl)oxy)-10-(2-(triisopropylsilyl)oxazol-5-yl) deca-4,6,8-trienoate (19):

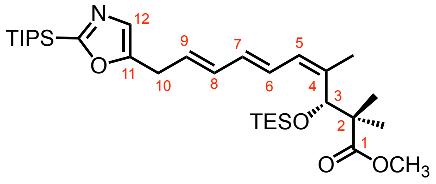

In 25 mL round bottom flask, boronic ester (**15**, 137 mg, 0.32 mmol) and alkenyl iodide (**(*Z*)-17**, 162 mg, 0.39 mmol) were dissolved in THF (3 mL) and purged with argon for 15 mins. Palladium(II) acetate (7 mg, 0.032 mmol) and triphenylphosphine (33 mg, 0.128 mmol) were then added followed by degassed 1 M Na_2CO_3 (3.2 mL, 3.2 mmol). The reaction mixture was stirred at room temperature for 10 h, warmed to 40 °C and stirred for a further 1 h to allow complete consumption of the boronic ester substrate. The reaction mixture was diluted with Et_2O and sat. brine solution. The aqueous phase was extracted with Et_2O (3 x 25 mL) and the solvent was removed *in vacuo*. The crude product was absorbed onto silica and purification by flash chromatography gave the coupled product (**19**) as a colourless oil (118 mg, 0.21 mmol, 64 %).

$[\alpha]_D^{25}$ +57.1 (c = 0.84 in $CHCl_3$)

R_f = 0.4 (5% EtOAc/petrol 40-60)

A number of broad resonances were observed in both the ^{1}H and ^{13}C NMR spectra at 20 °C. Acquisition of the spectra in d_6-benzene at 75 °C resulted in sharpening of some resonances. NMR data at both temperatures are given below:

^{1}H NMR (400 MHz, 20 °C, $CDCl_3$) δ 6.85 (s, 1H, **H12**), 6.50 – 6.30 (m, 1H, **H6**), 6.26 – 6.05 (m, 2H, **H8,7**), 5.98 (d, *J* = 11.3 Hz, 1H, **H5**), 5.76 (dt, *J* = 14.3, 6.6 Hz, 1H, **H9**), 4.94 (s, 1H, **H3**), 3.62 (s, 3H, **OCH_3**), 3.51 (d, *J* = 6.6 Hz, 2H, **H10**), 1.79 (d, *J* = 1.4 Hz, 1H, **C4-CH_3**), 1.47 -1.35 (m, 3H, **3** x **CH-TIPS**), 1.21, (s, 3H, **C2-CH_3**), 1.13 (d, *J* = 7.4 Hz, 18H, **6** x **CH_3-TIPS**), 1.08 (s, 3H, **C2-CH_3**), 0.92 (t, *J* = 7.9 Hz, 9H, **3** x **CH_3-TES**), 0.54 (q, *J* = 8.2 Hz, 6H, **3** x **CH_2-TES**)

^{13}C NMR (100 MHz, 20 °C, $CDCl_3$) δ 177.1 (**C1**), 167.8 (**C13**), 152.4 (**C11**), 138.2 (**C4**), 133.1 (**C8**), 131.6 (**C7**), 129.4 (**C5**), 128.0 (**C6**) 127.6 (**C9**), 122.9 (**C12**), 74.3 (**C3**), 51.7 (**O<u>C</u>H_3**), 49.5 (**C2**), 29.0 (**C10**), 22.2 (**C2-<u>C</u>H_3**), 21.3 (**C2-<u>C</u>H_3**), 18.4 (**C4-CH_3**), 18.4 (**C-TIPS**), 11.0 (**C-TIPS**), 6.8 (**C-TES**), 4.7 (**C-TES**).

^{1}H NMR (500 MHz, 75 °C, C_6D_6) δ 6.78 (s, 1H, **H12**), 6.63(t, *J* = 12.6 Hz, 1H, **H6**), 6.14 – 5.99 (m, 2H, **H8, 7**), 5.93 (d, *J* = 11.4 Hz, 1H, **H5**), 5.56 (dt, *J* = 14.2, 6.6 Hz, 1H, **H9**), 5.19 (s, 1H, **H3**), 3.43 (s, 3H, **OCH_3**), 3.18 (d, *J* = 6.6 Hz, 2H, **H10**), 1.83 (s, 3H, **C4-CH_3**), 1.54-1.34 (m, 3H, **3** x **CH-TIPS**), 1.36, (s, 3H, **C2-CH_3**), 1.21 (d, *J* = 7.4 Hz, 18H, **6** x **CH_3-TIPS**), 1.20 (s, 3H, **C2-CH_3**), 1.00 (t, *J* = 7.9 Hz, 9H, **3** x **CH_3-TES**), 0.64 (q, *J* = 8.2 Hz, 6H, **3** x **CH_2-TES**);

^{13}C NMR (125 MHz, 75 °C, C_6D_6) δ 176.1(**C1**), 167.3 (**C13**), 152.2 (**C11**), 137.8 (**C4**), 133.0 (**C8**), 132.2 (**C7**), 132.0 (**C5**), 129.7 (**C6**), **127.4 (C9**), 123.2 (**C12**), 75.3 (**C3**), 50.9 (**O<u>C</u>H_3**), 49.4 (**C2**), 28.7 (**C10**), 22.2 (**C2-<u>C</u>H_3**), 21.6 (**C2-<u>C</u>H_3**), 21.2 (**C4-<u>C</u>H_3**), 18.2 (**C-TIPS**), 11.4 (**C-TIPS**), 6.5 (**C-TES**), 5.1 (**C-TES**).

IR: 2949 (C-H, s), 2870 (C-H, s), 1740 (C=O), 1593 (C=C), 1465, 1386, 1259, 1122 (C-O, ester), 1079, 987 (C=C, bend), 884, 846, 741, 677, 658

HRMS ES: Calcd. for $C_{32}H_{58}NO_4Si_2$ $[M+H]^+$ = 576.3899, found $[M+H]^+$ = 576.3899

Methyl (*R*,4*Z*,6*E*,8*E*)-3-hydroxy-2,2,4-trimethyl-10-(oxazol-5-yl)deca-4,6,8-trienoate (20):

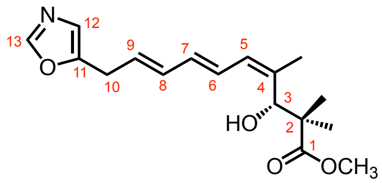

In a 20 mL plastic vial, substrate (**19**, 118 mg, 0.21 mmol) was dissolved in MeCN (3 mL) and cooled to 0 °C. HF-pyridine solution (30%, 100 µl, 0.25 mmol) was added dropwise and the solution was stirred at room temperature until TLC analysis indicated the complete consumption of starting material (24 h). The mixture was cooled to 0 °C, diluted with water and neutralized with sat. $NaHCO_3$ solution. The aqueous phase was extracted with EtOAc (3 x 20 mL), the combined organic phases were washed with brine (30 mL), dried (Na_2SO_4) and concentrated *in vacuo*. Flash chromatography afforded the required product **(20)** as pale yellow oil (51 mg, 0.17 mmol, 80 %).

$[\alpha]_{\mathrm{D}}^{25}$ +44.0 (c = 0.57 in $CHCl_3$); (lit.[9] $[\alpha]_{\mathrm{D}}^{27}$ +81.9 (c 0.99, $CHCl_3$), lit.[10]for material of 64% ee $[\alpha]_{\mathrm{D}}^{21}$ +57.9 (c 10.80, $CHCl_3$)). The optical rotation of our sample of **20** was different from those reported in the literature, however, the optical rotation of inthomycin B derived from **20** is in keeping with the literature values (*vide infra*). We measured the enantiomeric excess of a derivative prepared from **20** by chiral HPLC to confirm that the C-3 stereocentre had not been compromised during the silyl group deprotection.

R_f = 0.2 (30% EtOAc/petrol 40-60)

^{1}H NMR (400 MHz, $CDCl_3$): δ 7.78 (s, 1H, **H13**), 6.78 (d, *J* = 1.2 Hz, 1H, **H12**), 6.42 (dd, *J* = 14.0, 11.3 Hz, 1H, **H6**), 6.28 – 6.06 (m, 2H, **H8, 7**), 6.02 (dd, *J* = 11.5, 1.6 Hz, 1H, **H5**), 5.72 (dt, *J* = 14.2, 6.8 Hz, 1H, **H9**), 4.71 (d, *J* = 6.4 Hz, 1H, **H3**), 3.70 (s, 3H, **OCH₃**), 3.47 (d, *J* = 7.0 Hz, 2H, **H10**), 3.40 (d, *J* = 6.5 Hz, 1H, **OH**), 1.74 (s, 3H, **C4-CH₃**), 1.26 (s, 3H, **C2-CH₃**), 1.15 (s, 3H, **C2-CH₃**).

^{13}C NMR (100 MHz, $CDCl_3$): δ 178.3 (**C1**), 150.7 (**C11**), 150.4 (**C13**), 136.8 (**C4**), 133.3 (**C8**), 131.7 (**C7**), 130.1 (**C5**), 127.8 (**C6**), 127.2 (**C9**), 122.5 (**C12**), 75.0 (**C3**), 52.2 (**O<u>C</u>H₃**), 46.8 (**C2**), 28.8 (**C10**), 24.3 (**C2-<u>C</u>H₃**), 21.0 (**C2-<u>C</u>H₃**), 19.5 (**C4-<u>C</u>H₃**).

IR: 3392(br, O-H), 2950 (C-H), 1726 (C=O), 1510 (C=C), 1470, 1434, 1389, 1258, 1191, 1141, 1120 (C-O, ester), 1055 (C-O), 988 (C=C, bend), 828, 647

HRMS ES: Calcd. for $C_{17}H_{23}NO_4Na$ $[M+Na]^+$ = 328.1519, found $[M+Na]^+$ = 328.1519

Perfluorophenyl (*R*,4*Z*,6*E*,8*E*)-3-hydroxy-2,2,4-trimethyl-10-(oxazol-5-yl)deca-4,6,8-trienoate (21):

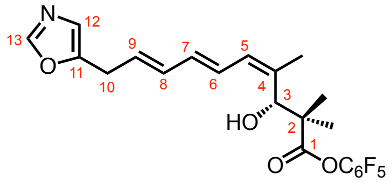

To the stirred solution of methyl ester (**20**, 15 mg, 0.05 mmol) in THF (1 mL) was added MeOH:H_2O (1:1, 0.6 mL). Light was excluded from the reaction flask and the reaction mixture was cooled 0 °C and LiOH•H_2O (5 mg, 0.12 mmol) was added. The orange coloured reaction mixture was allowed to come to room temperature and stirred overnight until TLC analysis indicated the complete consumption of starting material. The reaction mixture was recooled to 0 °C, diluted with EtOAc and acidified with 1 M aqueous HCl to pH 3-4. The aqueous phase was extracted with Et_2O (3 x 20 mL). The combined organic phases were washed with brine (10 mL), dried (Na_2SO_4) and concentrated *in vacuo*. The crude acid was dissolved in dry CH_2Cl_2 (2 mL) at 0 °C and added a solution of 1,2,3,4,5-pentafluorophenol (12 mg, 0.06 mmol) in CH_2Cl_2 (1 mL) and N-(3-dimethylaminopropyl)-N-ethylcarbodiimide hydrochloride (10 mg, 0.05 mmol) was added. After the addition, 4-(dimethylamino)pyridine (2 mg, 0.02 mmol) was added and the reaction mixture was stirred for 2 h. The solvent was evaporated under a N_2 flow and purification of the residue by flash column chromatography gave the required product (**21**) as a colourless oil (18 mg, 0.04 mmol, 80 %).

$[\alpha]_{\mathrm{D}}^{25}$ + 82.8 (c= 0.22 in $CHCl_3$)

R$_f$ = 0.5 (20% EtOAc/petrol 40-60)

^{1}H NMR (400 MHz, $CDCl_3$): δ 7.82 (s, 1H, **H13**), 6.82 (s, 1H, **H12**), 6.47 (dd, *J* = 11.4, 13.8, 1H, **H6**), 6.25 – 6.17 (m, 2H, **H8, 7**), 6.12 (dd, *J* = 11.8, 1.7 Hz, 1H, **H5**), 5.77 (dt, *J*= 14.3, 6.8, 1H, **H9**), 5.04 (d, *J* = 5.3 Hz, 1H, **H3**), 3.50 (d, *J* = 6.8, 2H, **H10**), 2.43 (d, *J* = 5.4 Hz, 1H, **OH**), 1.89 (s, 3H, **C4-CH$_3$**), 1.40 (s, 3H, **C2-CH$_3$**), 1.37 (s, 3H, **C2-CH$_3$**).

^{13}C NMR (100 MHz, $CDCl_3$): δ 173.7 (**C1**), 150.8 (**C11**), 150.5 (**C13**), 135.7 (**C4**), 133.3 (**C8**), 132.3 (**C7**), 130.9 (**C5**), 127.6 (**C6 and C9**), 122.4 (**C12**), 74.2 (**C3**), 49.0 (**C2**), 28.8 (**C10**), 24.0 (**C2-CH$_3$**), 20.1 (**C2-CH$_3$**), 20.1 (**C4-CH$_3$**). *The ^{13}C resonances for the pentafluro aromatic ring were not observed in the ^{13}C NMR spectrum.*

IR: 3295 (br, O-H), 3136, 2982 (C-H), 1781 (C=O), 1519 (C=C), 1470, 1231, 1074 (C-O), 993 (C=C, bend), 825, 647

Mass: Calcd. for $C_{22}H_{21}F_5NO_4$ $[M+H]^+$ = 458.1385, found $[M+H]^+$ = 458.1382

(*R*,4*Z*,6*E*,8*E*)-3-Hydroxy-2,2,4-trimethyl-10-(oxazol-5-yl)deca-4,6,8-trienamide (2):

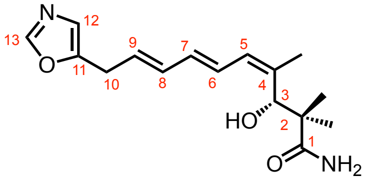

To the stirred solution of ester (**21**, 18 mg, 0.039 mmol) in THF (2 mL) was added NH_4OH solution (70 µl, 9 M in H_2O) under N_2. The resulting mixture was stirred at room temperature for 30 min. The solvent was removed *in vacuo* and the crude residue was purified by column chromatography to give the required inthomycin B (**2**) as colourless oil (10.7 mg, 0.037 mmol, 95 %).

$[\alpha]_D^{25}$ +40.9 (c= 0.82 in $CHCl_3$) (lit.[9] $[\alpha]_D^{26}$ +46.8 (c 1.25, $CHCl_3$), lit.[10] for material of 64%. ee $[\alpha]_D^{22}$ +19.3 (c 1.0, $CHCl_3$))

R$_f$ = 0.4 (5% MeOH/CH_2Cl_2)

^{1}H NMR (500 MHz, $CDCl_3$): δ 7.79 (s, 1H, **H13**), 6.80 (m, 1H, **H12**), 6.42 (dd, *J* = 13.7, 11.3, 1H, **H6**), 6.24 – 6.12 (m, 3H, **H8, 7, NH**), 6.03 (dd, *J* = 11.5, 1.6 Hz, 1H, **H5**), 5.75 (dt, *J* = 13.7, 6.6 Hz, 1H, **H9**), 5.39 (s, 1H, **NH**), 4.62 (d, *J* = 5.5 Hz, 1H, **H3**), 3.90 (d, *J* = 5.6, 1H, **OH**), 3.49 (d, *J* = 6.9, 2H, **H10**), 1.83 – 1.80 (m, 3H, **C4-CH$_3$**), 1.37 (s, 3H, **C2-CH$_3$**), 1.11 (s, 3H, **C2-CH$_3$**)

^{13}C NMR (125 MHz, $CDCl_3$): δ 180.9 (**C1**), 150.7 (**C13**), 150.4 (**C11**), 137.5 (**C4**), 133.3 (**C8**), 132.0 (**C7**), 130.2 (**C5**), 127.4 (**C6**), 127.4 (**C9**), 122.6 (**C12**), 75.9 (**C3**), 44.6 (**C2**), 28.8 (**C10**), 26.1 (**C2-CH$_3$**), 21.7 (**C2-CH$_3$**), 19.2 (**C4-CH$_3$**)

IR: 3341 (br, N-H), 2923 (C-H), 1657 (C=O), 1600 (N-H, bend), 1510 (C=C), 1048 (C-O, alcohol), 773 (C=C, bend)

Mass: Calcd. for $C_{16}H_{22}N_2O_3Na$ $[M+Na]^+$ = 313.1523, found $[M+Na]^+$ = 313.1522.

Methyl (R,4E,6E,8E)-2,2,4-trimethyl-3-((triethylsilyl)oxy)-10-(2-(triisopropylsilyl)oxazol-5-yl)deca-4,6,8-trienoate (22):

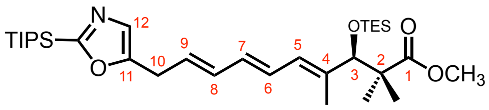

In a 25 mL round bottom flask, boronic ester (**15**, 30 mg, 0.07 mmol) and alkenyl iodide (**(*E*)-17**, 29 mg, 0.08 mmol) were dissolved in THF (1 mL) and purged with argon for 15 mins. Palladium(II) acetate (1.6 mg, 0.007 mmol) and triphenylphosphine (7.5 mg, 0.028 mmol) were added subsequently. Degassed Na_2CO_3 (1.1 mL, 0.25 M) was added and the reaction mixture was stirred at room temperature for 10 h. The crude mixture was absorbed onto silica and purification by flash chromatography gave the coupled product **(22)** as colourless oil (27 mg, 0.047 mmol, 65 %).

$[\alpha]_{\mathrm{D}}^{25}$ + 30.7 (c= 0.15 in $CHCl_3$)

R_f = 0.4 (5% EtOAc/petrol 40-60)

^{1}H NMR (400 MHz, $CDCl_3$): δ 6.84 (t, *J* = 1.0 Hz, 1H, **H12**), 6.38 – 6.30 (m, 1H, **H6**), 6.23 – 6.16 (m, 2H, **H8,7**), 5.98 (d, *J* = 11.1 Hz, 1H, **H5**), 5.77 (dt, *J* = 13.5, 6.9 Hz, 1H, **H9**), 4.36 (s, 1H, **H3**), 3.64 (s 3H, **OCH₃**), 3.51 (d, *J* = 6.7 Hz, 2H, **H10**), 1.73 (s, 3H, **C4-CH₃**), 1.43 – 1.35 (m, 3H, **3** x **CH-TIPS**), 1.14 (m, 21H, **C2-CH₃**, **6** x **CH₃-TIPS**), 1.05 (s, 3H, **C2-CH₃**), 0.91 (t, *J* = 7.84 Hz, 9H, **3** x **CH₃-TES**), 0.53 (q, *J* = 7.82 Hz, 6H, **3** x **CH₂-TES**)

^{13}C NMR (100 MHz, $CDCl_3$): δ 177.3 (**C1**), 167.7 (**C13**), 152.5 (**C11**), 138.1 (**C4**), 133.1 (**C8**), 131.8 (**C7**), 128.1 (**C5**), 128.0 (**C6**), 127.7 (**C9**), 122.8 (**C12**), 81.8 (**C3**), 51.6 (**O<u>C</u>H3**), 49.3 (**C2**), 29.1 (**C10**), 21.7 (**C2-<u>C</u>H₃**), 20.9 (**C2-<u>C</u>H₃**), 18.4 (**C-TIPS**), 14.5 (**C4-<u>C</u>H₃**), 10.9 (**C-TIPS**), 6.8 (**C-TES**), 4.7 (**C-TES**)

IR: 2987 (C-H), 2869, 1740 (C=O), 1593 (C=C), 1464, 1385, 1244, 1130 (C-O, ester), 1076 (C-O, alcohol), 986 (C=C, bend), 883, 843, 726, 676, 657

Mass: Calcd. for $C_{32}H_{58}NO_4Si_2$ $[M+H]^+$ = 576.3899, found $[M+H]^+$ = 576.3902

Methyl (*R*,4*E*,6*E*,8*E*)-3-hydroxy-2,2,4-trimethyl-10-(oxazol-5-yl)deca-4,6,8-trienoate (23):

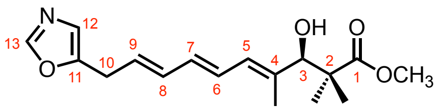

In the same manner as described for the synthesis compound **20**, substrate (**22**, 23 mg, 0.04 mmol) gave the required product (**23**, 12 mg, 0.038 mmol) as a colorless oil in 97% yield after purification by flash chromatography.

$[\alpha]_D^{25}$ + 3.1 (c= 0.27 in $CHCl_3$); (lit.[9] $[\alpha]_D^{22}$ +0.78 (c 1.39 $CHCl_3$), lit.[10] for material of 76% ee $[\alpha]_D^{22}$ +5.2 (c 1.55, $CHCl_3$), lit.[11] $[\alpha]_D^{25}$ +1.5 (c 1.0, $CHCl_3$),)

R_f = 0.2 (30% EtOAc/petrol 40-60)

^{1}H NMR (400 MHz, $CDCl_3$): δ 7.78 (s, 1H, **H13**), 6.79 (s, 1H, **H12**), 6.42 – 6.35 (m, 1H, **H6**), 6.26 – 6.18 (m, 2H, **H8,7**), 6.02 (d, *J* = 10.9, 1H, **H5**), 5.79 – 5.70 (m, 1H, **H9**), 4.17 (d, *J* = 5.5 Hz, 1H, **H3**), 3.70 (s, 3H, **OCH₃**), 3.48 (d, *J* = 6.9 Hz, 2H, **H10**), 3.09 (d, *J* = 5.5 Hz, 1H, **OH**), 1.74 (d, *J* = 1.3 Hz, 3H, **C4-CH₃**), 1.20 (s, 3H, **C2-CH₃**), 1.15 (s, 3H, **C2-CH₃**)

^{13}C NMR (100 MHz, $CDCl_3$): δ 178.2 (**C1**), 150.8 (**C11**), 150.4 (**C13**), 137.3 (**C4**), 133.5 (**C8**), 132.2 (**C7**), 128.5 (**C5**), 128.1 (**C6**), 127.3 (**C9**), 122.5 (**C12**), 82.2 (**C3**), 52.1 (**OCH3**), 47.0 (**C2**), 28.9 (**C10**), 23.7 (**C2-CH₃**), 20.8 (**C2-CH₃**), 14.0 (**C4-CH₃**)

IR: 3378(br, O-H), 2950 (C-H), 1725 (C=O), 1510 (C=C), 1468, 1434, 1385, 1258, 1191, 1129 (C-O, ester), 1046 (C-O, alcohol), 987 (C=C, bend), 823, 647

Mass: Calcd. for $C_{17}H_{23}NO_4Na$ $[M+Na]^+$ = 328.1519, Obtained $[M+Na]^+$ = 328.1525.

Perfluorophenyl (*R*,4*E*,6*E*,8*E*)-3-hydroxy-2,2,4-trimethyl-10-(oxazol-5-yl)deca-4,6,8-trienoate (24):

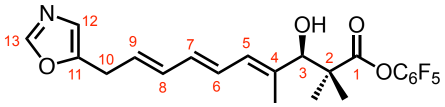

In the same manner as described for the synthesis of compound **21**, substrate (**23**, 18 mg, 0.06 mmol) gave the required product (**24**, 24 mg, 0.052 mmol) as a colorless oil in 87% yield after purification by column chromatography.

$[\alpha]_D^{25}$ +14.7 (c= 0.25 in $CHCl_3$)

R_f = 0.5 (20% EtOAc/petrol 40-60)

^{1}H NMR (400 MHz, $CDCl_3$): δ 7.84 (s, 1H, **H13**), 6.83 (s, 1H, **H12**), 6.43 (td, *J* = 11.0, 3.3 Hz, 1H, **H6**), 6.30 – 6.20 (m, 2H, **H8, 7**), 6.14 (d, *J* = 11.0 Hz, 1H, **H5**), 5.85 – 5.70 (m, 1H, **H9**), 4.51 (d, *J* = 4.1 Hz,

1H, **H3**), 3.51 (d, J = 6.9 Hz 2H, **H10**), 2.40 (d, J = 4.4 Hz, 1H, **OH**), 1.86 (d, J = 1.2 Hz, 3H, **C4-CH$_3$**), 1.35 (d, J = 5.7 Hz, 6H, **2** x **C2-CH$_3$**).

^{13}C NMR (100 MHz, $CDCl_3$): δ 173.4 (**C1**), 150.8 (**C11**), 150.5 (**C13**), 136.3 (**C4**), 133.4 (**C8**), 132.8 (**C7**), 129.3 (**C5**), 129.1 (**C6**), 127.8 (**C9**), 122.5 (**C12**), 80.9 (**C3**), 48.8 (**C2**), 28.9 (**C10**), 23.3 (**C2-C̲H$_3$**), 19.9 (**C2-C̲H$_3$)**, 14.7 (**C4-C̲H$_3$**). *The ^{13}C NMR spectrum of **22** indicated a number of other small resonances particularly in the aromatic region, which are likely due to the pentaflouro aromatic ring.*

IR: 3295 (br, OH), 2922 (C-H), 2668, 2460, 1780 (C=O), 1518 (C=C), 1469, 1231, 1070 (C-O, alcohol), 991 (C=C, bend), 827, 647

Mass: Calcd. for $C_{22}H_{21}OF_5NO_4$ $[M+H]^+$ = 458.1385, found $[M+H]^+$ = 458.1387

(*R*,4*E*,6*E*,8*E*)-3-hydroxy-2,2,4-trimethyl-10-(oxazol-5-yl)deca-4,6,8-trienamide (3):

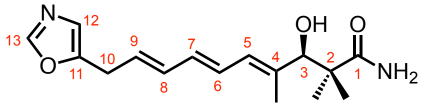

In the same manner as described for the synthesis compound **2**, substrate (**24**, 19.3 mg, 0.04 mmol) gave the required product (**3**, 11.4 mg, 0.039 mmol) as colorless oil in 94% yield after purification by column chromatography.

$[\alpha]_D^{25}$ – 4.0 (c= 0.89 in $CHCl_3$) (lit.[12] $[\alpha]_D$ -7.9 (c 0.33, $CHCl_3$);lit.[12-13] $[\alpha]_D$ −8.4 (c 1.0, $CHCl_3$). lit.[11] $[\alpha]_D$ −8.2 (c 1.0, $CHCl_3$)). *For the assignment of the absolute configuration of inthomycin C along with a discussion of the various reported optical rotations of inthomycin C see ref 12.*

R_f = 0.4 (5% MeOH/CH_2Cl_2)

^{1}H NMR (400 MHz, $CDCl_3$): δ 7.78 (s, 1H, **H13**), 6.79 (s, 1H, **H12**), 6.38 (dd, J = 13.7, 11.3 Hz, 1H, **H6**), 6.31 – 6.13 (m, 3H, **H8, 7, NH**), 6.02 (d, J = 10.9, 1H, **H5**), 5.76 (dt, J = 13.7, 6.8 Hz, 1H, **H9**), 5.56 (br, 1H, **NH**), 4.01 (d, J = 4.9 Hz, 1H, **H3**), 3.90 (d, J = 5.0 Hz, 1H, **OH**), 3.48 (d, J = 6.8 Hz, 2H, **H10**), 1.79 (s, 3H, **C4-CH$_3$**), 1.30 (s, 3H, **C2-CH$_3$**), 1.10 (s, 3H, **C2-CH$_3$**)

^{13}C NMR (125 MHz, $CDCl_3$): δ 180.7 (**C1**), 150.8 (**C11**), 150.4 (**C13**), 137.9 (**C4**), 133.4 (**C8**), 132.3 (**C7**), 128.7 (**C6**), 128.0 (**C5**), 127.4 (**C9**), 122.5 (**C12**), 83.7 (**C3**), 45.0 (**C2**), 28.8 (**C10**), 25.7 (**C2-C̲H$_3$**), 21.7 (**C2-C̲H$_3$**), 13.3 (**C4-C̲H$_3$**)

IR: 3339 (br, NH), 2981 (C-H), 1657, 1599, 1510 (C=C), 1473, 1382, 1252, 1154, 1085, 989 (C=C, bend), 965, 822, 647

Mass: Calcd. for $C_{16}H_{22}N_2O_3Na$ $[M+Na]^+$ = 313.1523, Obtained $[M+Na]^+$ = 313.1524

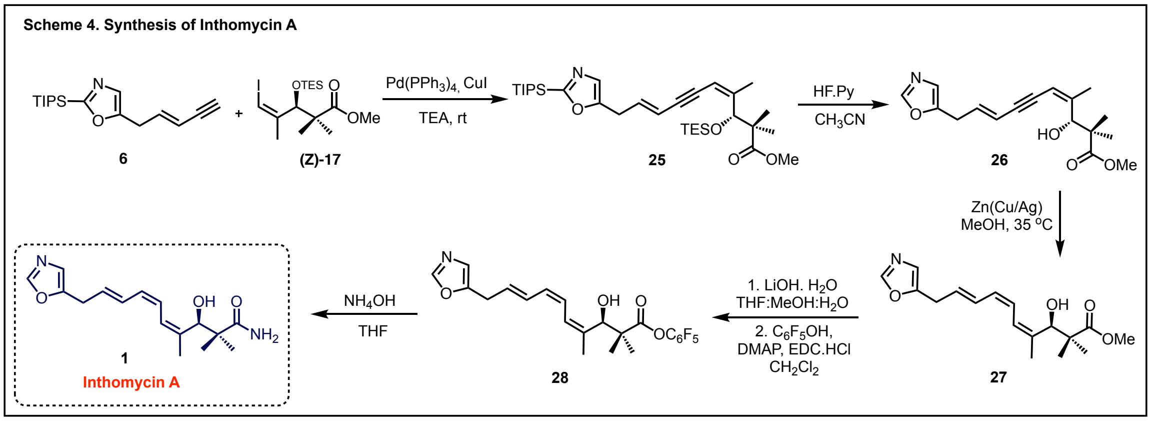

Methyl (R,4Z,8E)-2,2,4-trimethyl-3-((triethylsilyl)oxy)-10-(2-(triisopropylsilyl)oxazol-5-yl)deca-4,8-dien-6-ynoate (25):

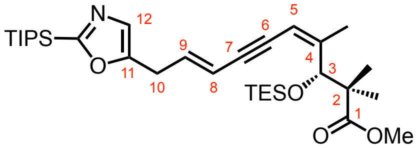

In 25 mL round bottom flask, alkyne (**6**, 43 mg, 0.15 mmol) and alkenyl iodide (**(*Z*-17)**, 45 mg, 0.11 mmol) were dissolved in EtOAc and dried under the vacuum. To the completely dry reaction mixture triethylamine (1.5 mL), purged with argon for 15 mins, was added to the reaction flask. The reaction mixture was degassed (vacuum/Ar, 5-10 cycles). Tetrakis(triphenylphosphine) palladium(0) (8 mg, 0.007 mmol) and copper(I) iodide (6.3 mg, 0.03 mmol) were subsequently added. The degassing procedure was repeated (5-10 cycles). The flask was then sealed with a glass stopper and stirred in the dark for 4 h. The reaction mixture was diluted with Et_2O and sat. brine solution was added. The aqueous phase was extracted with Et_2O (3 x 10 mL). The combined organic phases were dried (Na_2SO_4) and concentrated *in vacuo*. Flash chromatography gave the required product (**25**) as colourless oil (39 mg, 0.07 mmol, 62 %).

$[\alpha]_D^{25}$ +82.8 (c= 0.83 in $CHCl_3$)

R_f = 0.3 (20% EtOAc/petrol 40-60)

^{1}H NMR (400 MHz, $CDCl_3$): δ 6.87 (d, *J* = 1.1 Hz, 1H, **H12**), 6.17 (dt, *J* = 15.8, 6.7 Hz, 1H, **H9**), 5.73 (dq, *J* = 15.8, 1.8 Hz, 1H, **H8**), 5.53 (s, 1H, **H5**), 5.16 (s, 1H, **H3**), 3.63 (s, 3H, **OCH_3**), 3.54 (d, *J* = 6.9, 2H, **H10**), 1.81 (d, *J* = 1.6 Hz, 3H, **C4-CH_3**), 1.44 – 1.34 (m, 3H, **3** x **CH-TIPS**), 1.20 (s, 3H, **C2-CH_3**), 1.16 (s, 3H, **C2-CH_3**), 1.12 (d, *J* = 7.5 Hz, 18H, **6** x **CH_3-TIPS**), 0.93 (t, *J* = 7.9 Hz, 9H, **3** x **CH_3-TES**), 0.56 (q, *J* = 8.1 Hz, 6H, **3** x **CH_2-TES**).

^{13}C NMR (100 MHz, $CDCl_3$): δ 177.0 (**C1**), 168.1 (**C13**), 151.4 (**C11**), 151.0 (**C4**), 136.8 (**C9**), 123.2 (**C12**), 112.8 (**C8**), 109.2 (**C5**), 92.0 (**C7/6**), 86.9 (**C7/6**), 76.6 (**C3**), 51.7 (**OCH_3**), 49.2 (**C2**), 29.7 (**C10**), 29.3 (**C2-CH_3**), 22.5 (**C2-CH_3**), 20.7 (**C4-CH_3**), 18.8 (**C-TIPS**), 10.7 (**C-TIPS**), 6.8(**C-TES**), 4.4 (**C-TES**)

IR: 2949 (C-H), 1736 (C=O), 1593 (C=C), 1465, 1366, 1256 (Si-C), 1192, 1132 (C-O, ester), 1081, 1016 (C-O, alcohol), 953, 884, 770, 741, 677

Mass: Calcd. for $C_{32}H_{56}NO_4Si_2$ $[M+H]^+$ = 574.3742, found $[M+H]^+$ = 574.3740

Methyl (R,4Z,8E)-3-hydroxy-2,2,4-trimethyl-10-(oxazol-5-yl)deca-4,8-dien-6-ynoate (26):

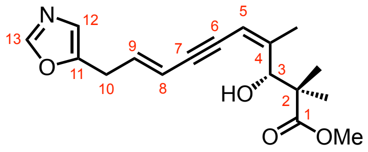

In the same manner as described for the synthesis compound **20**, substrate (**25**, 44 mg, 0.076 mmol) gave the required product (**26**, 21 mg, 0.069 mmol) as colorless oil in 91% yield after purification through column chromatography.

$[\alpha]_D^{25}$ – 26.1 (c= 0.6 in $CHCl_3$)

R_f = 0.2 (20% EtOAc/petrol 40-60)

^{1}H NMR (500 MHz, $CDCl_3$): δ 7.80 (s, 1H, **H13**), 6.84 (s, 1H, **H12**), 6.12 (dt, *J* = 15.8, 6.8 Hz, 1H, **H9**), 5.74 (dq, *J* = 15.7, 1.9 Hz, 1H, **H8**), 5.58 (s, 1H, **H5**), 4.83 (d, *J* = 7.2 Hz, 1H, **H3**), 3.72 (s, 3H, **OCH_3**), 3.56 (d, *J* = 7.3 Hz, 1H, **OH**), 3.51 (d, *J* = 6.8, 2H, **H10**), 1.75 (d, *J* = 1.5 Hz, 3H, **C4-CH_3**), 1.34 (s, 3H, **C2-CH_3**), 1.19 (s, 3H, **C2-CH_3**)

^{13}C NMR (125 MHz, $CDCl_3$): δ 178.3 (**C1**), 150.7 (**C11/4**), 149.8 (**C11/4**), 149.5 (**C13**), 136.4 (**C9**), 123.0 (**C12**), 113.1 (**C8**), 109.6 (**C5**), 91.6 (**7/6**), 86.8 (**C7/6**), 76.6 (**C3**), 52.2 (**O<u>C</u>H_3**), 46.5 (**C2**), 29.0 (**C10**), 24.6 (**C2-<u>C</u>H_3**), 20.7 (**C2-<u>C</u>H_3**), 18.4 (**C4-<u>C</u>H_3**). *Note C-5 and C-12 appear as small broad resonances in the ^{13}C NMR of compound **25**.*

IR: 3358 (br, OH), 2951 (C-H), 2188 (C≡C), 1727 (C=O), 1603, 1511, 1470, 1434, 1261, 1194, 1133 (C-O, ester), 1058 (C-O, alcohol), 1010, 954, 829, 647

Mass: Calcd. for $C_{17}H_{21}NNaO_4$ $[M+Na]^+$ = 326.1363, found $[M+Na]^+$ = 326.1362

Methyl (*R*,4*Z*,6*Z*,8*E*)-3-hydroxy-2,2,4-trimethyl-10-(oxazol-5-yl)deca-4,6,8-trienoate (27):

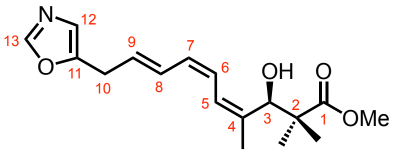

Boland conditions have been used to synthesise the Zn(Cu/Ag) couple.[14]
Zinc Powder was etched with 1 M HCl solution for 1 min and the supernatant acid was removed by pipette. The procedure was repeated twice. The zinc powder was then carefully washed with water, acetone and Et_2O. Dried under an N_2 flow and the resultant zinc dust (2.0 g, 30.5 mmol) was suspended in water (6 mL) and copper(II) acetate (0.2 g, 1.1 mmol) was added and the suspension was stirred for 15 min. Silver nitrate (0.2 g, 1.2 mmol) was added and stirring was

continued for a further 30 min. The Zn(Cu/Ag) couple was collected by suction filtration and carefully washed with water, MeOH and Et_2O respectively.

The ether moist Zn(Cu/Ag) couple was immediately transferred into MeOH:H_2O (1:1, 6 mL) and the substrate (**26**, 60 mg, 0.2 mmol) was added. The reaction mixture heated to 35 °C and stirred for 18 h to achieve full conversion. The reaction mixture was filtered through a short pad of Celite™ and eluted with MeOH (5 mL). The solvent was removed *in vacuo* and purification by flash chromatography gave the required product in 80 % yield (**27**, 48 mg, 0.16 mmol).

$[\alpha]_D^{25}$ +71.8 (c= 0.83 in $CHCl_3$); (lit.[9] $[\alpha]_D$ +100.6 (c 0.7, CH_2Cl_2);lit.[15] $[\alpha]_D$ +102.1 (c 1.0, CH_2Cl_2)).

R_f = 0.3 (20% EtOAc/petrol 40-60)

^{1}H NMR (500 MHz, $CDCl_3$): δ 7.79 (s, 1H, **H13**), 6.80 (s, 1H, **H12**), 6.65 (dd, *J* = 14.5, 11.7 Hz, 1H, **H8**), 6.44 (dd, *J* = 12.0, 1.6 Hz, 1H, **H5**), 6.21 (t, *J* = 11.5 Hz, 1H, **H6**), 5.95 (t, *J* = 11.1 Hz, 1H, **H7**), 5.77 (dt, *J* = 14.3, 6.9 Hz, 1H, **H9**), 4.76 (d, *J* = 6.5 Hz, 1H, **H3**), 3.71 (s, 3H, **OCH₃**), 3.52 (d, *J* = 6.9 Hz, 2H, **H10**), 3.31 (d, *J* = 6.5 Hz, 1H, **OH**), 1.79 (d, *J* = 1.3 Hz, 3H, **C4-CH₃**), 1.25 (s, 3H, **C2-CH₃**), 1.16 (s, 3H, **C2-CH₃**)

^{13}C NMR (125 MHz, $CDCl_3$): δ 178.3 (**C1**), 150.7 (**C11**), 150.4 (**C13**), 137.6 (**C4**), 128.5 (**C9**), 128.2 (**C8**), 127.9 (**C7**), 124.9 (**C5**), 124.1 (**C6**), 122.5 (**C12**), 74.6 (**C3**), 52.2 (**OCH₃**), 46.9 (**C2**), 29.0 (**C10**), 24.4 (**C2-CH₃**), 21.0 (**C2-CH₃**), 19.7 (**C4-CH₃**).

IR: 3394 (br, OH), 2951 (C-H), 1729 (C=O), 1636, 1511, 1471, 1434, 1259, 1192, 1138, 1010, 952, 830, 772, 648

Mass: Calcd. for $C_{17}H_{23}NNaO_4$ $[M+Na]^+$ = 328.1519, Obtained $[M+Na]^+$ = 328.1518

Perfluorophenyl (R,4Z,6Z,8E)-3-hydroxy-2,2,4-trimethyl-10-(oxazol-5-yl)deca-4,6,8-trienoate (28)

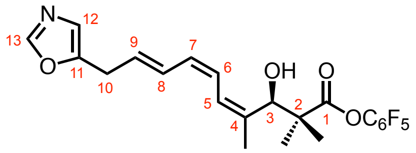

In the same manner as described for the synthesis compound **21**, substrate (**27**, 8.3 mg, 0.028 mmol) gave the required product (**28**, 10.1 mg, 0.022 mmol) as colorless oil in 78 % yield after purification by column chromatography.

$[\alpha]_D^{25}$ + 67.5 (c= 0.28 in $CHCl_3$)

R_f = 0.4 (20% EtOAc/petrol 40-60)

^{1}H NMR (500 MHz, $CDCl_3$): δ 7.80 (s, 1H, **H13**), 6.82 (s, 1H, **H12**), 6.67 (dd, *J* = 14.8, 12.3 Hz, 1H, **H8**), 6.56 – 6.52 (d, *J* = 12.1 Hz, 1H, **H5**), 6.24 (t, *J* = 11.5 Hz, 1H, **H6**), 5.99 (t, *J* = 11.1 Hz, 1H, **H7**), 5.81 (dt, *J* = 14.4, 6.9 Hz, 1H, **H9**), 5.10 (d, *J* = 5.5 Hz, 1H, **H3**), 3.54 (d, *J* = 6.9 Hz, 2H, **H10**), 2.51 (d, *J* = 5.5 Hz, 1H, **OH**), 1.93 (s, 3H, **C4-CH₃**), 1.39 (s, 3H, **C2-CH₃**), 1.37 (s, 3H, **C2-CH₃**)

^{13}C NMR (125 MHz, $CDCl_3$): δ 173.7 (**C1**), 150.8 (**C11**), 150.5 (**C13**), 136.5 (**C4**), 129.0 (**C9**), 128.5 (**C8**), 128.1 (**C7**), 125.7 (**C5**), 123.9 (**C6**), 122.4 (**C12**), 73.8 (**C3**), 49.1 (**C2**), 29.7 (**C-10**), 29.0 (**C2-CH₃**), 24.0 (**C2-CH₃**), 20.2 (**C4-CH₃**). *The ^{13}C NMR spectrum of **27** indicated a number of other small resonances particularly in the aromatic region which are likely due to the pentaflouro aromatic ring.*

IR: 3304 (br, OH), 2921 (C-H), 2851, 1779 (C=O), 1519 (C=C), 1470, 1077 (C-O, alcohol), 994, 828, 647

Mass: Calcd. for $C_{22}H_{20}F_5NNaO_4$ $[M+Na]^+$ = 480.1205, found $[M+Na]^+$ = 480.1202

(*R*,4*Z*,6*Z*,8*E*)-3-Hydroxy-2,2,4-trimethyl-10-(oxazol-5-yl)deca-4,6,8-trienamide (1):

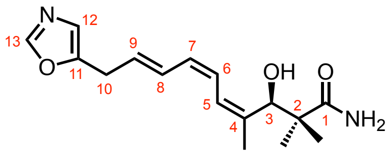

In the same manner as described for the synthesis compound **2**, substrate (**28**, 8.1 mg, 0.018 mmol) gave the required product (**1**, 4.8 mg, 0.016 mmol) as colorless oil in 89 % yield after purification by column chromatography. The synthetic inthomycin A was contaminated with a small quantity of inthomycin B which appears to arise during conversion of the ester **27** into inthomycin A **1**.

$[\alpha]_D^{25}$ + 32.3 (c= 0.42 in $CHCl_3$) (lit.[9] $[\alpha]_D^{21}$ +37.3 (c 0.62, $CHCl_3$); lit.[16] $[\alpha]_D^{21}$ +37.4 (c 1.0, $CHCl_3$))

R_f = 0.4 (5% MeOH/CH_2Cl_2),

^{1}H NMR (500 MHz, $CDCl_3$): δ 7.79 (s, 1H, **H13**), 6.81 (s, 1H, **H12**), 6.65 (dd, *J* = 12.6, 2.8 Hz, 1H, **H8**), 6.43 (d, *J* = 11.9 Hz, 1H, **H5**), 6.29 – 6.19 (m, 2H, **H6, NH**), 5.96 (t, J = 10.9 Hz, 1H, **H7**), 5.78 (dt, *J* = 14.5, 6.9 Hz, 1H, **H9**), 5.49 (s, 1H, **NH**), 4.65 (d, *J* = 5.9 Hz, 1H, **H3**), 4.05 (d, *J* = 5.9 Hz, 1H, **OH**), 3.52 (d, *J* = 6.9 Hz, 2H, **H10**), 1.86 (d, *J* = 1.3 Hz, 3H, **C4-CH₃**), 1.36 (s, 3H, **C2-CH₃**), 1.10 (s, 3H, **C2-CH₃**)

^{13}C NMR (125 MHz, $CDCl_3$): δ 180.9 (**C1**), 150.7 (**C11**), 150.4 (**C13**), 138.3 (**C4**), 128.7 (**C9**), 128.2 (**C8**), 128.1 (**C7**), 124.9 (**C5**), 123.7 (**C6**), 122.5 (**C12**), 75.4 (**C3**), 44.6 (**C2**), 29.0 (**C10**), 26.1 (**C2-CH₃**), 21.7 (**C2-CH₃**), 19.4 (**C4-CH₃**)

IR: 3338 (br, NH), 2964 (C-H), 2923, 1657, 1600, 1510, 1470, 1378, 1087, 1044, 995, 823, 753, 647

Mass: Calcd. for $C_{16}H_{22}N_2NaO_3$$[M+Na]^+$ = 313.1523, found $[M+Na]^+$ = 313.1523

Methyl (S,Z)-5-iodo-2,2,4-trimethyl-3-(((S)-3,3,3-trifluoro-2-methoxy-2-phenylpropan- oyl)oxy) pent-4-enoate (SI-2):

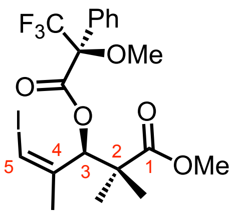

To a stirred solution of the (*Z*)-alcohol (**(*Z*)-5**, 40 mg, 0.14 mmol) in dry CH_2Cl_2 (3 mL) at room temperature was successively added (S)-α-methoxy-α-trifluoromethylphenylacetic acid (36 mg, 0.15 mmol), *N*,*N*-dicyclohexylcarbodiimide (28 mg, 0.14 mmol) and 4-(dimethylamino)pyridine (2 mg, 0.016 mmol). The reaction mixture was stirred under the N_2 balloon for 6 days. The reaction mixture filtered through Celite™ and the residue was washed with CH_2Cl_2. The filtrates and washings were combined and concentrated in *vacuo*. The crude product mixture was purified by silica-gel chromatography to give **SI-2** (21 mg, 0.04 mmol) as a colorless oil in 30% yield.

R_f = 0.8 (10% EtOAc/petrol 40-60)

^{1}H NMR (400 MHz, $CDCl_3$) δ 7.51 – 7.46 (m, 2H, **Ph**), 7.42 (m, 3H, **Ph**), 6.46 (d, *J* = 1.6 Hz, 1H, **H5**), 6.21 (s, 1H, **H3**), 3.61 (s, 3H, **OMe**), 3.49 (s, 3H, **OMe**), 1.80 (s, 3H, **C4-CH$_3$**), 1.30 (s, **C2-CH$_3$**), 1.28 (s, **C2-CH$_3$**)

^{13}C NMR (100 MHz, $CDCl_3$) δ 175.2, 165.2, 140.6, 129.7, 128.4, 127.6, 83.1, 83.0, 55.3, 52.3, 47.4, 23.2, 21.1, 20.4

IR: 2950 (C-H), 1745 (C=O, ester), 1666 (C=C), 1451, 1245 (C-O), 1168, 1120, 1012 (C-O), 950, 720, 607

Mass: Calcd. for $C_{19}H_{22}O_5F_3INa$ $[M+Na]^+$: 537.0356; found 537.0352

Methyl (S,Z)-5-iodo-2,2,4-trimethyl-3-(((R)-3,3,3-trifluoro-2-methoxy-2-phenylpropanoyl)oxy) pent-4-enoate (SI-3):

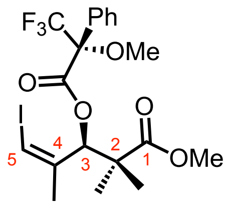

In the same manner as described for the synthesis compound **SI-2**, substrate (**(*Z*)-5**, 40 mg, 0.14 mmol) and (*R*)-α-methoxy-α-trifluoromethylphenylacetic acid (36 mg, 0.15 mmol) gave the required product (**SI-3**, 18 mg, 0.03 mmol) as colorless oil in 26% yield after purification by column chromatography.

R_f = 0.8 (10% EtOAc/petrol 40-60)

^{1}H NMR (400 MHz, $CDCl_3$) δ 7.50 (dd, *J* = 6.9, 2.9 Hz, 2H, **Ph**), 7.39 (dd, *J* = 5.0, 1.9 Hz, 3H, **Ph**), 6.43 (d, *J* = 1.6 Hz, 1H, **H5**), 6.21 (s, 1H, **H3**), 3.65 (s, 3H, **OMe**), 3.52 (d, *J* = 1.4 Hz, 3H. **OMe**), 1.66 (d, *J* = 1.4 Hz, 3H, **C4-CH$_3$**), 1.32 (s, 3H, **C2-CH$_3$**), 1.30 (s, 3H, **C2-CH$_3$**)

^{13}C NMR (100 MHz, $CDCl_3$) δ 175.4, 165.2, 140.6, 129.6, 128.4, 127.4, 83.1, 83.0, 55.5, 52.4, 47.3, 23.4, 20.8, 20.1

Mass: Calcd. for $C_{19}H_{22}O_5F_3INa$ $[M+Na]^+$: 537.0356; found 537.0359

Methyl (S,E)-5-iodo-2,2,4-trimethyl-3-(((S)-3,3,3-trifluoro-2-methoxy-2-phenylpropanoyl)oxy) pent-4-enoate (SI-4):

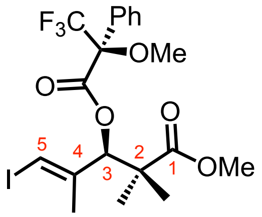

To a stirred solution of the (*E*)-alcohol (**(*E*)-5**, 40 mg, 0.14 mmol) in dry CH_2Cl_2 (3 mL) at room temperature was successively added (*S*)-α-methoxy-α-trifluoromethylphenylacetic acid (36 mg, 0.15 mmol), N,N-dicyclohexylcarbodiimide (28 mg, 0.14 mmol) and 4-(dimethylamino)pyridine (2 mg, 0.016 mmol). The reaction mixture flushed with N_2 and covered with the glass lid, stirred for 10 days. The reaction mixture was filtered through Celite™ and the residue as washed with CH_2Cl_2. The filtrates and washings were combined and concentrated in *vacuo*. The crude product mixture was purified by silica-gel chromatography to give **SI-4** (72 mg, 0.13 mmol) as a colorless oil in 95% yield.

R_f = 0.8 (10% EtOAc/petrol 40-60)

^{1}H NMR (400 MHz, $CDCl_3$) δ 7.42 (s, 5H, **Ph**), 6.28 (s, 1H, **H5**), 5.77 (s, 1H, **H3**), 3.61 (s, 3H, **OMe**), 3.45 (d, *J* = 1.4 Hz, 3H, **OMe**), 1.82 (d, *J* = 1.2 Hz, 3H, **C4-CH$_3$**), 1.17 (s, 3H, **C2-CH$_3$**), 1.13 (s, 3H, **C2-CH$_3$**)

^{13}C NMR (100 MHz, $CDCl_3$) δ 175.0, 165.2, 142.5, 129.8, 128.6, 127.5, 83.7, 82.4, 55.2, 52.2, 46.5, 23.3, 22.1, 20.4

Mass: Calcd. for $C_{19}H_{22}O_5F_3INa$ $[M+Na]^+$: 537.0356; found 537.0355

Methyl (S,E)-5-iodo-2,2,4-trimethyl-3-(((R)-3,3,3-trifluoro-2-methoxy-2-phenylpropanoyl) oxy) pent-4-enoate (SI-5):

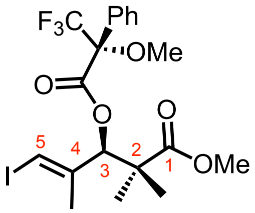

In the same manner as described for the synthesis compound **SI-4**, substrate (***(E)*-5**, 40 mg, 0.14 mmol) and (R)-α-methoxy-α-trifluoromethylphenylacetic acid (36 mg, 0.15 mmol) gave the required product (**SI-5**, 58 mg, 0.11 mmol) as a colorless oil in 77% yield after purification by column chromatography.

R_f = 0.8 (10% EtOAc/petrol 40-60)

^{1}H NMR (400 MHz, $CDCl_3$) δ 7.42 (d, *J* = 5.7 Hz, 5H, **Ph**), 6.15 (d, *J* = 1.6 Hz, 1H, **H5**), 5.72 (s, 1H, **H3**), 3.64 (s, 3H, **OMe**), 3.50 (d, *J* = 1.5 Hz, 3H, **OMe**), 1.78 (s, 3H, **C4-CH$_3$**), 1.20 (s, 3H, **C2-CH$_3$**), 1.15 (s, 3H, **C2-CH$_3$**)

^{13}C NMR (100 MHz, $CDCl_3$) δ 175.2, 165.1, 142.5, 129.7, 128.5, 127.2, 83.4, 82.4, 55.6, 52.3, 46.3, 23.5, 22.5, 19.9

Mass: Calcd. for $C_{19}H_{22}O_5F_3INa$ $[M+Na]^+$: 537.0356; found 537.0355

The absolute configuration of the alcohols **(*Z*)-5** and **(*E*)-5** were assigned using the method of Ohtani[17]

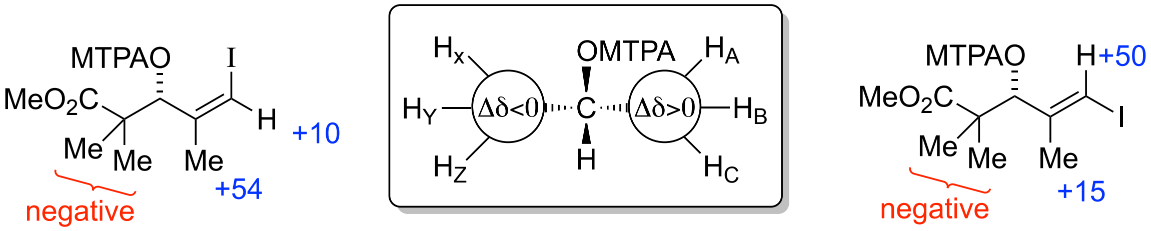

(*E*)-5-(Trimethylsilyl)pent-2-en-4-yn-1-ol (SI-1)

^{1}H NMR (400 MHz, $CDCl_3$)

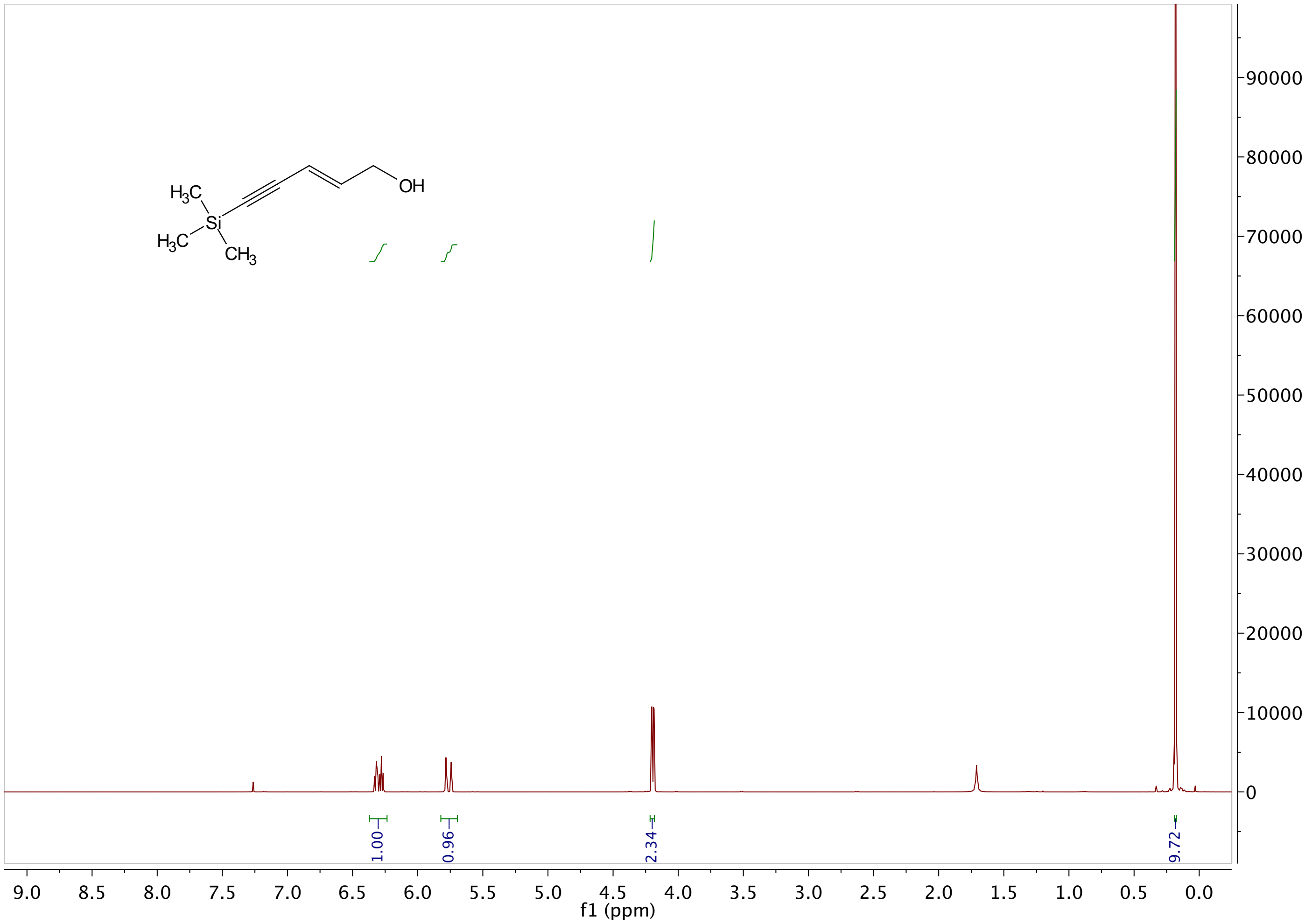

(*E*)-5-(Trimethylsilyl)pent-2-en-4-yn-1-ol (SI-1)

^{13}C NMR (100 MHz, $CDCl_3$)

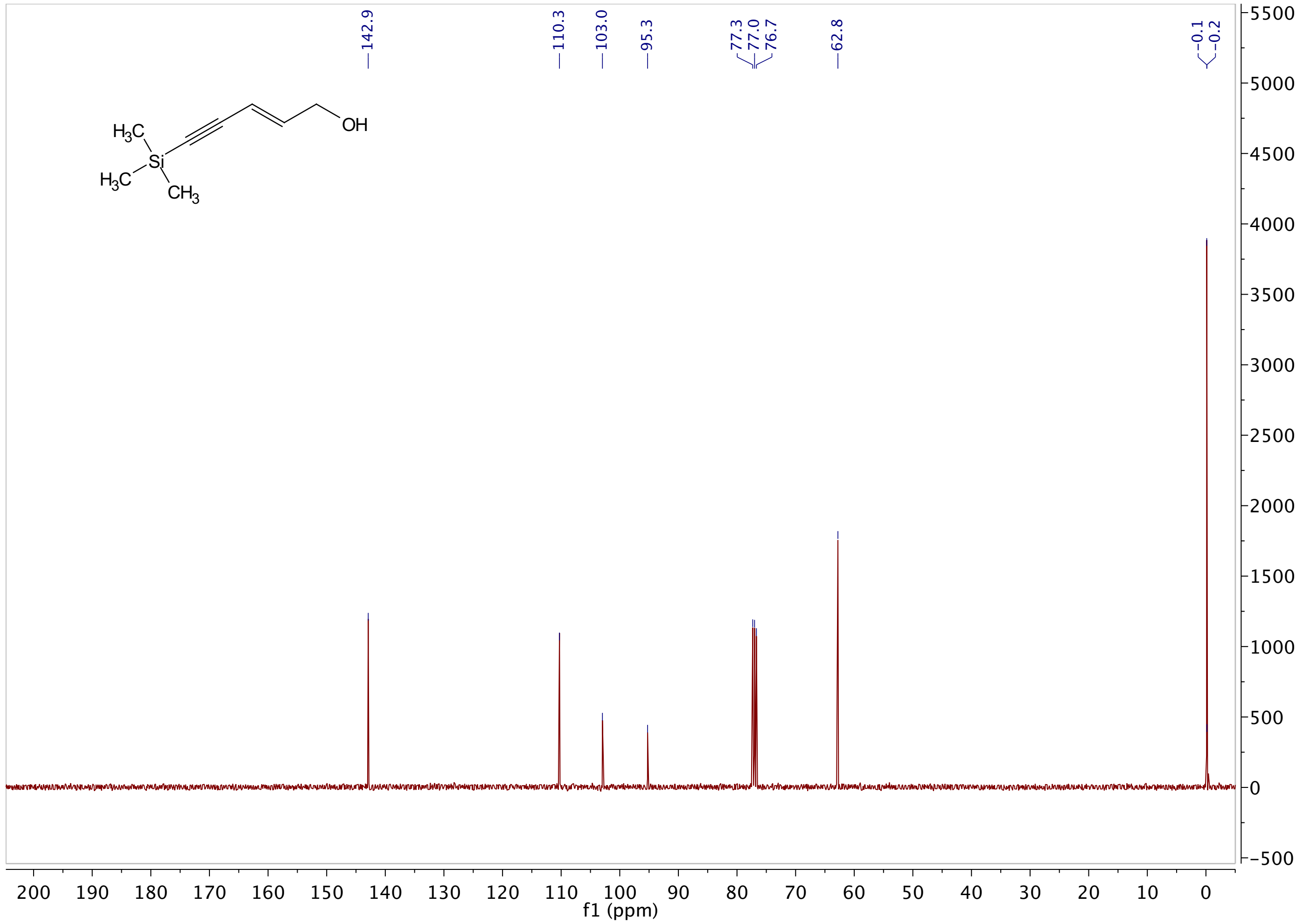

(*E*)-(5-Bromopent-3-en-1-yn-1-yl)trimethylsilane (12)

^{1}H NMR (400 MHz, $CDCl_3$)

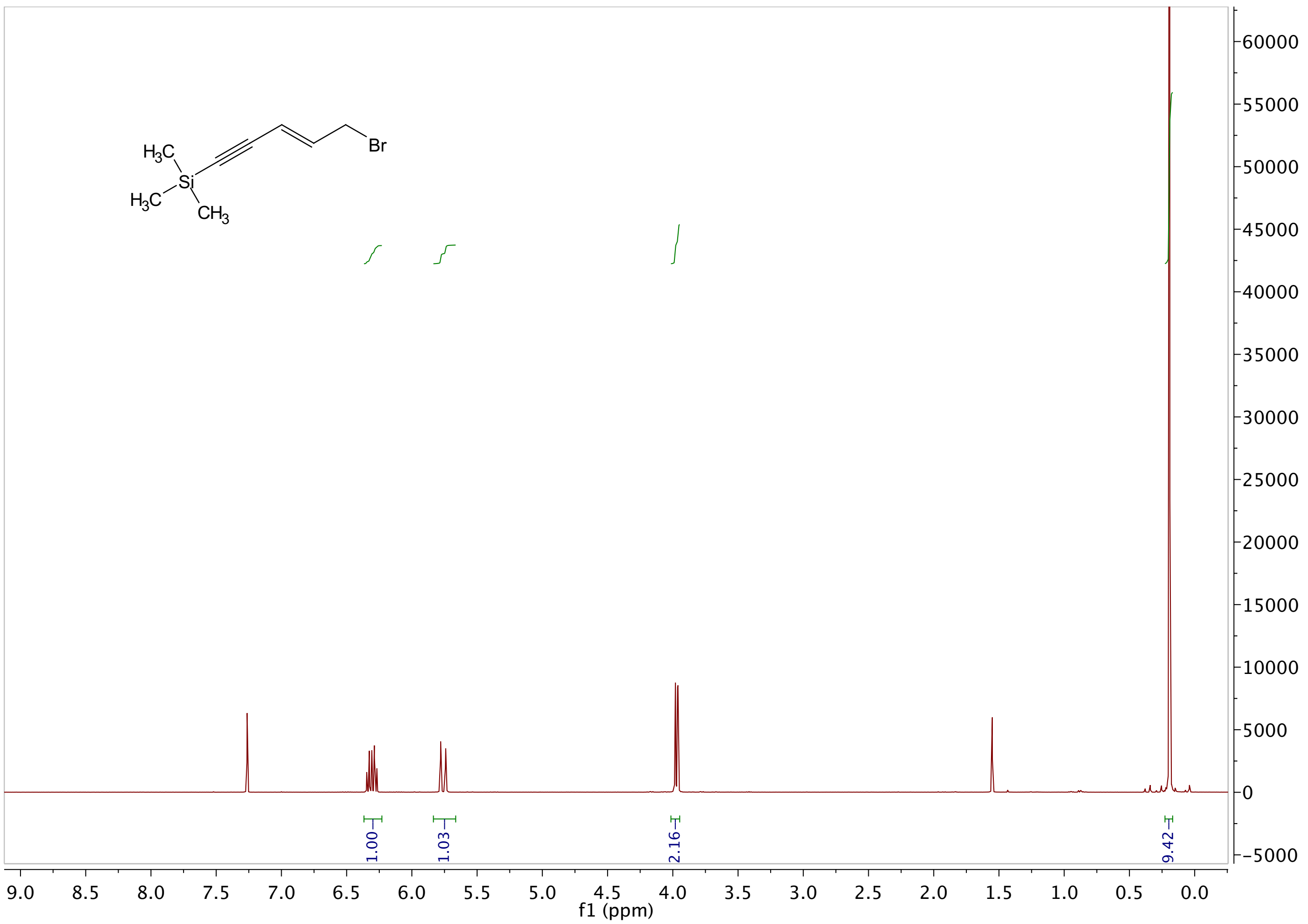

(*E*)-(5-Bromopent-3-en-1-yn-1-yl)trimethylsilane (12)

^{13}C NMR (100 MHz, $CDCl_3$)

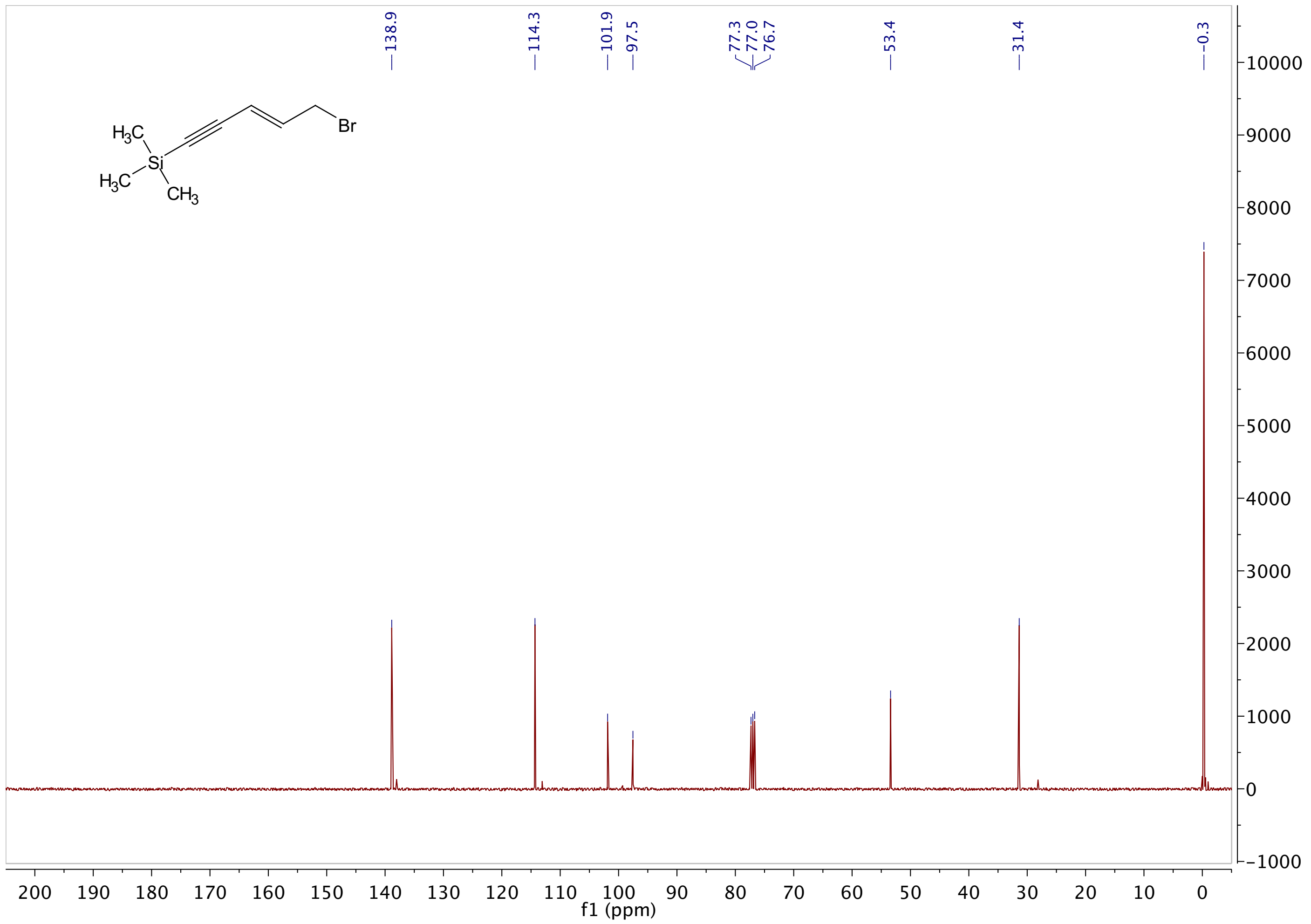

2-(Triisopropylsilyl)oxazole (13)

^{1}H NMR (400 MHz, CDCl$_3$)

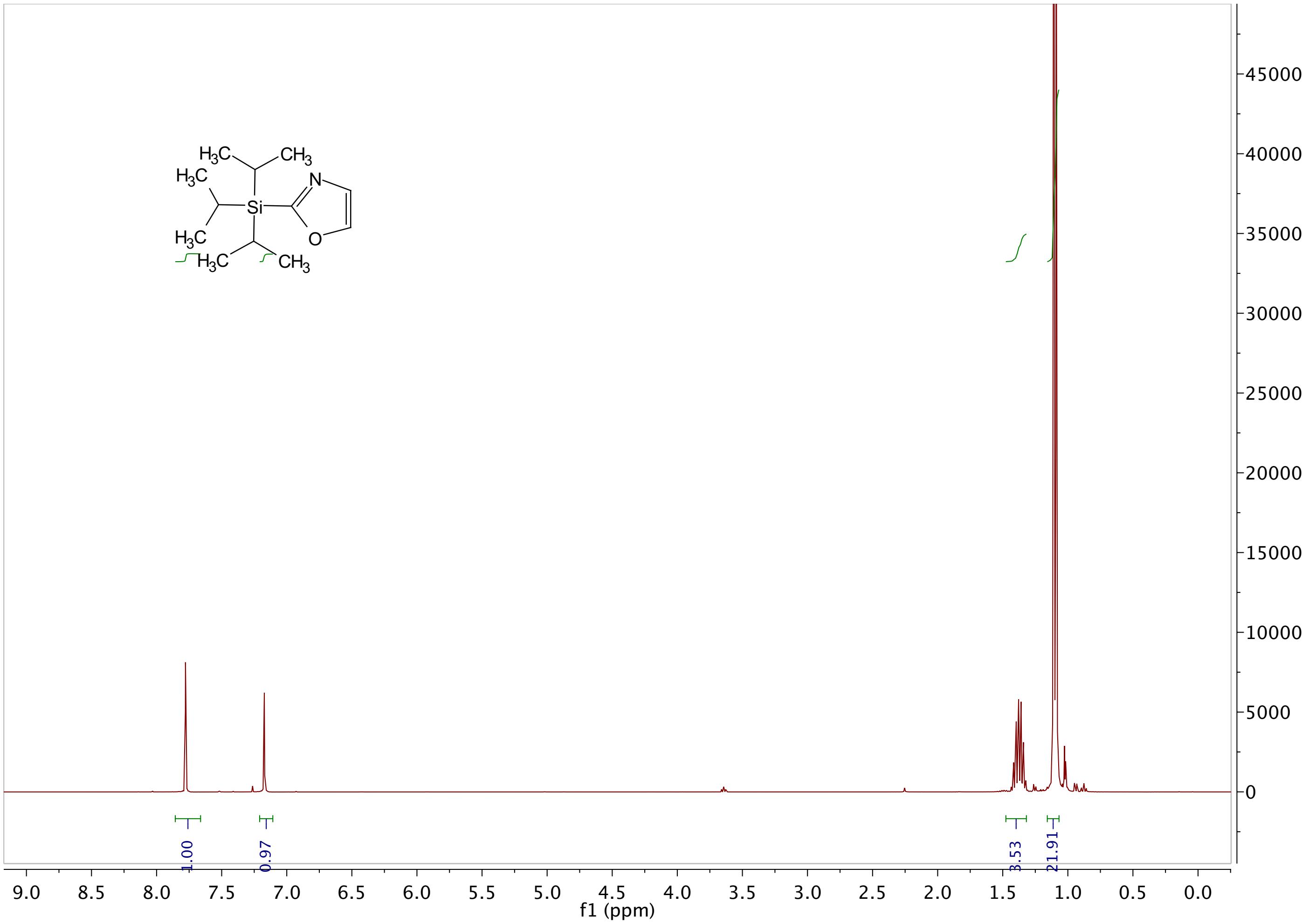

2-(Triisopropylsilyl)oxazole (13)

^{13}C NMR (100 MHz, CDCl$_3$)

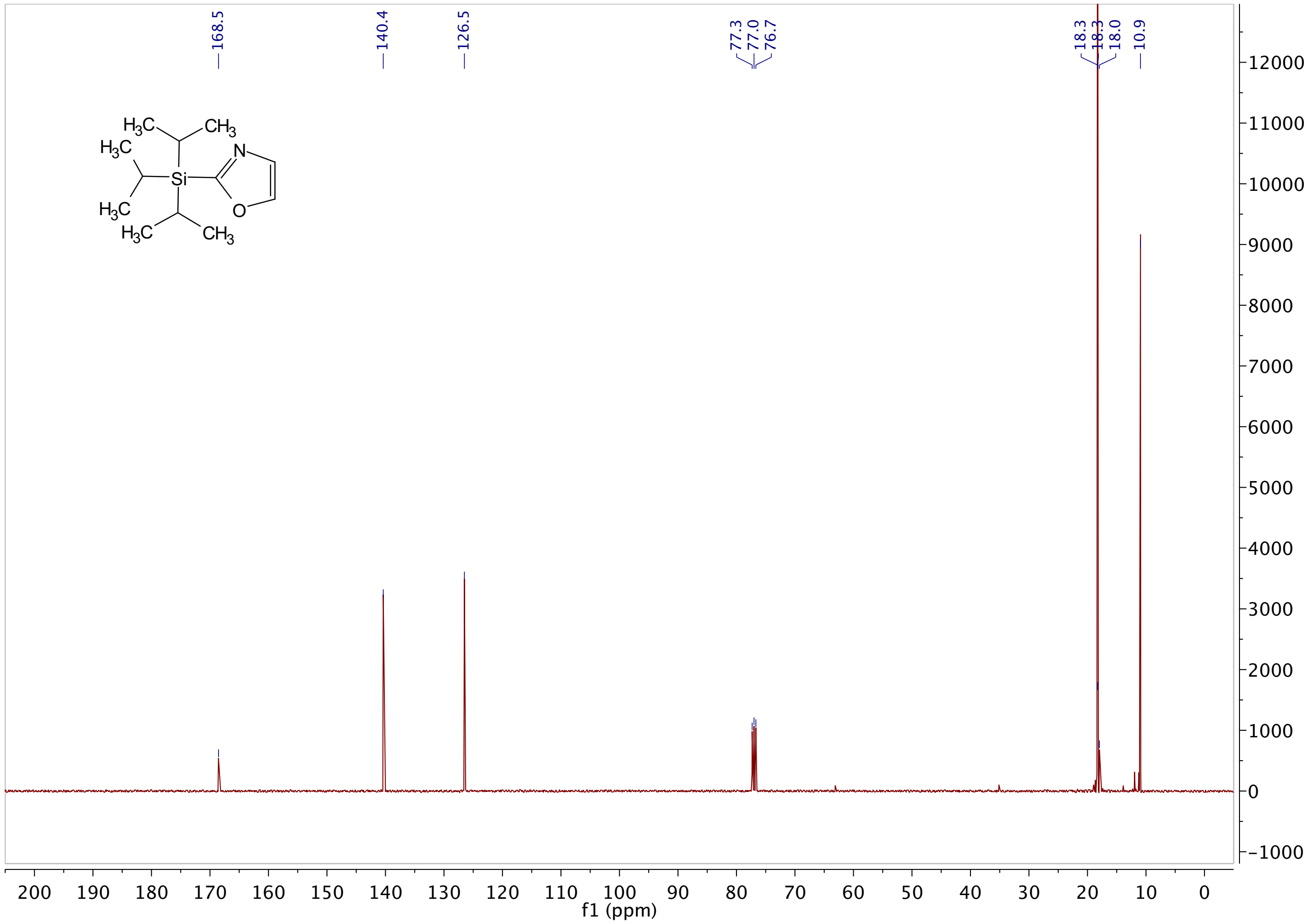

(E)-2-(Triisopropylsilyl)-5-(5-(trimethylsilyl)pent-2-en-4-yn-1-yl)oxazole (14)

^{1}H NMR (400 MHz, $CDCl_3$)

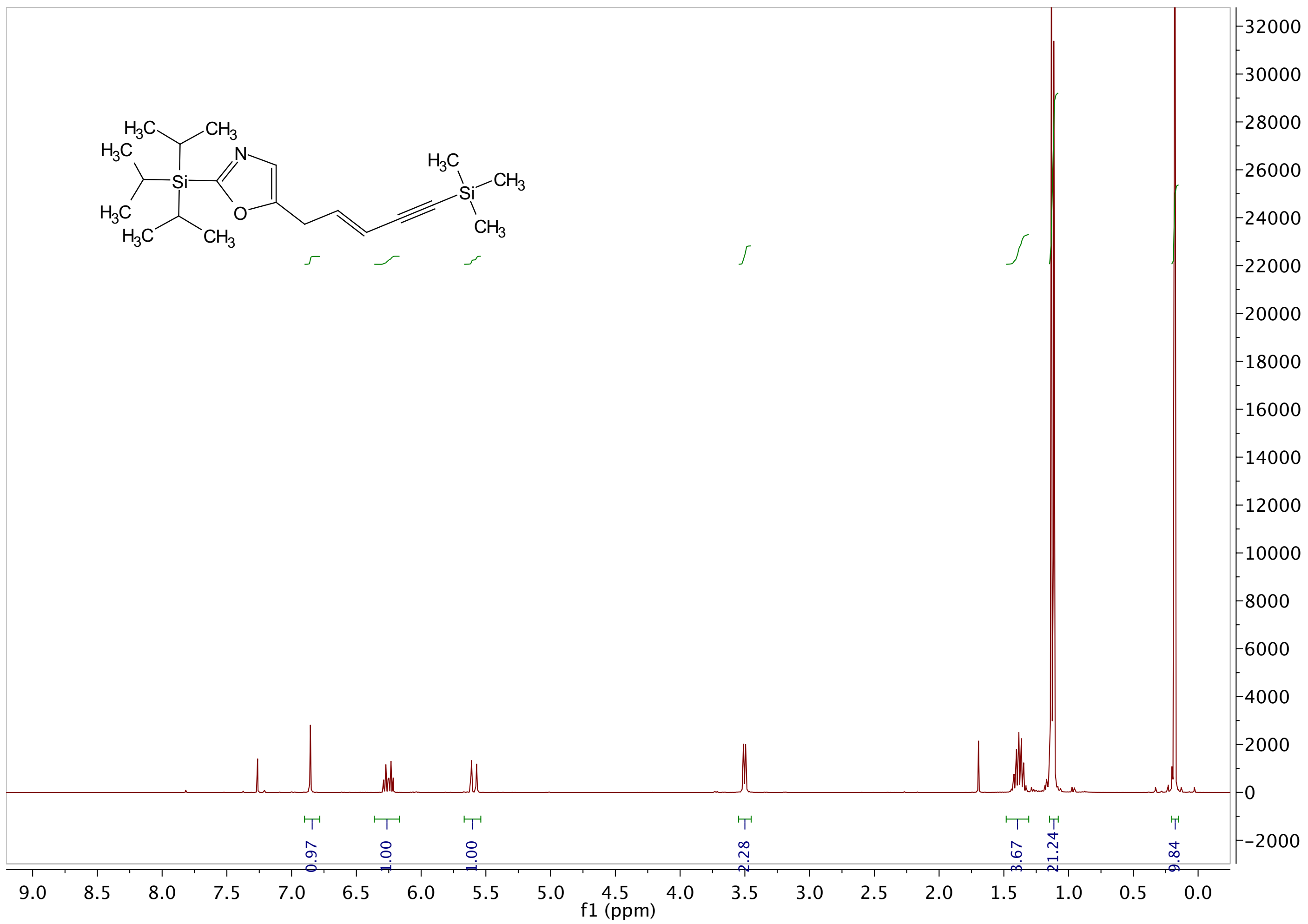

(E)-2-(Triisopropylsilyl)-5-(5-(trimethylsilyl)pent-2-en-4-yn-1-yl)oxazole (14)

^{13}C NMR (100 MHz, $CDCl_3$)

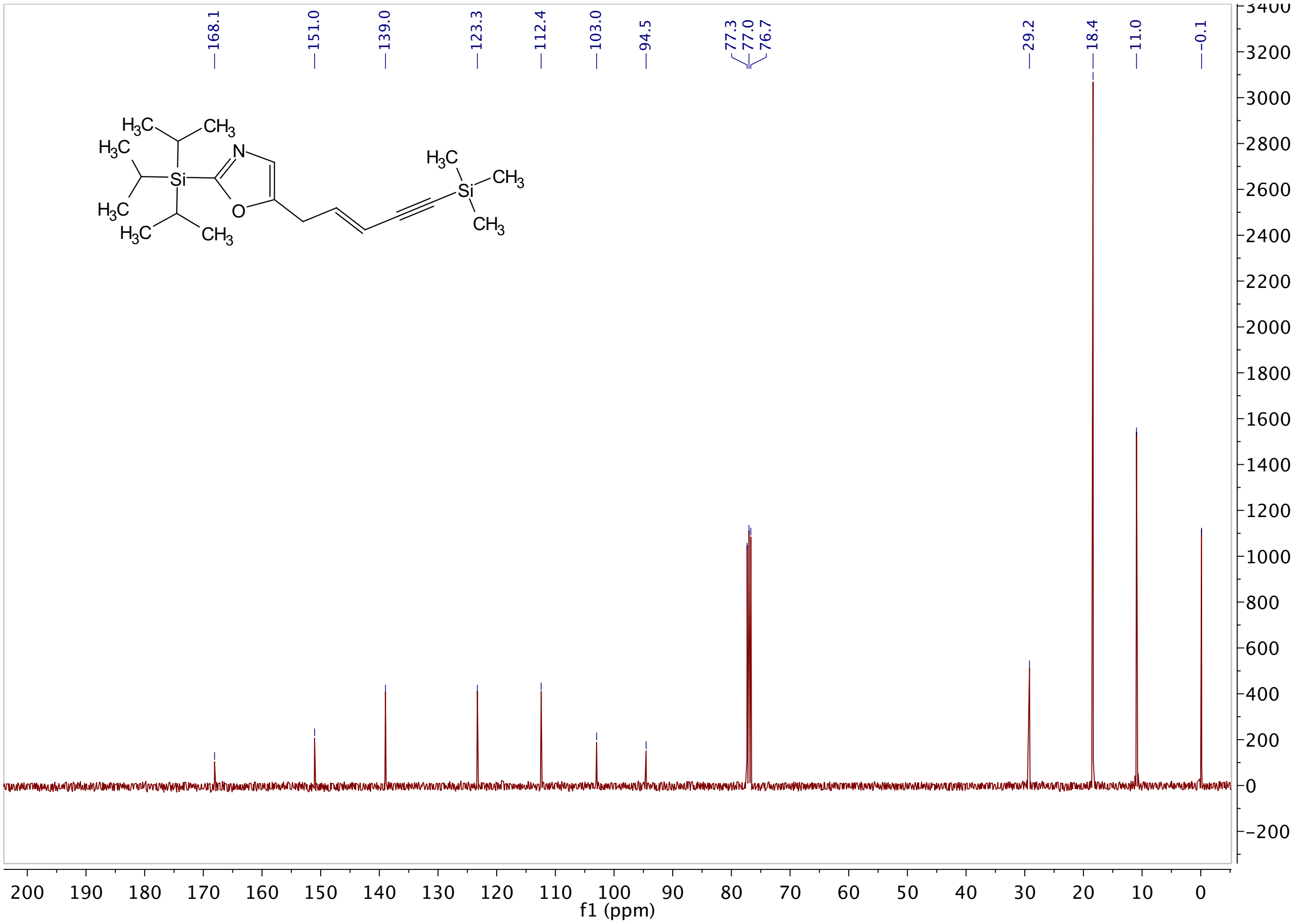

(E)-5-(Pent-2-en-4-yn-1-yl)-2-(triisopropylsilyl)oxazole (6)

^{1}H NMR (400 MHz, $CDCl_3$)

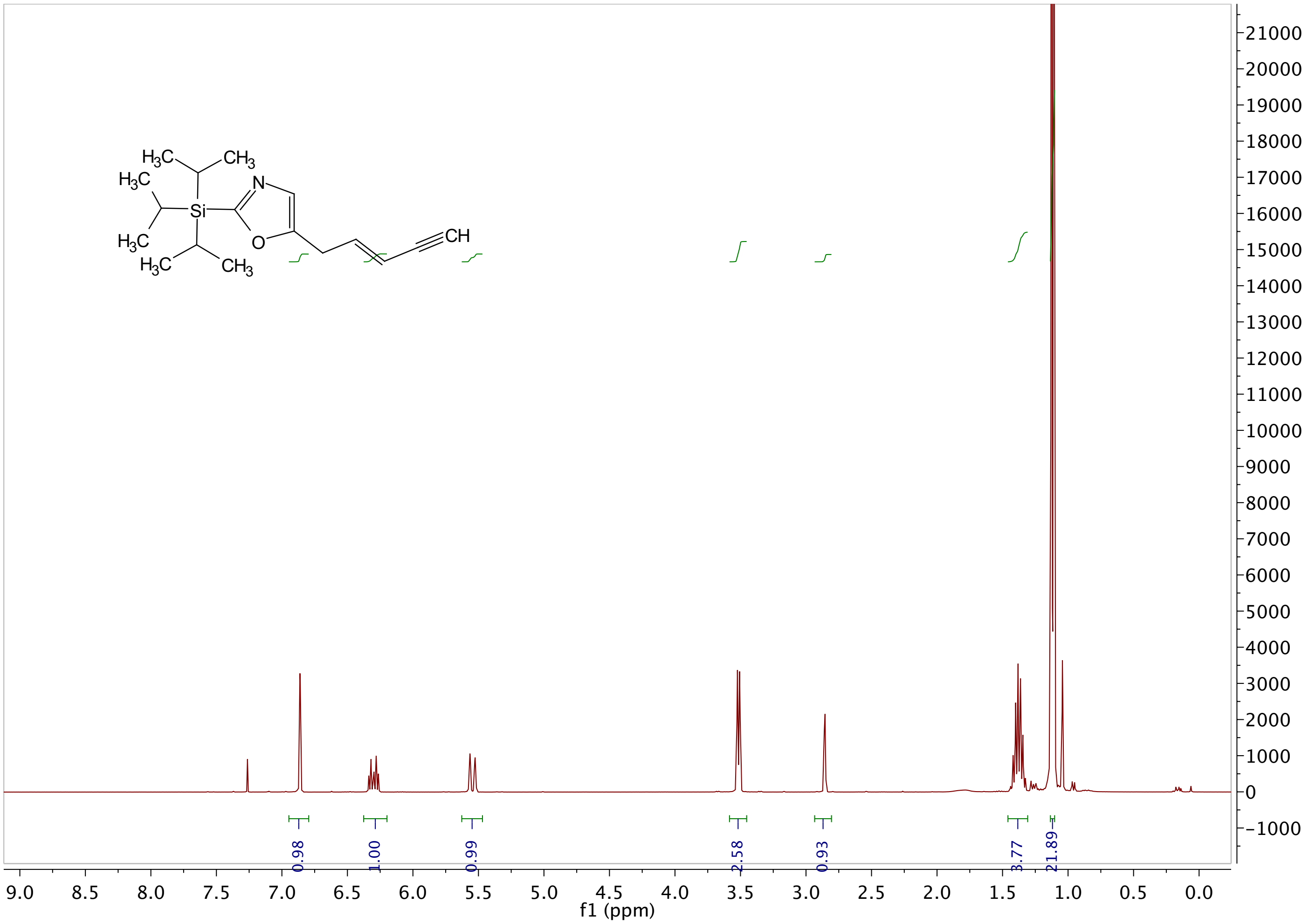

(E)-5-(Pent-2-en-4-yn-1-yl)-2-(triisopropylsilyl)oxazole (6)

^{13}C NMR (100 MHz, $CDCl_3$)

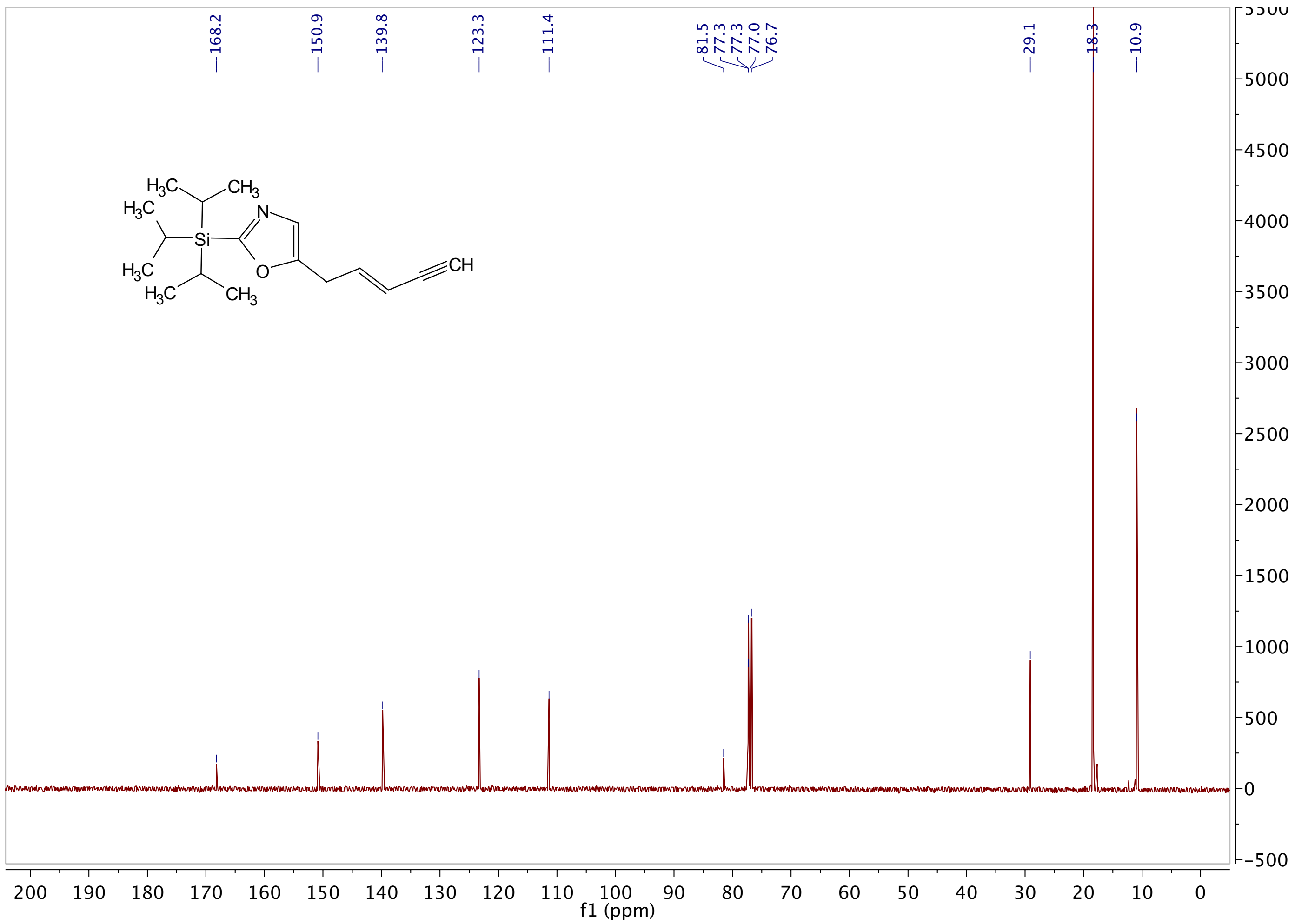

5-((2E,4E)-5-(4,4,5,5-Tetramethyl-1,3,2-dioxaborolan-2-yl)penta-2,4-dien-1-yl)-2 (triisopropylsilyl)oxazole (15)

^{1}H NMR (400 MHz, $CDCl_3$)

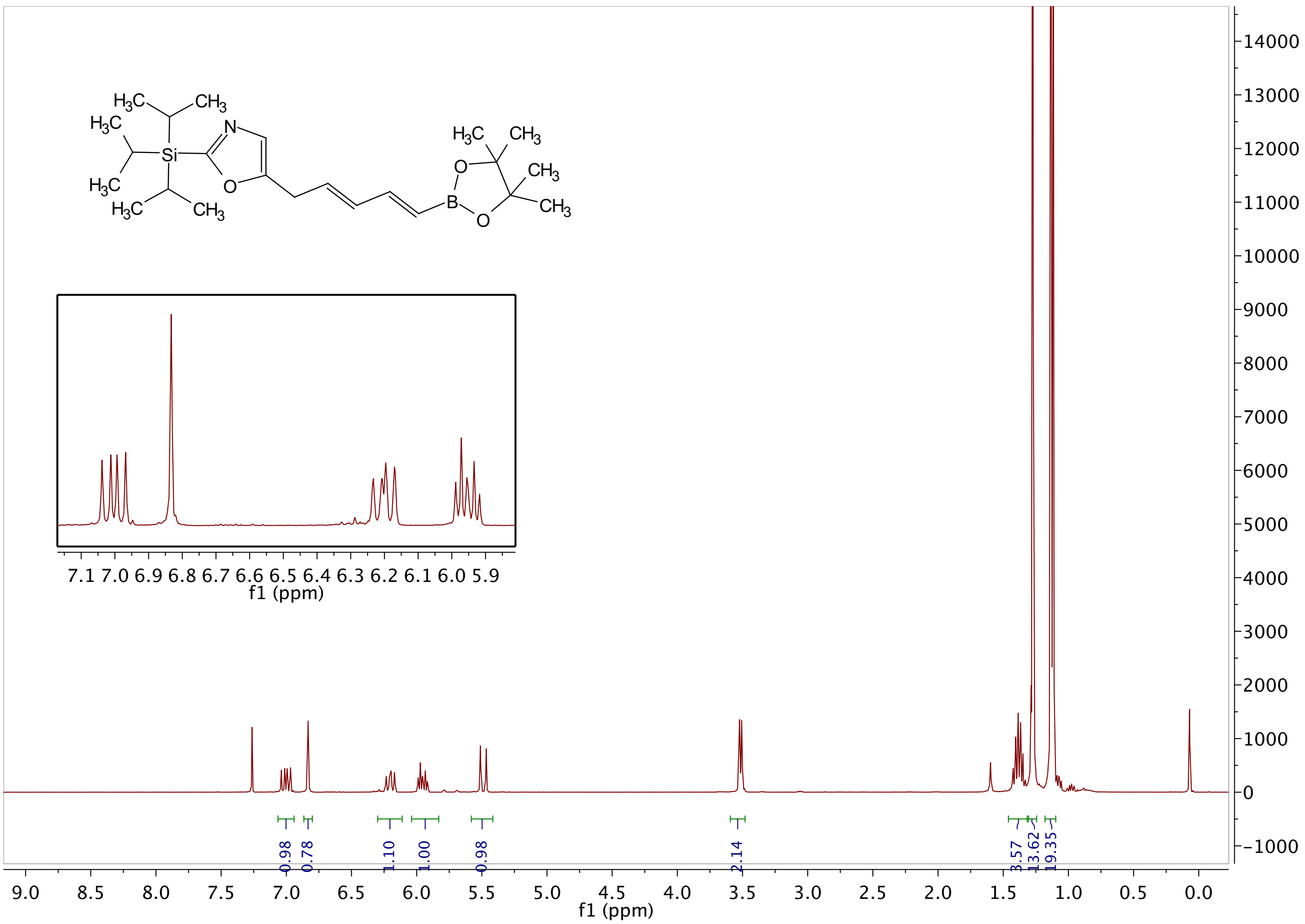

5-((2E,4E)-5-(4,4,5,5-Tetramethyl-1,3,2-dioxaborolan-2-yl)penta-2,4-dien-1-yl)-2 (triisopropylsilyl)oxazole (15)

^{13}C NMR (100 MHz, CDCl$_3$)

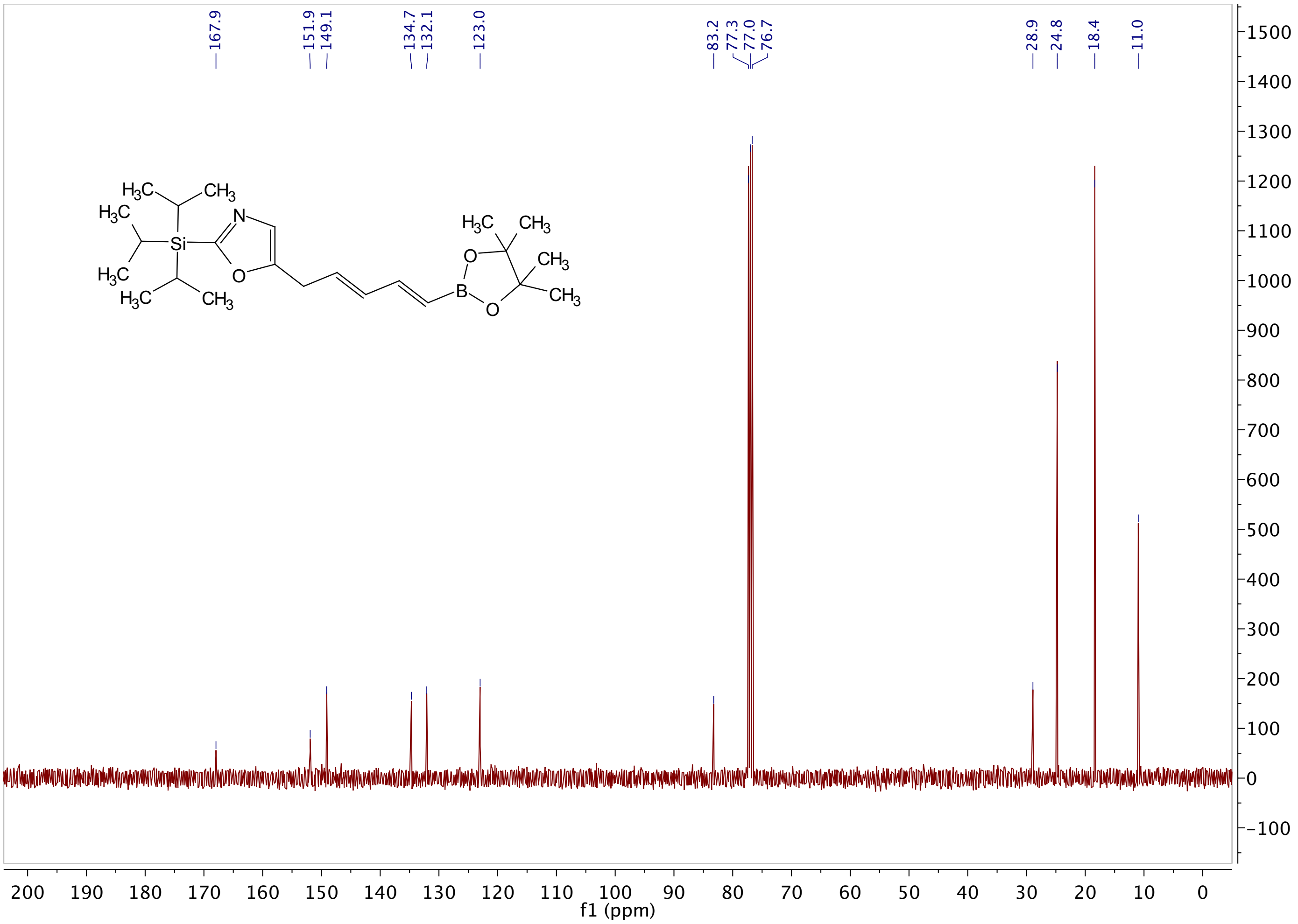

(*Z*)-3-Iodo-2-methylprop-2-en-1-ol ((*Z*)-16)

^{1}H NMR (400 MHz, CDCl$_3$)

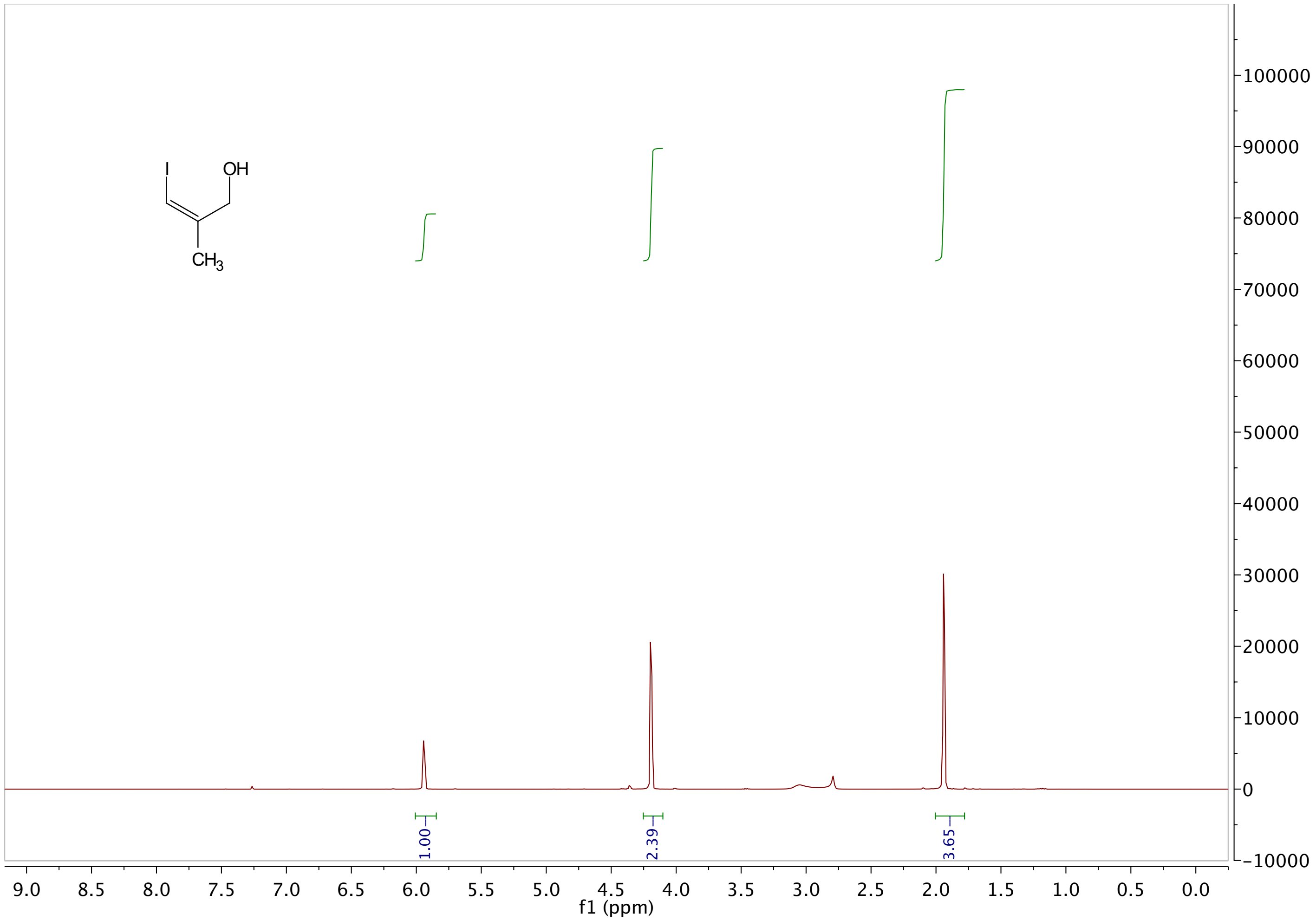

(*Z*)-3-Iodo-2-methylprop-2-en-1-ol ((*Z*)-16)

^{13}C NMR (100 MHz, CDCl3)

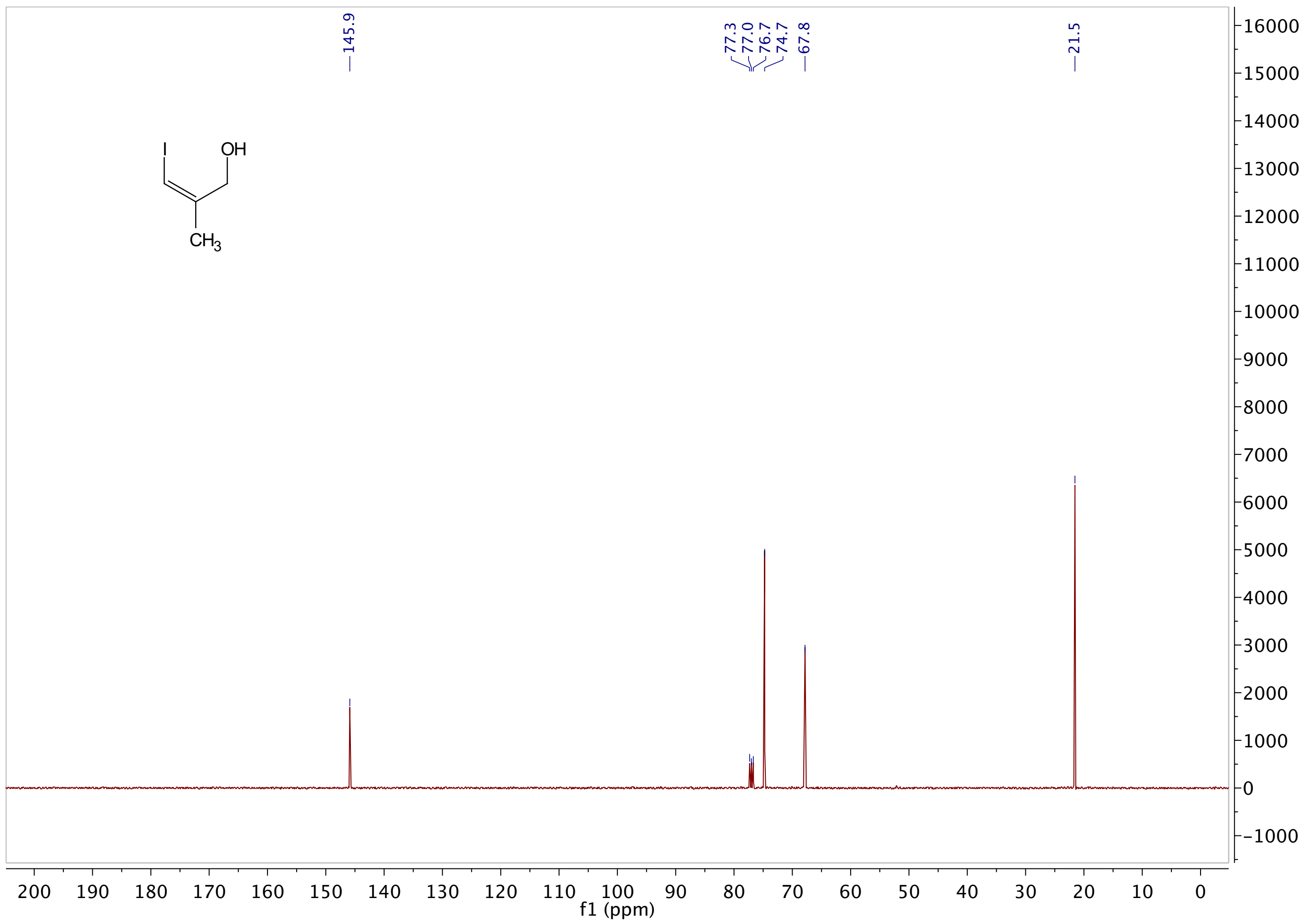

Methyl (S,Z)-3-hydroxy-5-iodo-2,2,4-trimethylpent-4-enoate ((Z)-5)

^{1}H NMR (400 MHz, $CDCl_3$)

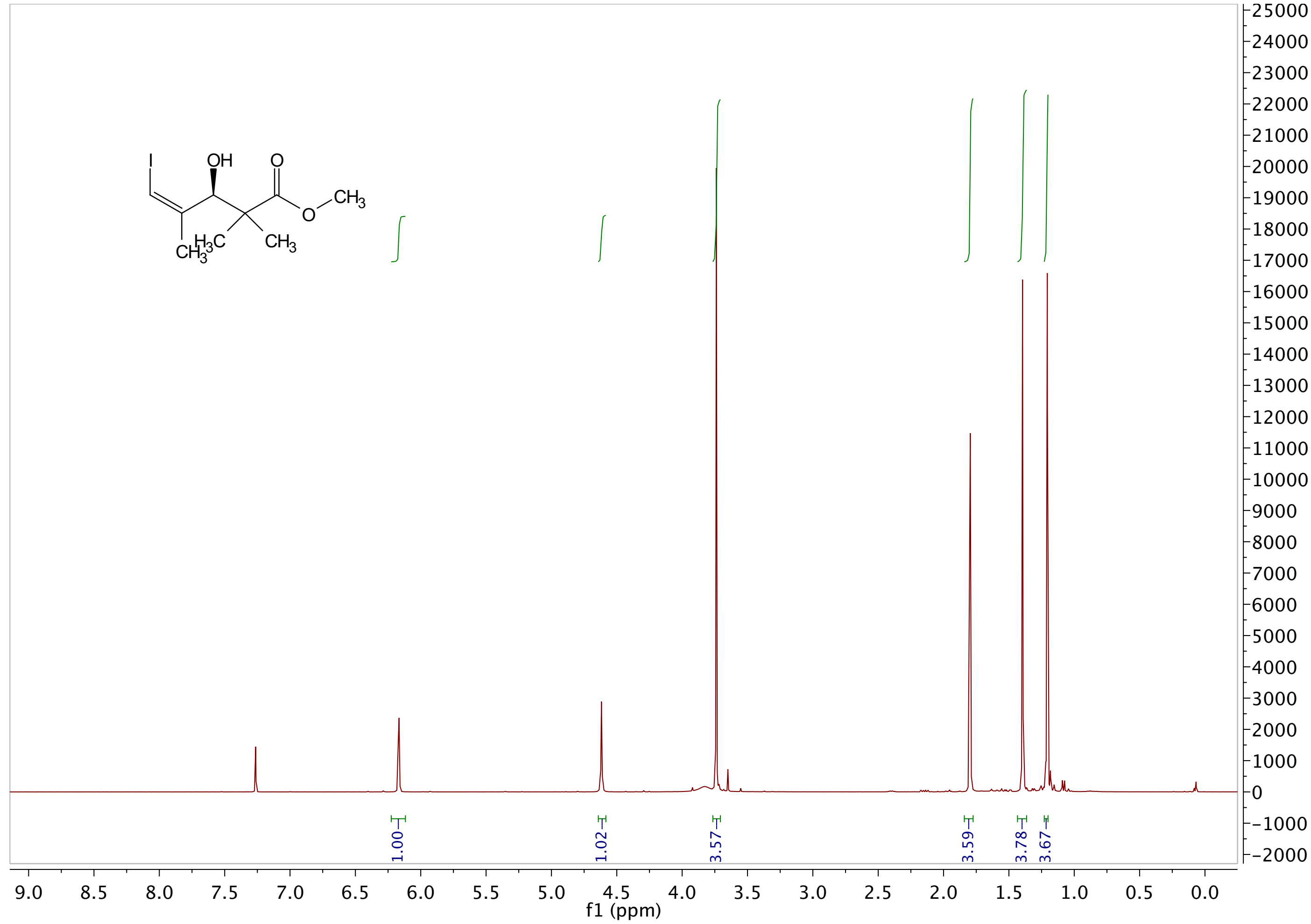

Methyl (S,Z)-3-hydroxy-5-iodo-2,2,4-trimethylpent-4-enoate ((Z)-5)

^{13}C NMR (100 MHz, CDCl$_3$)

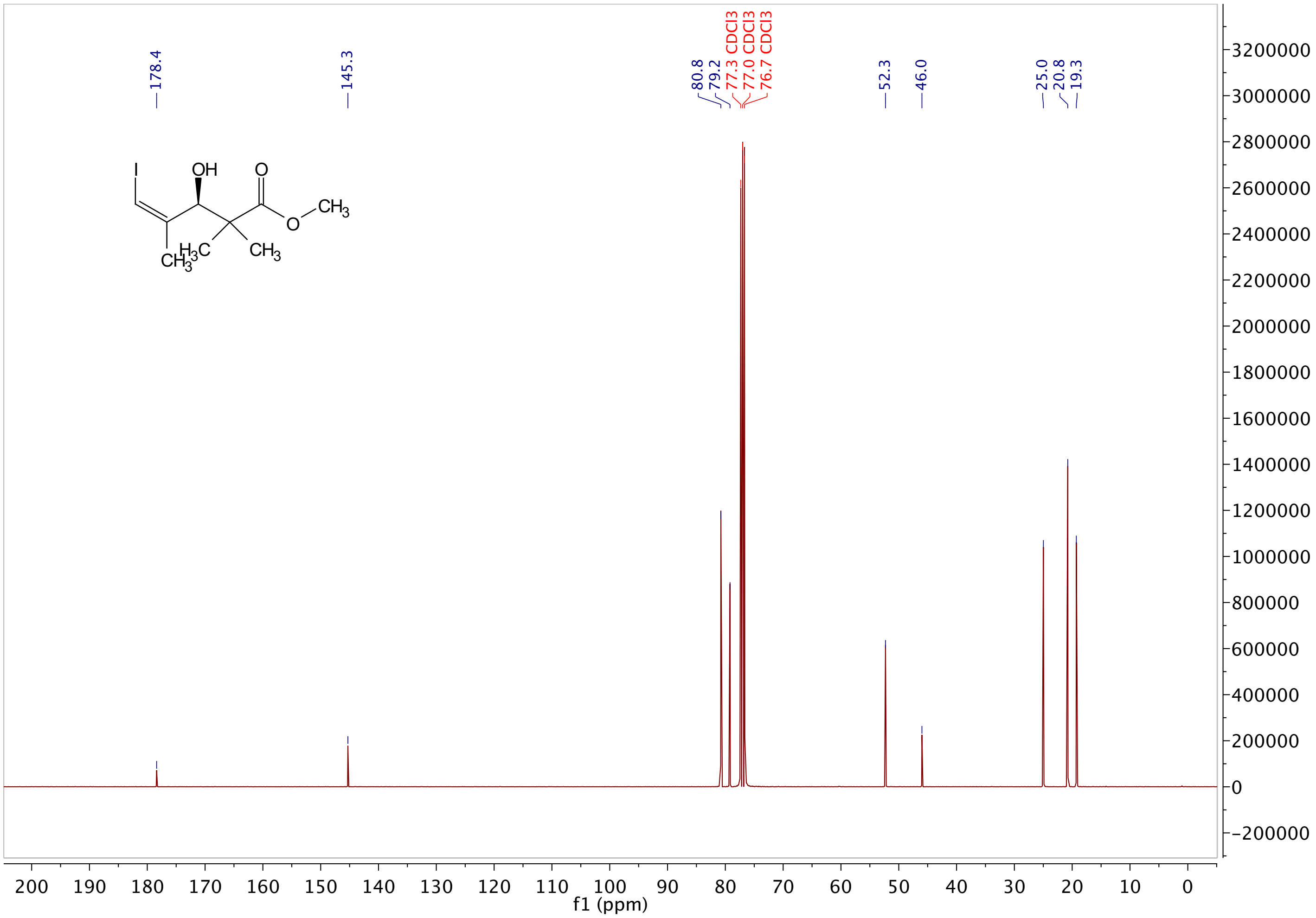

***Methyl* (S,Z)-5-iodo-2,2,4-trimethyl-3-((trimethylsilyl)oxy)pent-4-enoate ((Z)-17)**

^{1}H NMR (400 MHz, CDCl$_3$)

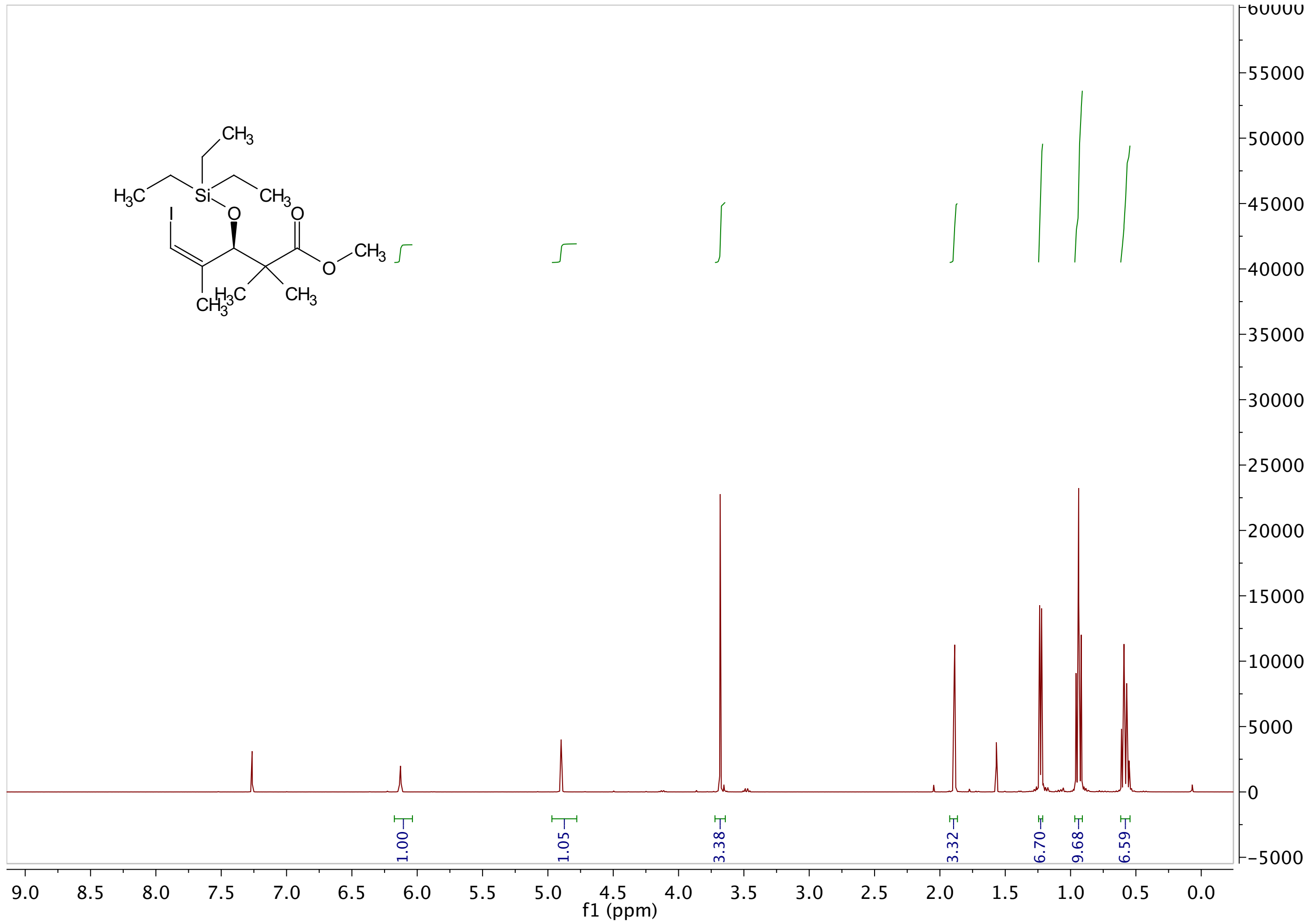

Methyl (S,Z)-5-iodo-2,2,4-trimethyl-3-((trimethylsilyl)oxy)pent-4-enoate ((Z)-17)

^{13}C NMR (100 MHz, CDCl$_3$)

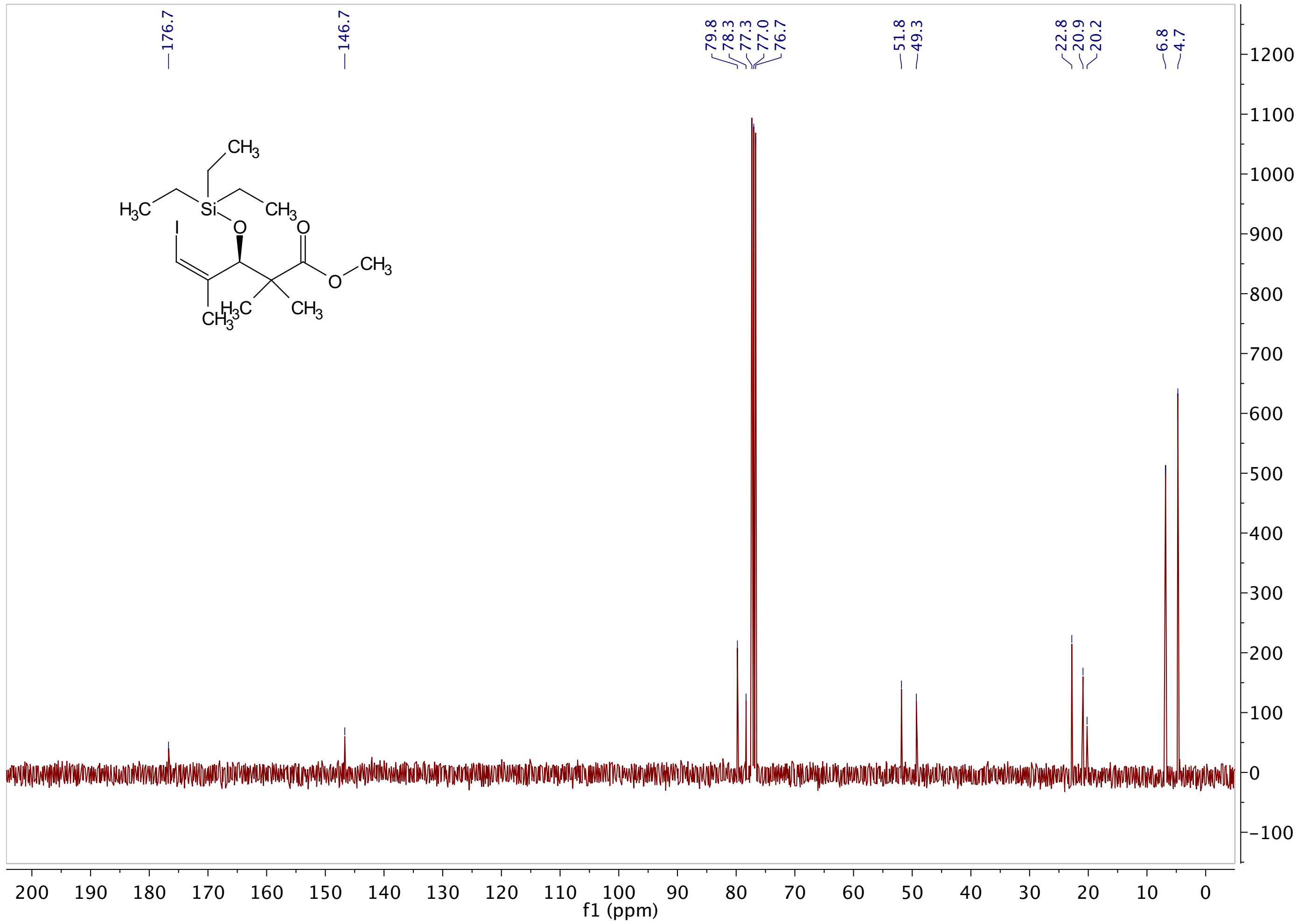

(*E*)-3-Iodo-2-methylprop-2-en-1-ol ((*E*)-16)

^{1}H NMR (400 MHz, $CDCl_3$)

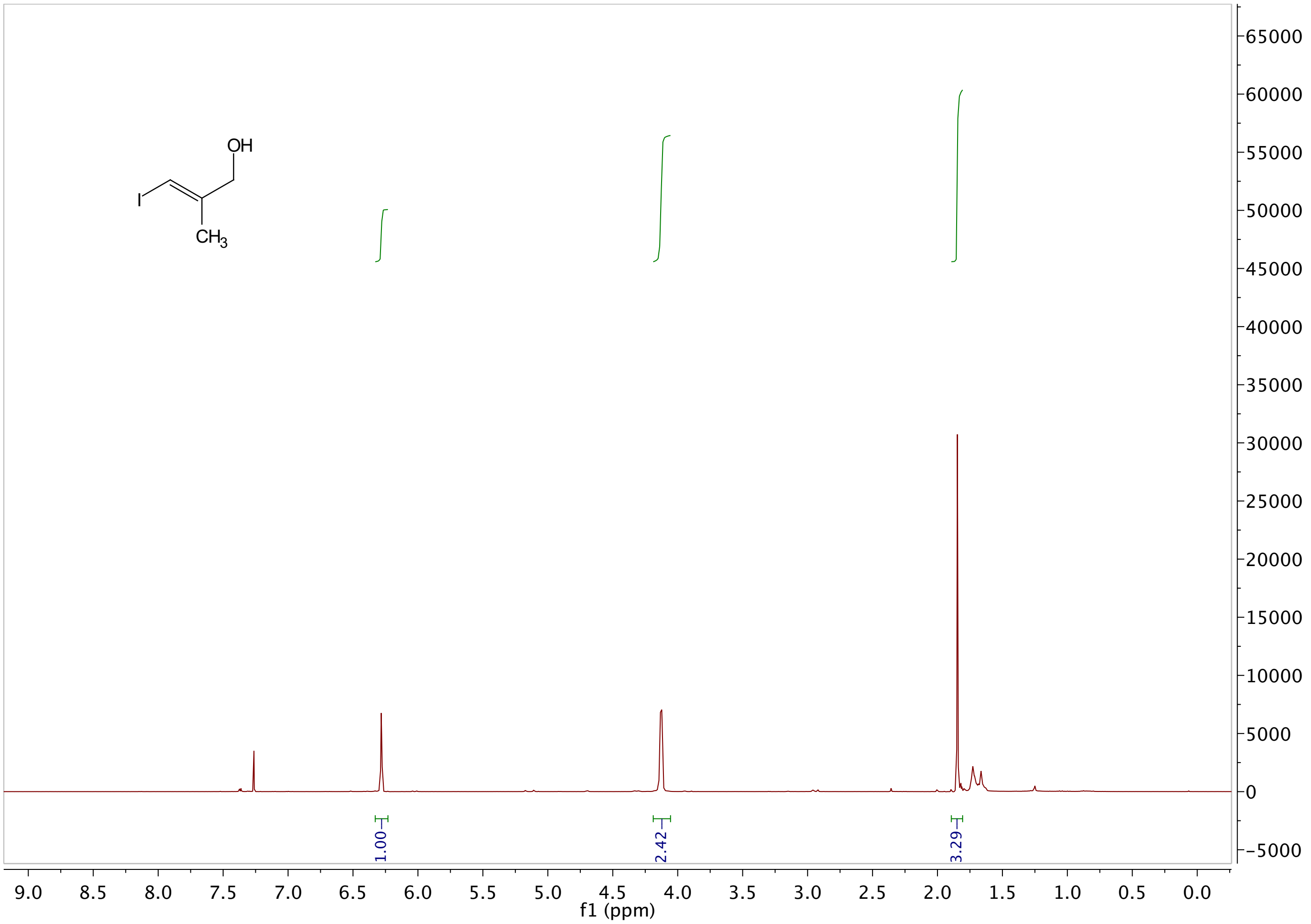

(*E*)-3-Iodo-2-methylprop-2-en-1-ol ((*E*)-16)

^{13}C NMR (100 MHz, $CDCl_3$)

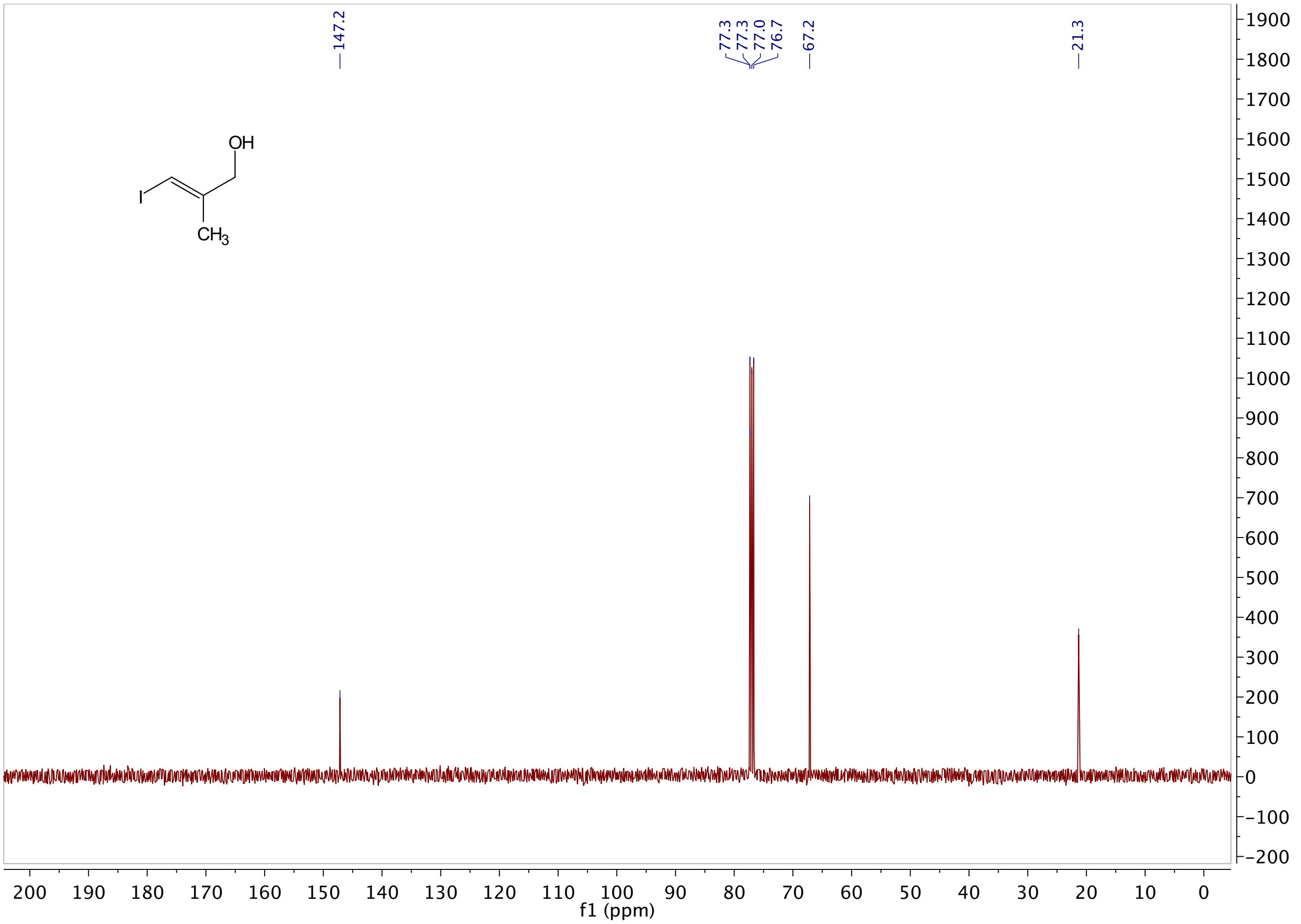

Methyl (S,E)-3-hydroxy-5-iodo-2,2,4-trimethylpent-4-enoate ((E)-5)

^{1}H NMR (400 MHz, $CDCl_3$)

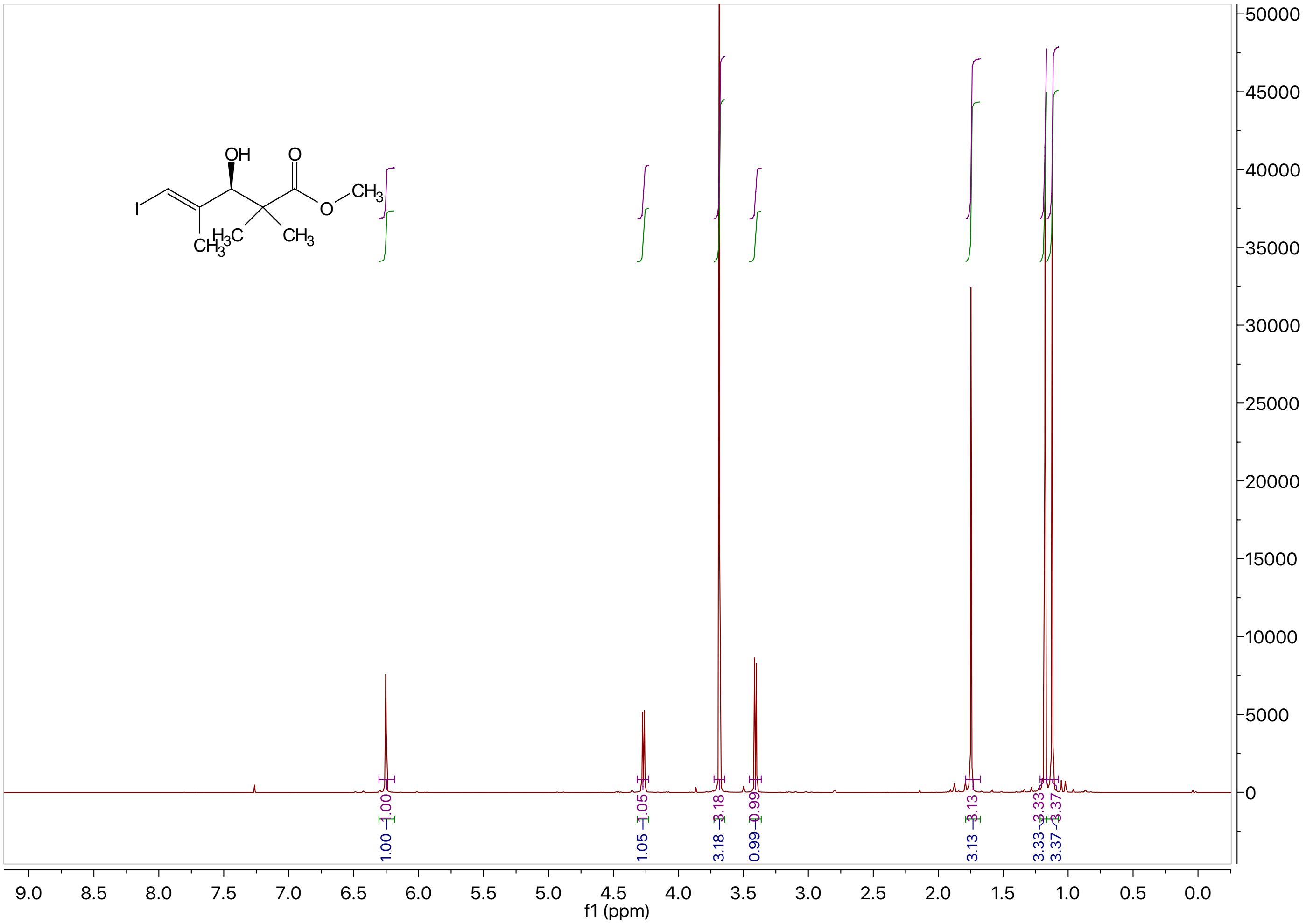

Methyl (S,E)-3-hydroxy-5-iodo-2,2,4-trimethylpent-4-enoate ((E)-5)

^{13}C NMR (100 MHz, $CDCl_3$)

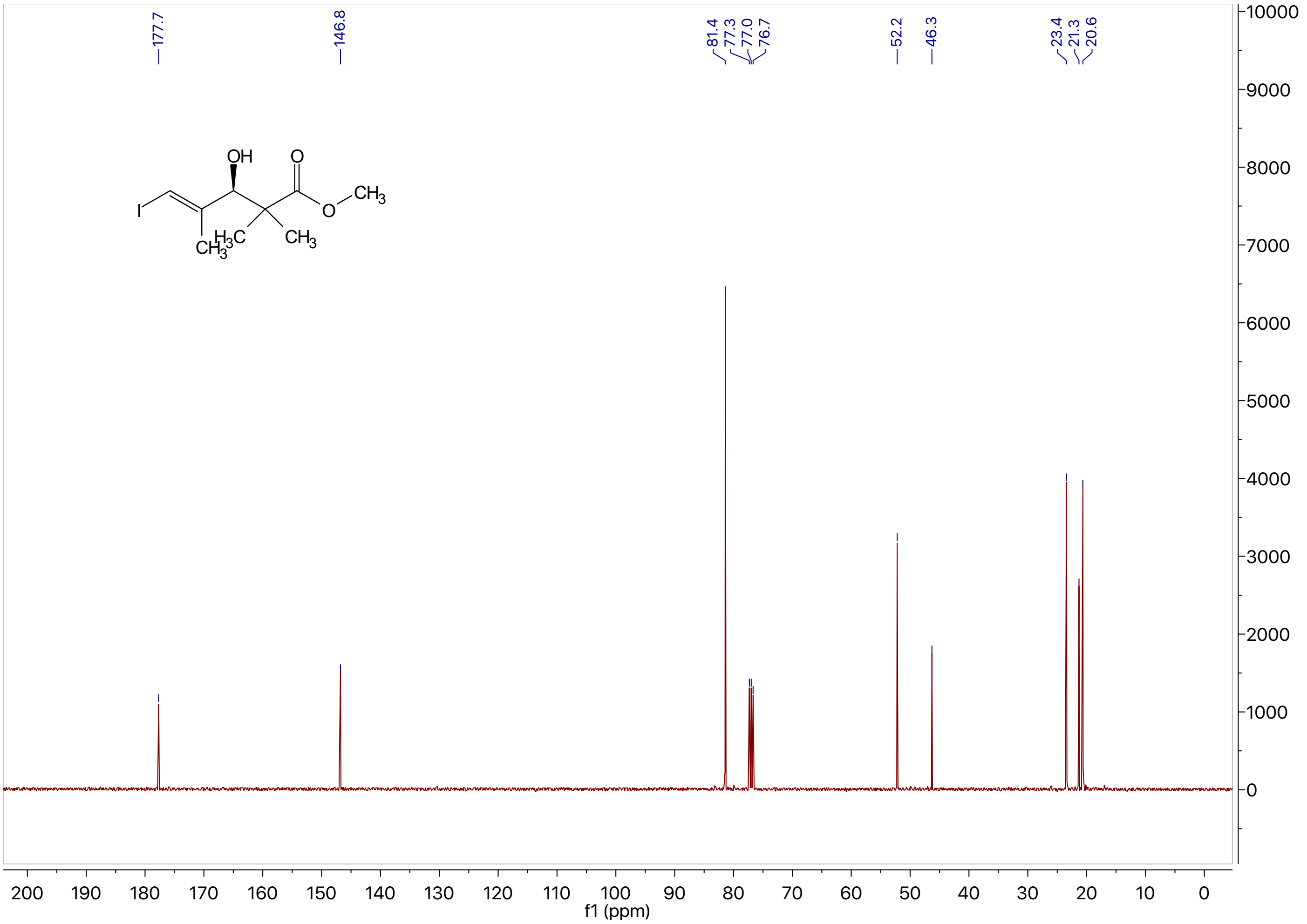

Methyl (S,E)-5-iodo-2,2,4-trimethyl-3-((trimethylsilyl)oxy)pent-4-enoate ((E)-17)

^{1}H NMR (400 MHz, CDCl$_3$)

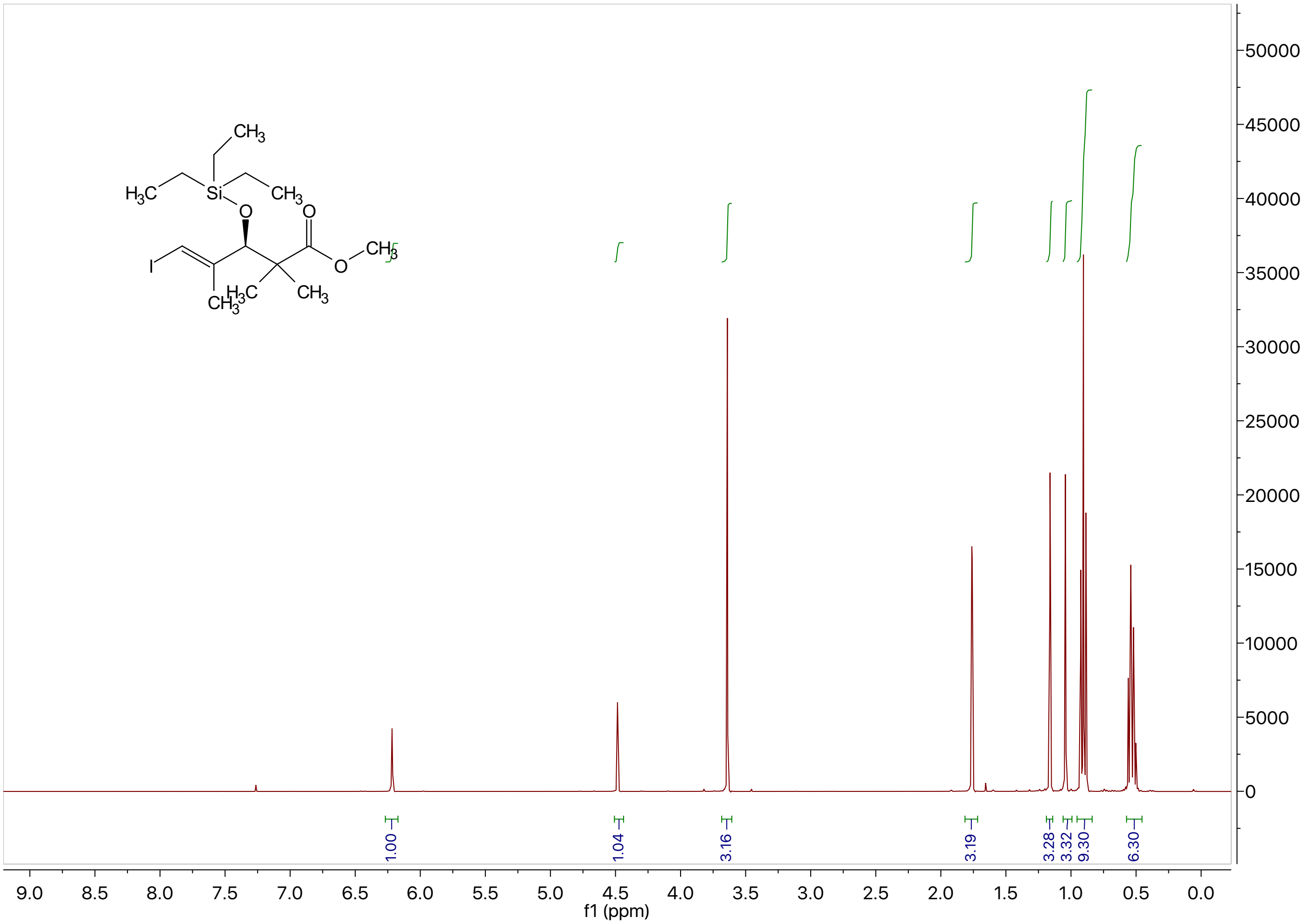

Methyl (S,E)-5-iodo-2,2,4-trimethyl-3-((trimethylsilyl)oxy)pent-4-enoate ((E)-17)

^{13}C NMR (100 MHz, $CDCl_3$)

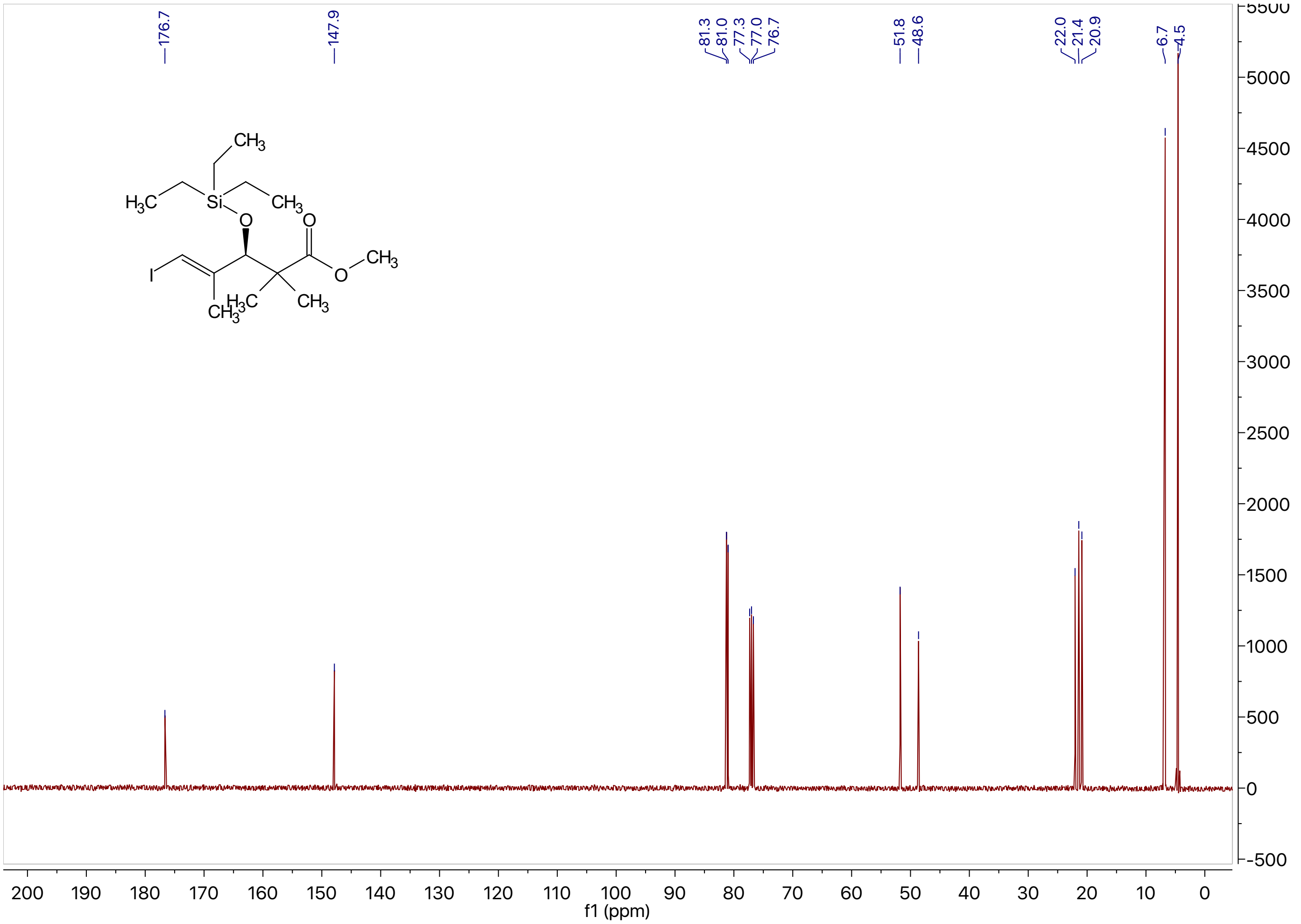

Methyl (R,4Z,6E,8E)-2,2,4-trimethyl-3-((triethylsilyl)oxy)-10-(2-(triisopropylsilyl)oxazol-5-yl)deca-4,6,8-trienoate (18)

^{1}H NMR (400 MHz, $CDCl_3$)

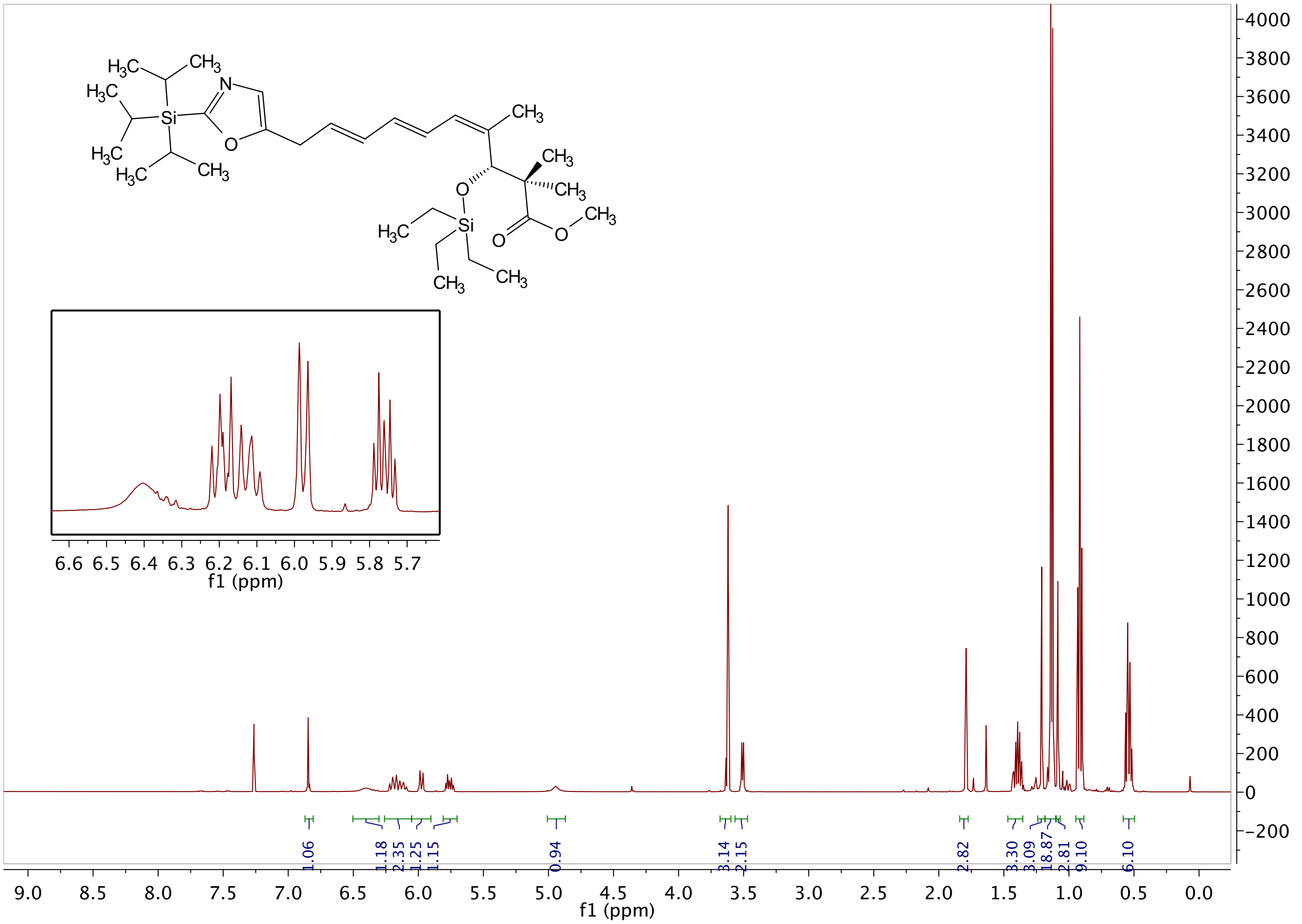

Methyl (R,4Z,6E,8E)-2,2,4-trimethyl-3-((triethylsilyl)oxy)-10-(2-(triisopropylsilyl)oxazol-5-yl)deca-4,6,8-trienoate (18)

^{13}C NMR (100 MHz, $CDCl_3$)

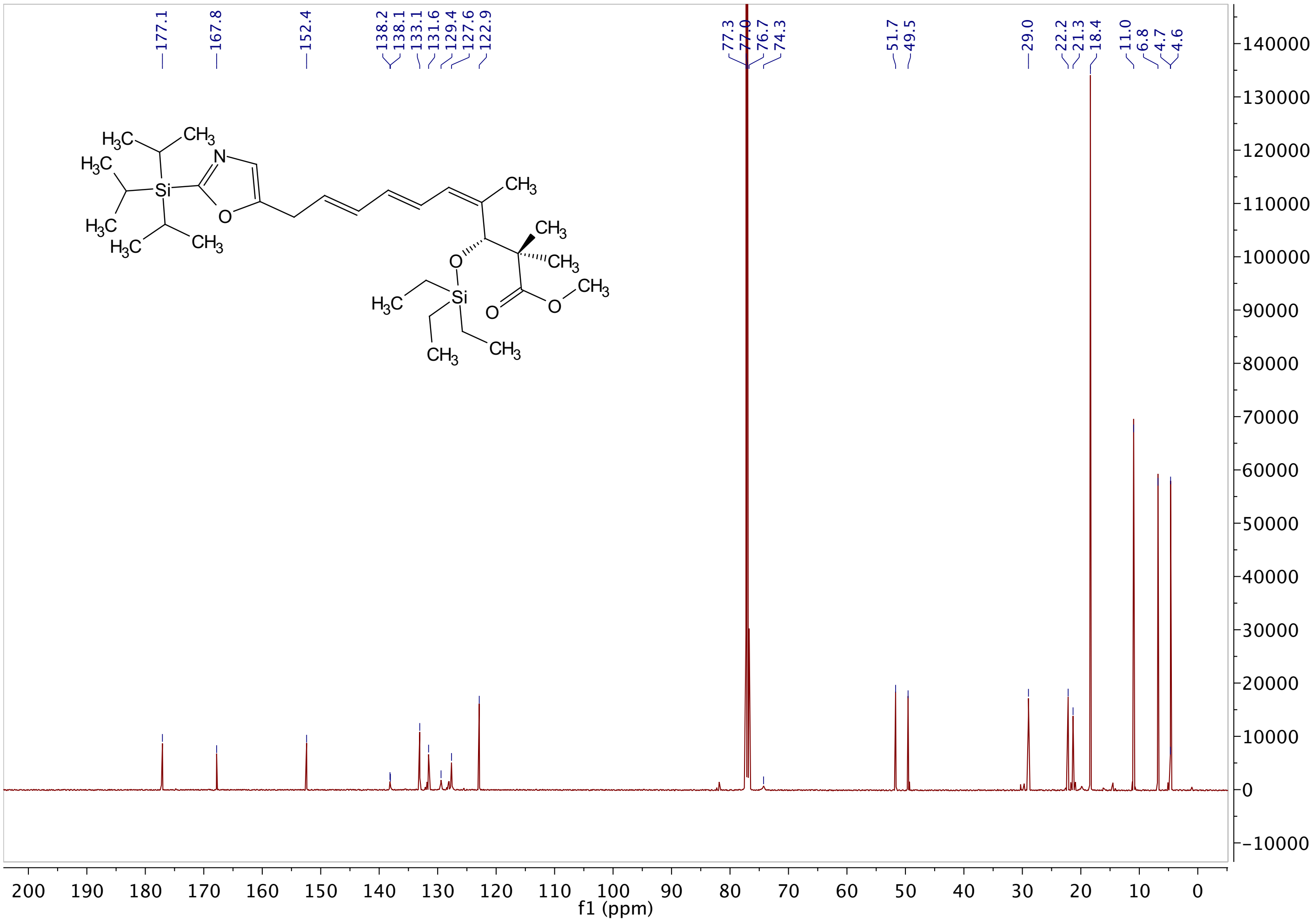

Methyl (*R*,4*Z*,6*E*,8*E*)-3-hydroxy-2,2,4-trimethyl-10-(oxazol-5-yl)deca-4,6,8-trienoate (19)

^{1}H NMR (400 MHz, $CDCl_3$)

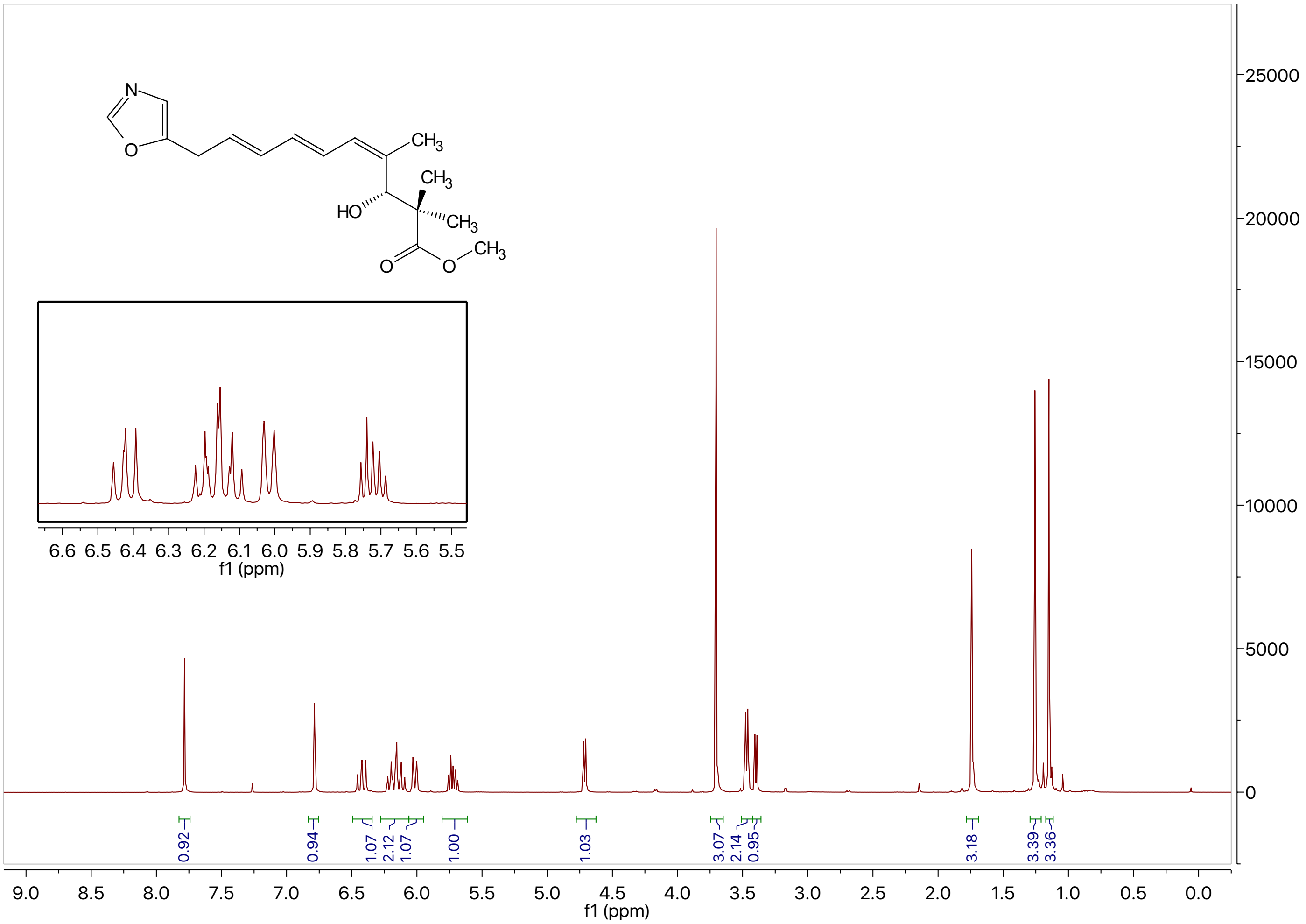

Methyl (*R*,4*Z*,6*E*,8*E*)-3-hydroxy-2,2,4-trimethyl-10-(oxazol-5-yl)deca-4,6,8-trienoate (19)

^{13}C NMR (100 MHz, $CDCl_3$)

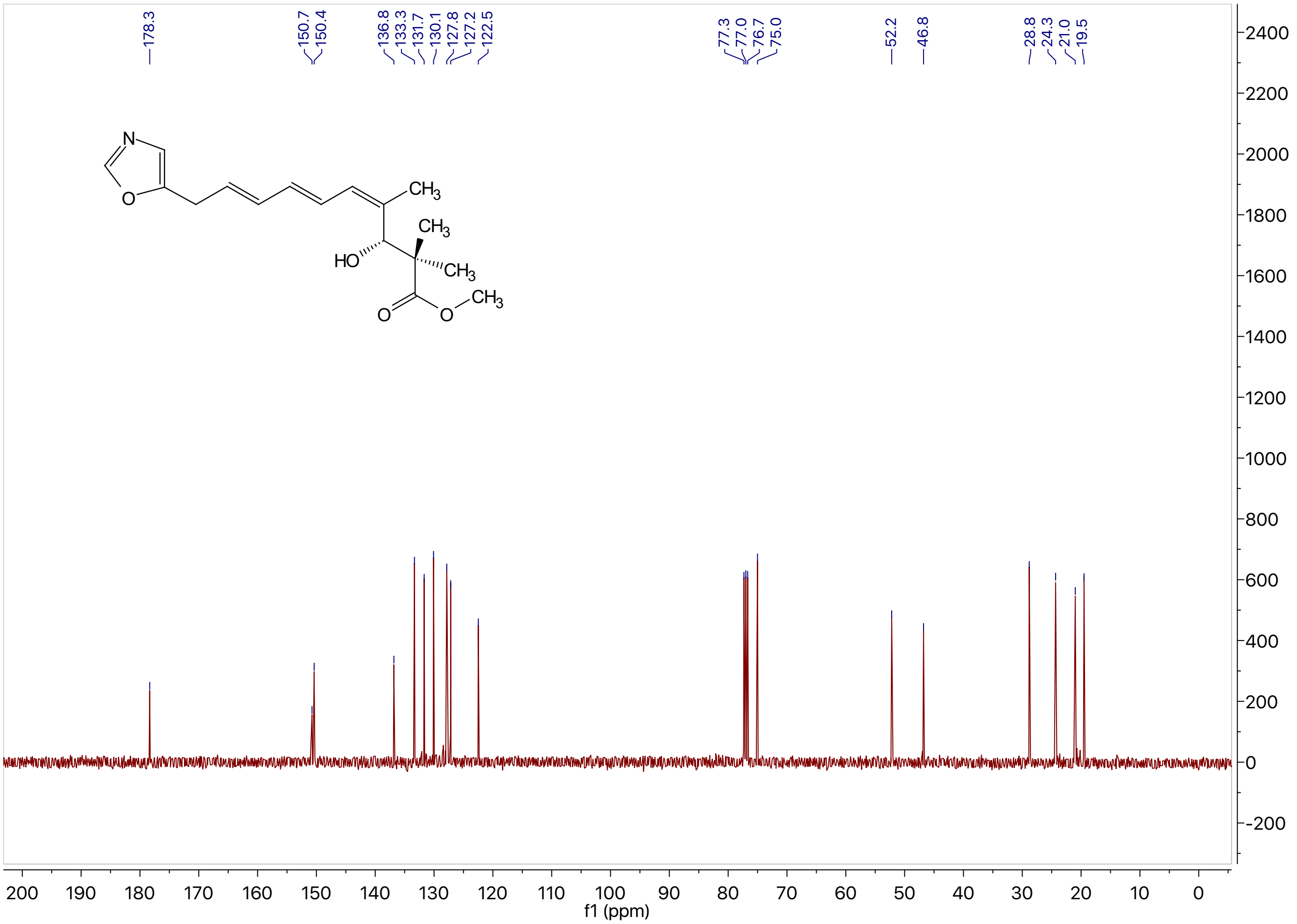

Perfluorophenyl (R,4Z,6E,8E)-3-hydroxy-2,2,4-trimethyl-10-(oxazol-5-yl)deca-4,6,8-trienoate (20)

^{1}H NMR (400 MHz, $CDCl_3$)

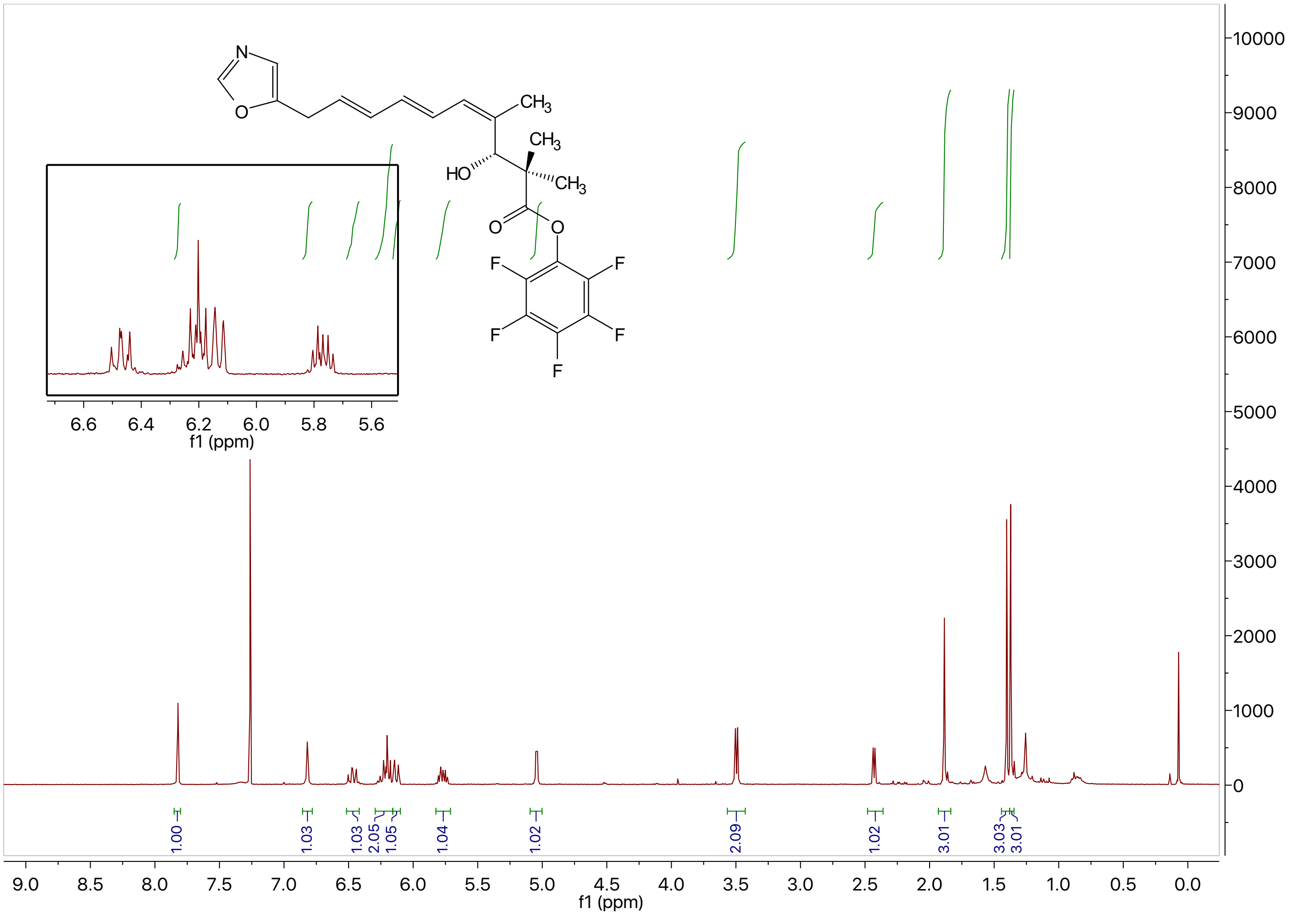

Perfluorophenyl (R,4Z,6E,8E)-3-hydroxy-2,2,4-trimethyl-10-(oxazol-5-yl)deca-4,6,8-trienoate (20):

^{13}C NMR (100 MHz, $CDCl_3$)

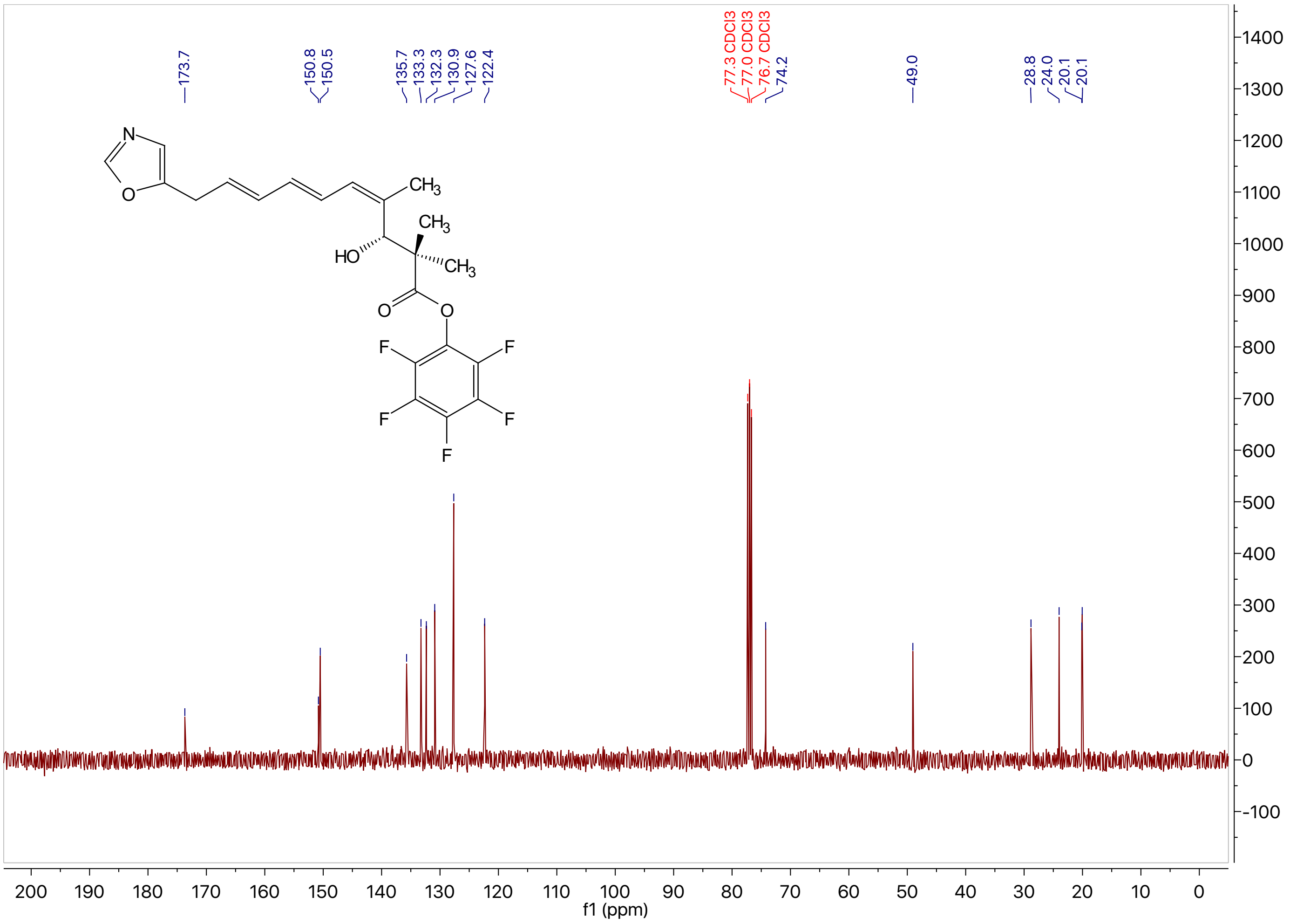

(*R*,4*Z*,6*E*,8*E*)-3-Hydroxy-2,2,4-trimethyl-10-(oxazol-5-yl)deca-4,6,8-trienamide (2)

^{1}H NMR (500 MHz, $CDCl_3$)

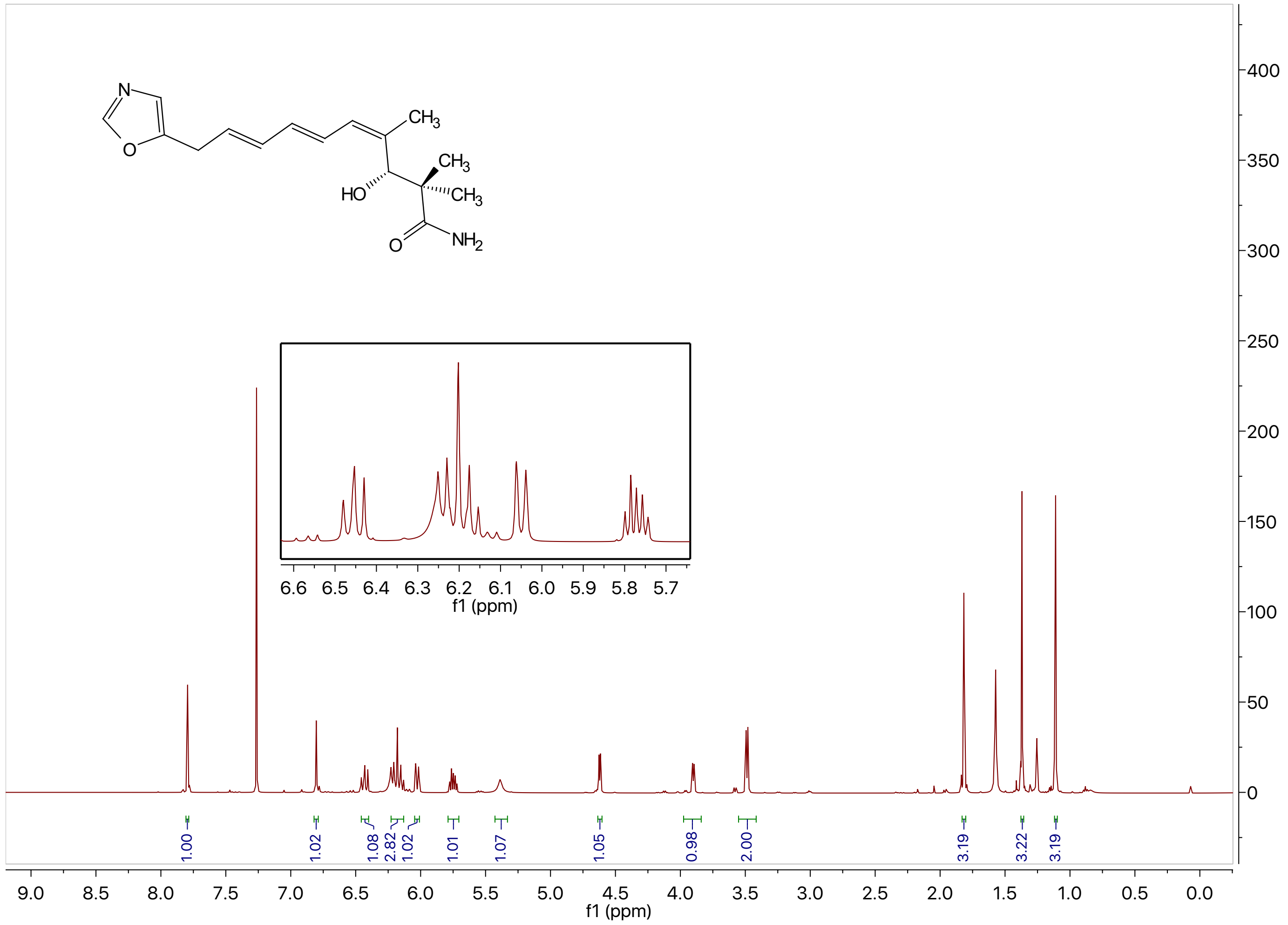

(*R*,4*Z*,6*E*,8*E*)-3-Hydroxy-2,2,4-trimethyl-10-(oxazol-5-yl)deca-4,6,8-trienamide (2)

^{13}C NMR (125 MHz, $CDCl_3$)

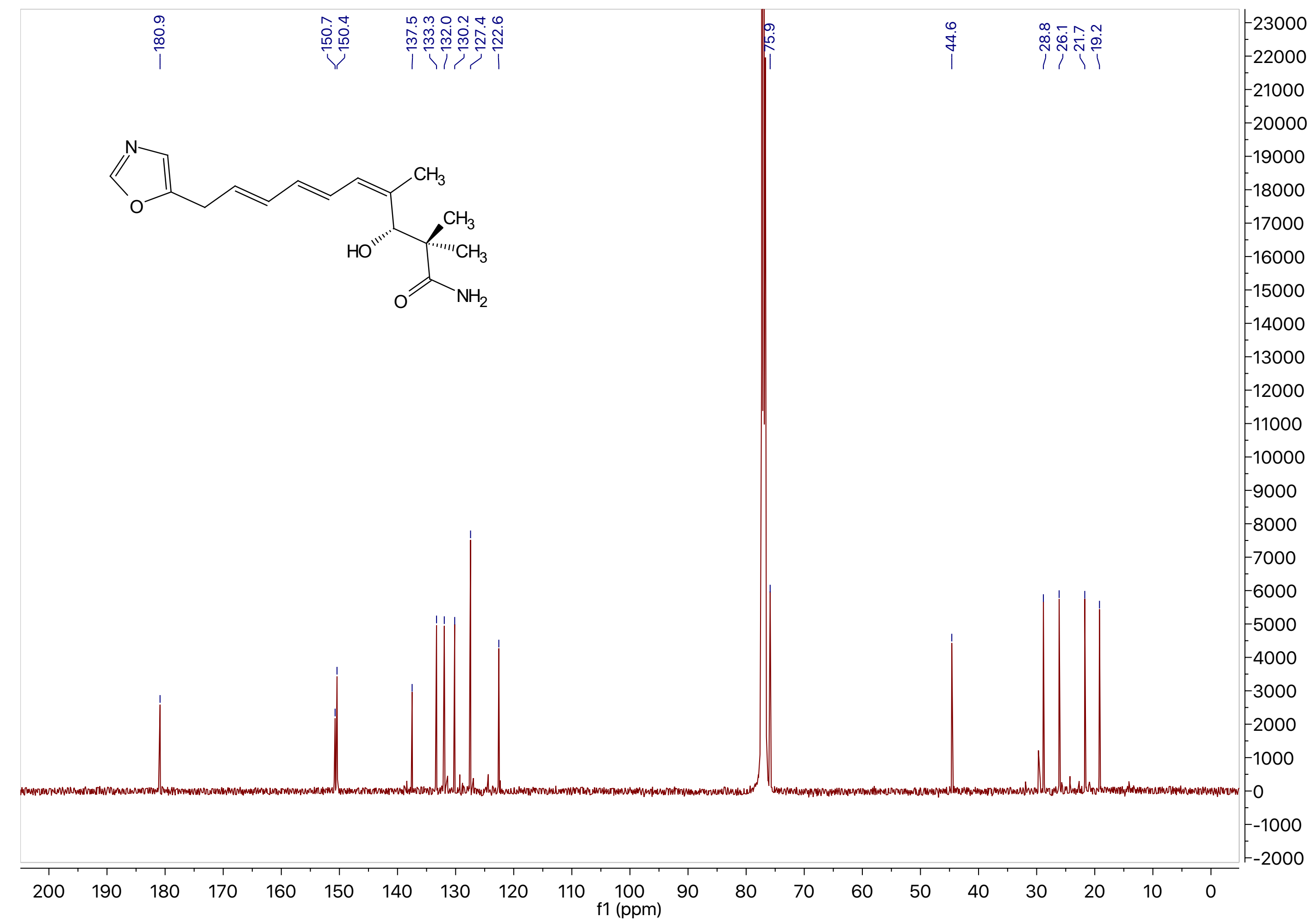

Methyl (*R*,4*E*,6*E*,8*E*)-2,2,4-trimethyl-3-((triethylsilyl)oxy)-10-(2-(triisopropylsilyl)oxazol-5-yl)

deca-4,6,8-trienoate (21)

^{1}H NMR (400 MHz, CDCl$_3$)

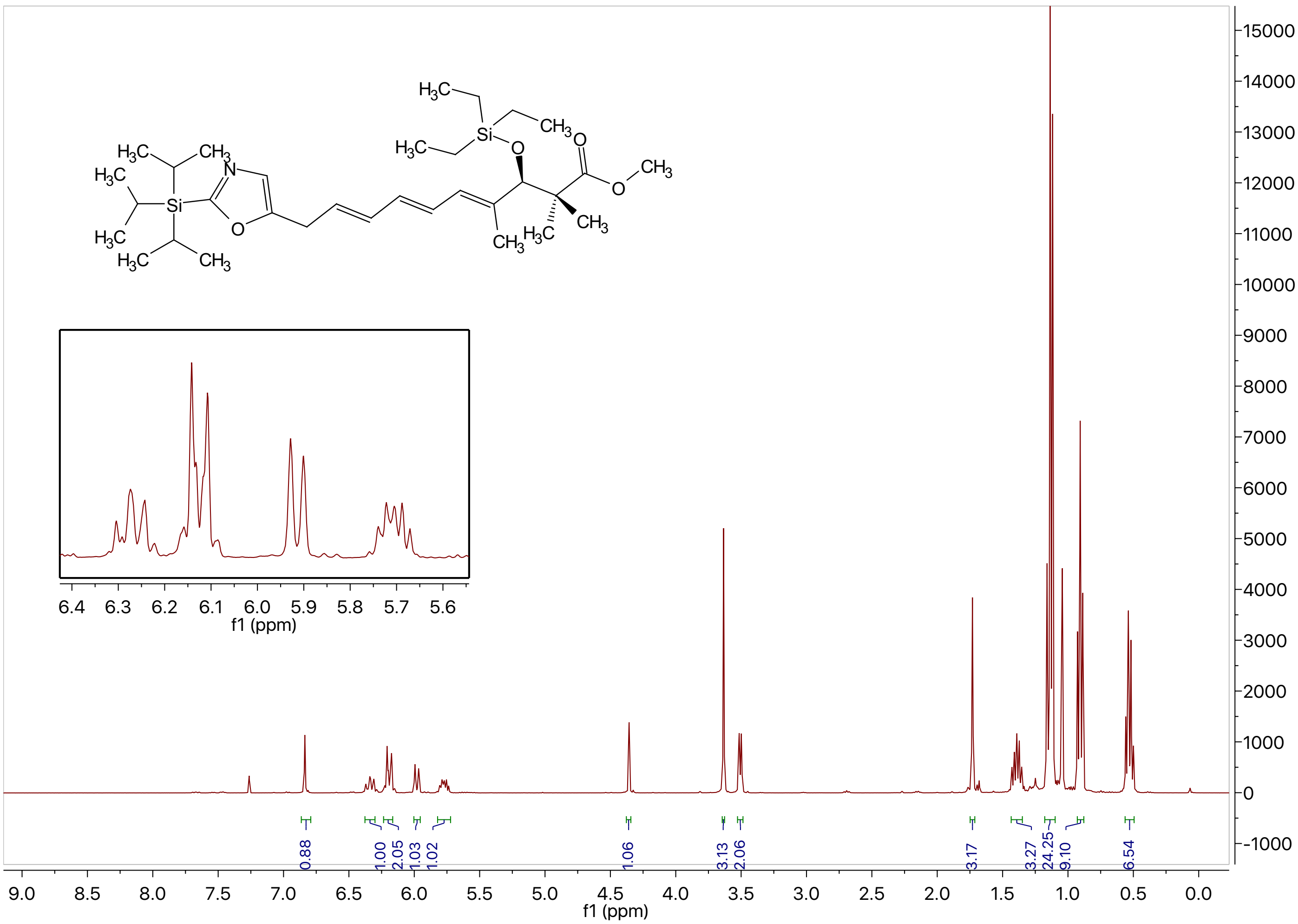

Methyl (R,4E,6E,8E)-2,2,4-trimethyl-3-((triethylsilyl)oxy)-10-(2-(triisopropylsilyl)

oxazol-5-yl)deca-4,6,8-trienoate (21)

^{13}C NMR (100 MHz, $CDCl_3$)

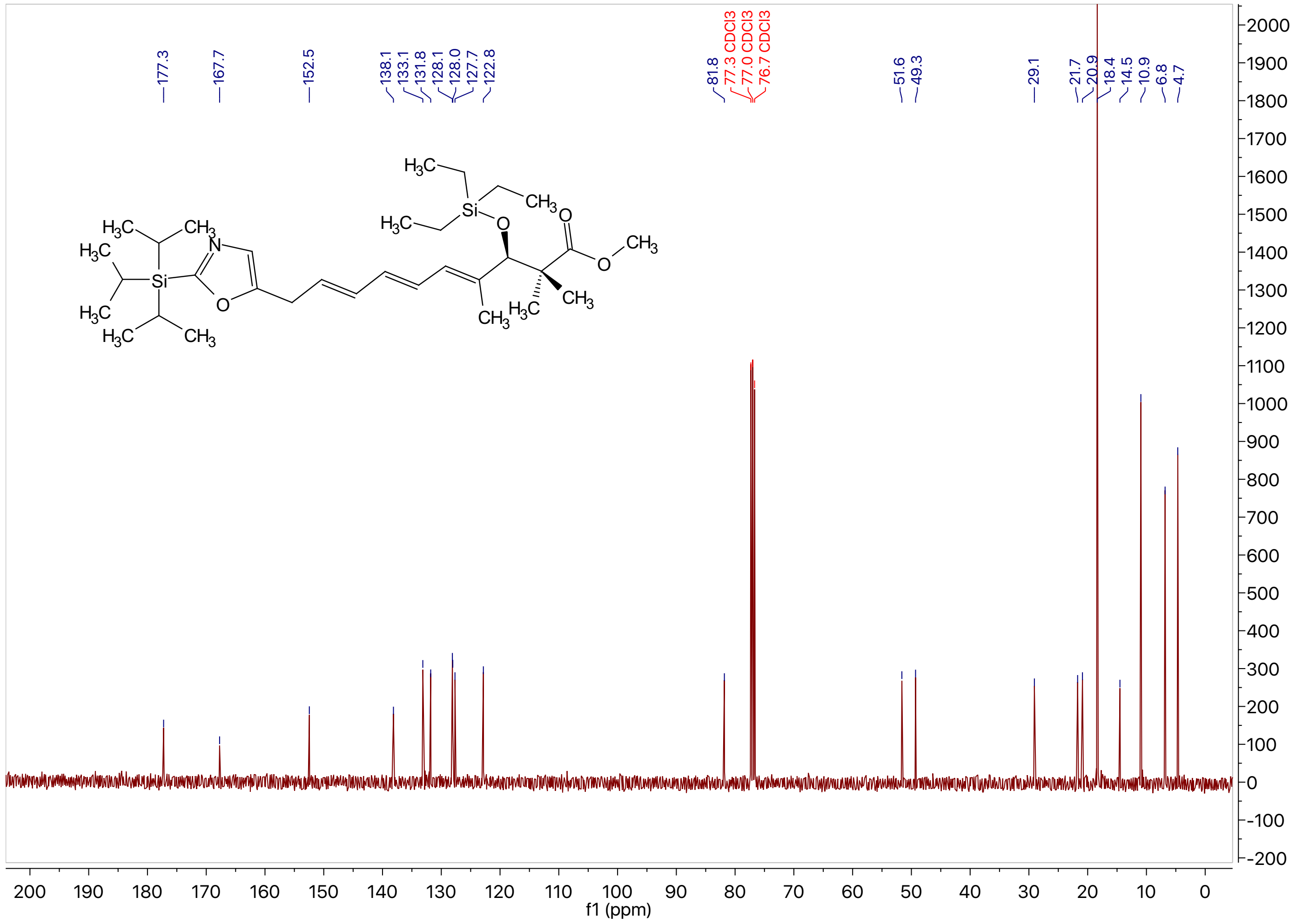

Methyl (*R*,4*E*,6*E*,8*E*)-3-hydroxy-2,2,4-trimethyl-10-(oxazol-5-yl)deca-4,6,8-trienoate (22)

^{1}H NMR (400 MHz, $CDCl_3$)

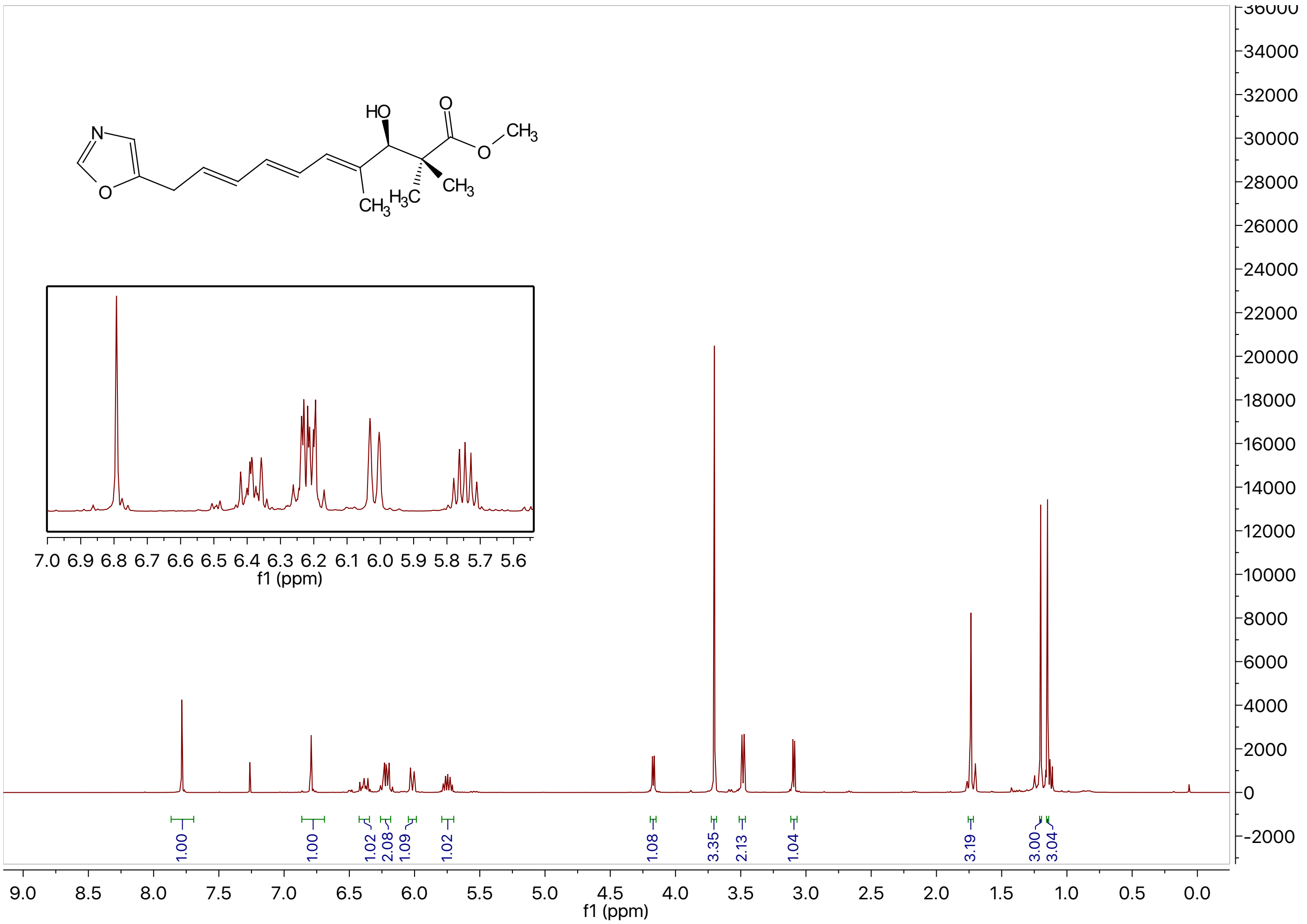

Methyl (*R*,4*E*,6*E*,8*E*)-3-hydroxy-2,2,4-trimethyl-10-(oxazol-5-yl)deca-4,6,8-trienoate (22)

^{13}C NMR (100 MHz, $CDCl_3$)

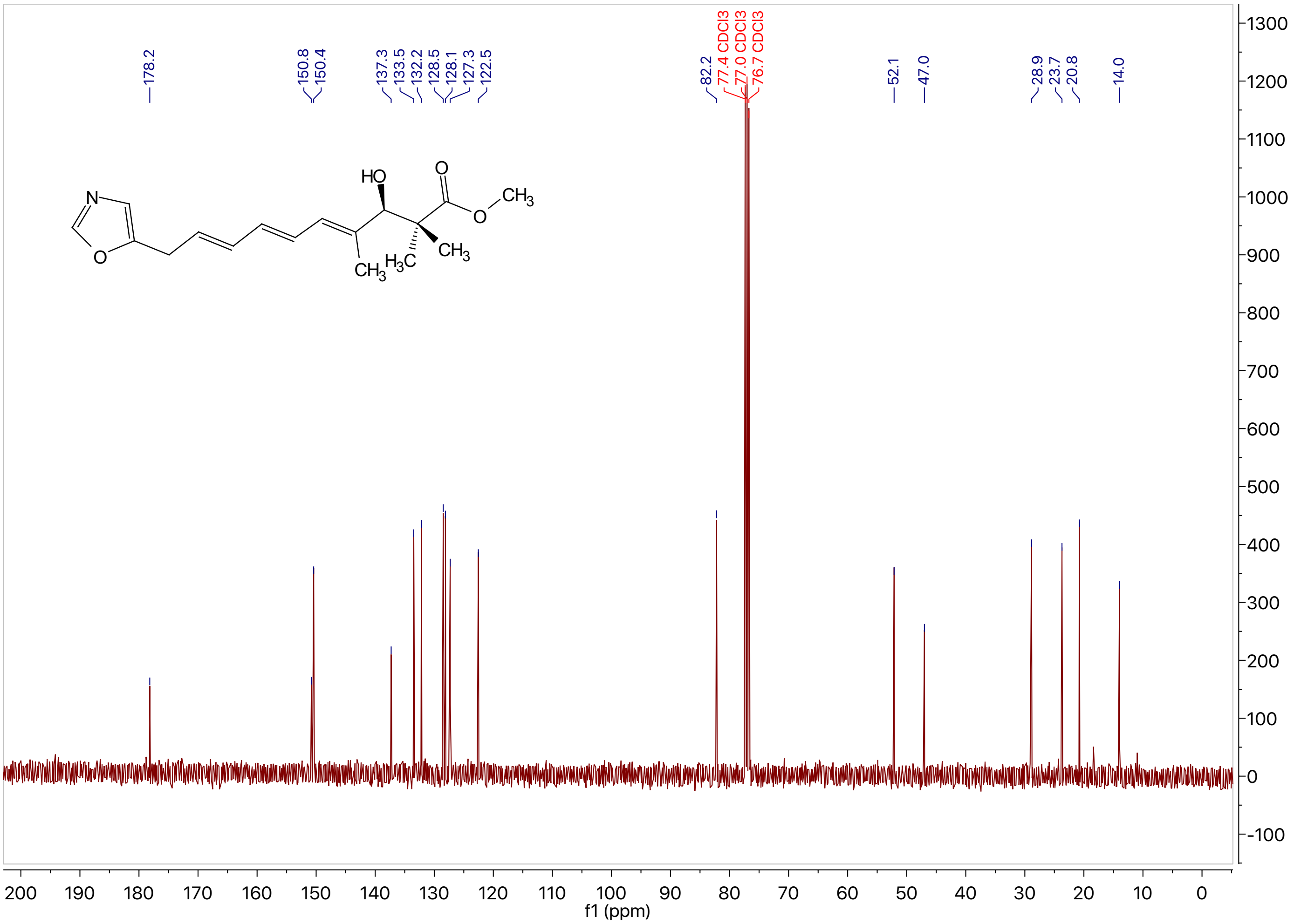

Perfluorophenyl (R,4E,6E,8E)-3-hydroxy-2,2,4-trimethyl-10-(oxazol-5-yl)deca-4,6,8-trienoate (23)

^{1}H NMR (400 MHz, CDCl$_3$)

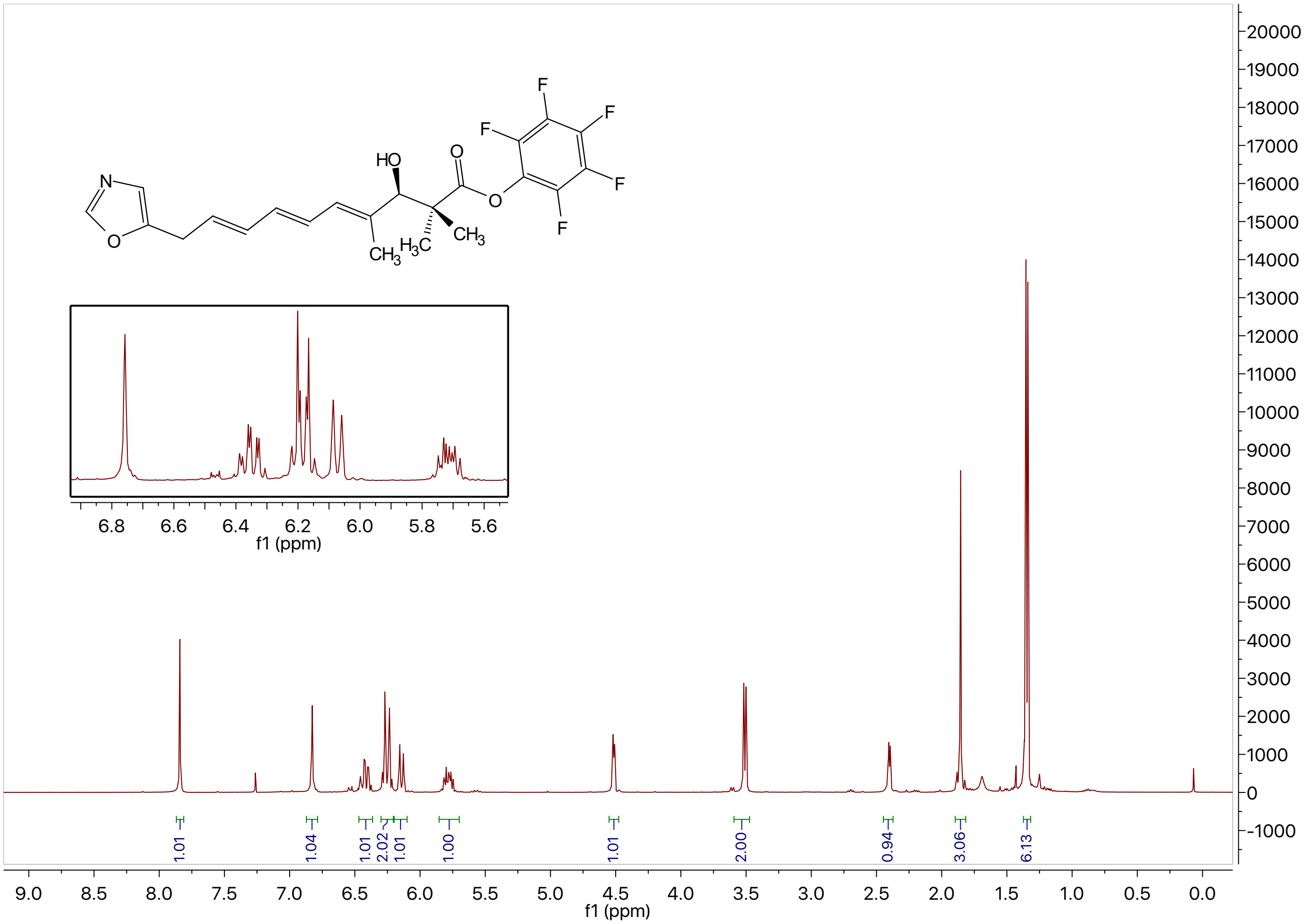

Perfluorophenyl (R,4E,6E,8E)-3-hydroxy-2,2,4-trimethyl-10-(oxazol-5-yl)deca-4,6,8-trienoate (23)

^{13}C NMR (100 MHz, $CDCl_3$)

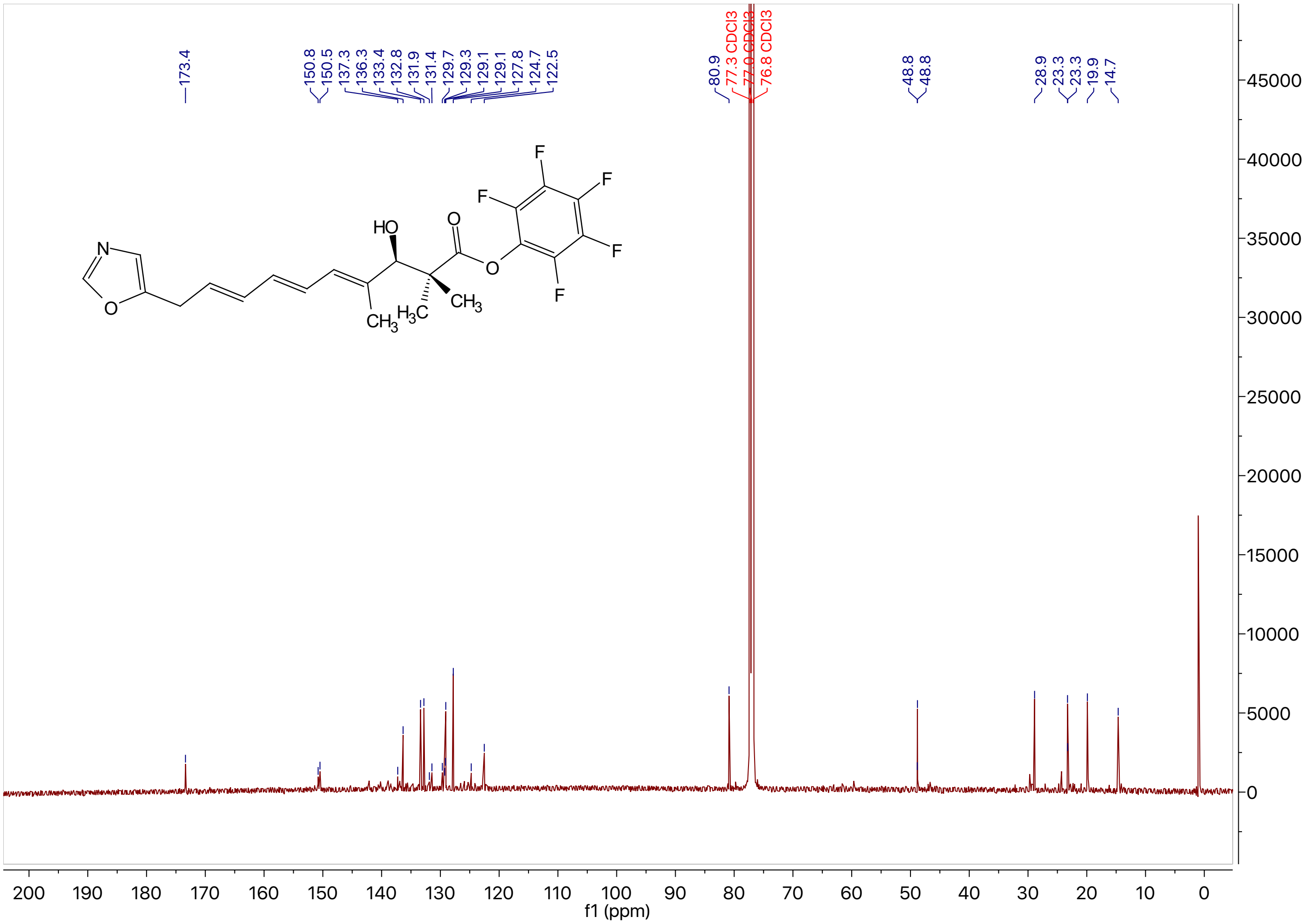

(R,4E,6E,8E)-3-hydroxy-2,2,4-trimethyl-10-(oxazol-5-yl)deca-4,6,8-trienamide (3)

^{1}H NMR (400 MHz, $CDCl_3$)

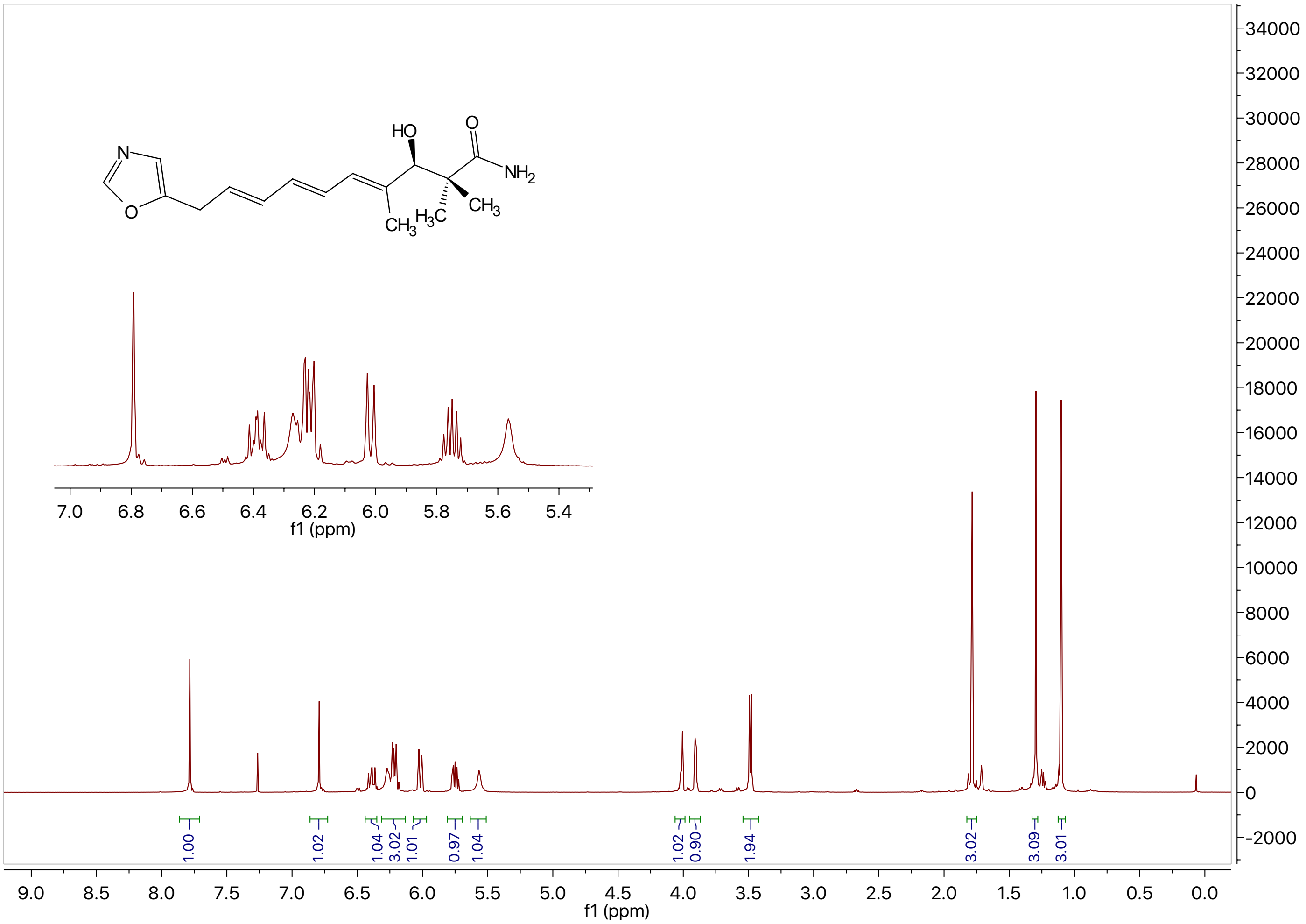

(R,4E,6E,8E)-3-hydroxy-2,2,4-trimethyl-10-(oxazol-5-yl)deca-4,6,8-trienamide (3)

^{13}C NMR (125 MHz, $CDCl_3$)

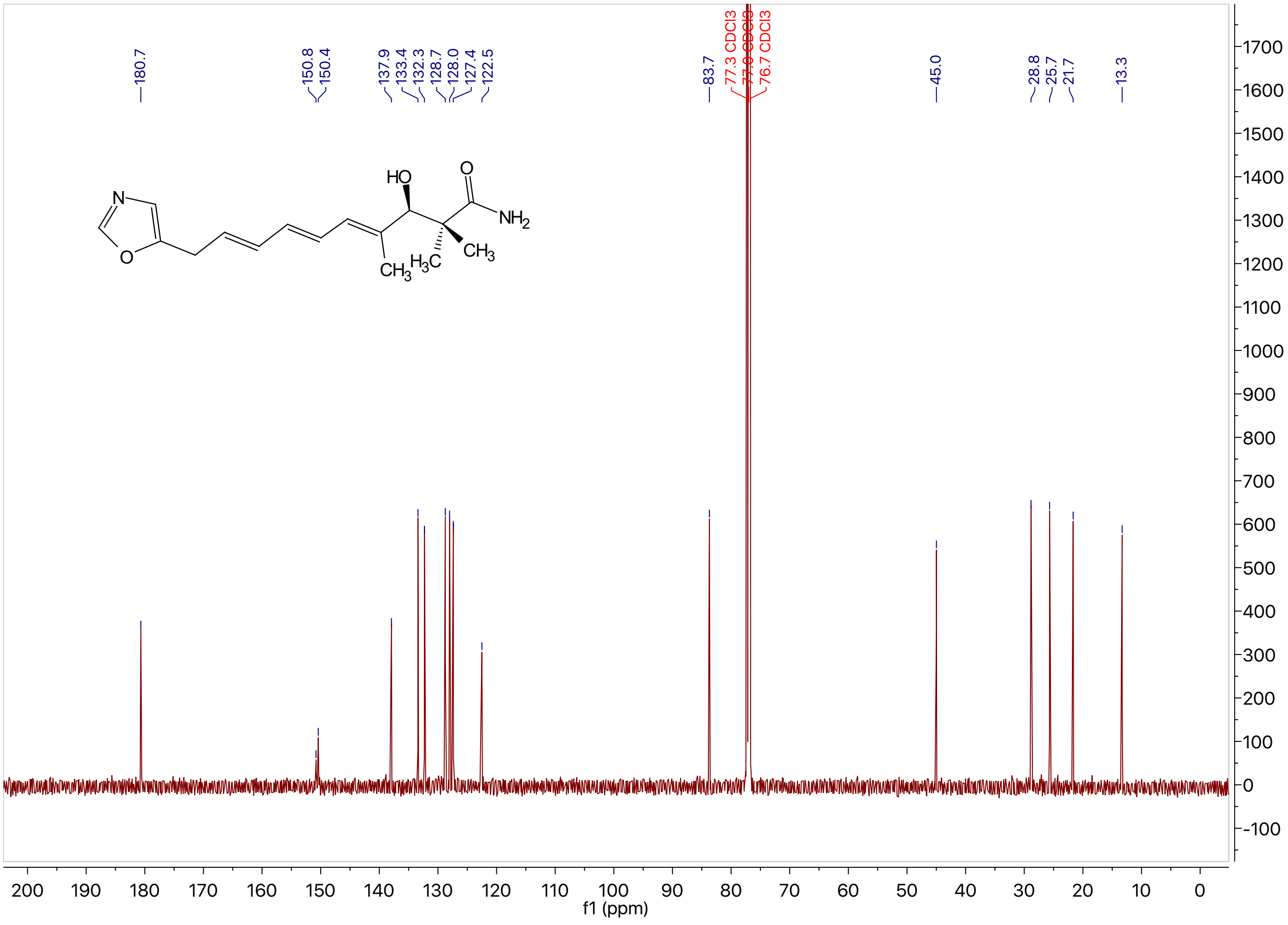

Methyl (*R*,4*Z*,8*E*)-2,2,4-trimethyl-3-((triethylsilyl)oxy)-10-(2-(triisopropylsilyl)oxazol-5-yl)deca-4,8-dien-6-ynoate (24):

^{1}H NMR (400 MHz, $CDCl_3$)

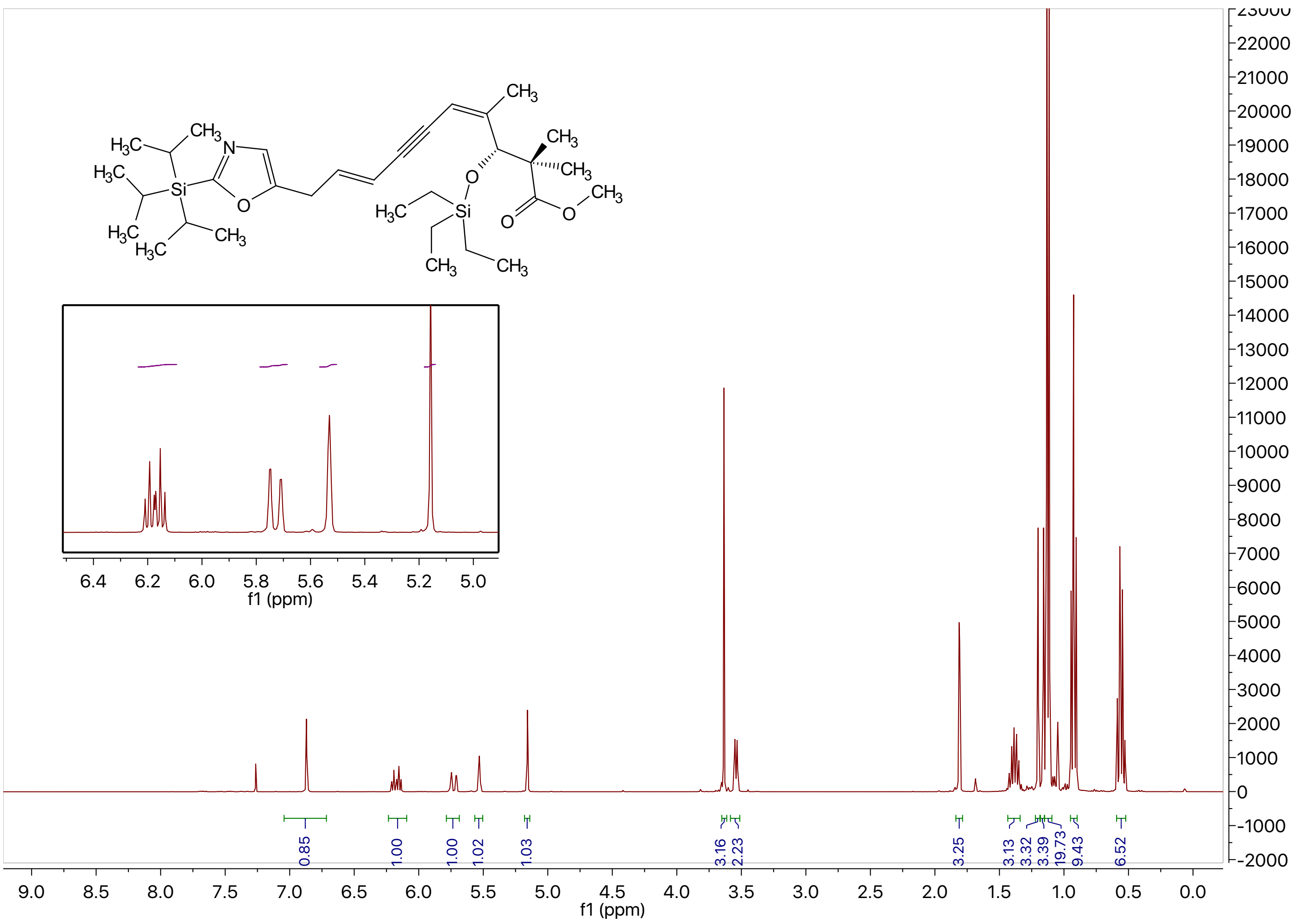

Methyl (*R*,4*Z*,8*E*)-2,2,4-trimethyl-3-((triethylsilyl)oxy)-10-(2-(triisopropylsilyl)oxazol-5-yl)deca-4,8-dien-6-ynoate (24):

^{13}C NMR (100 MHz, CDCl$_3$)

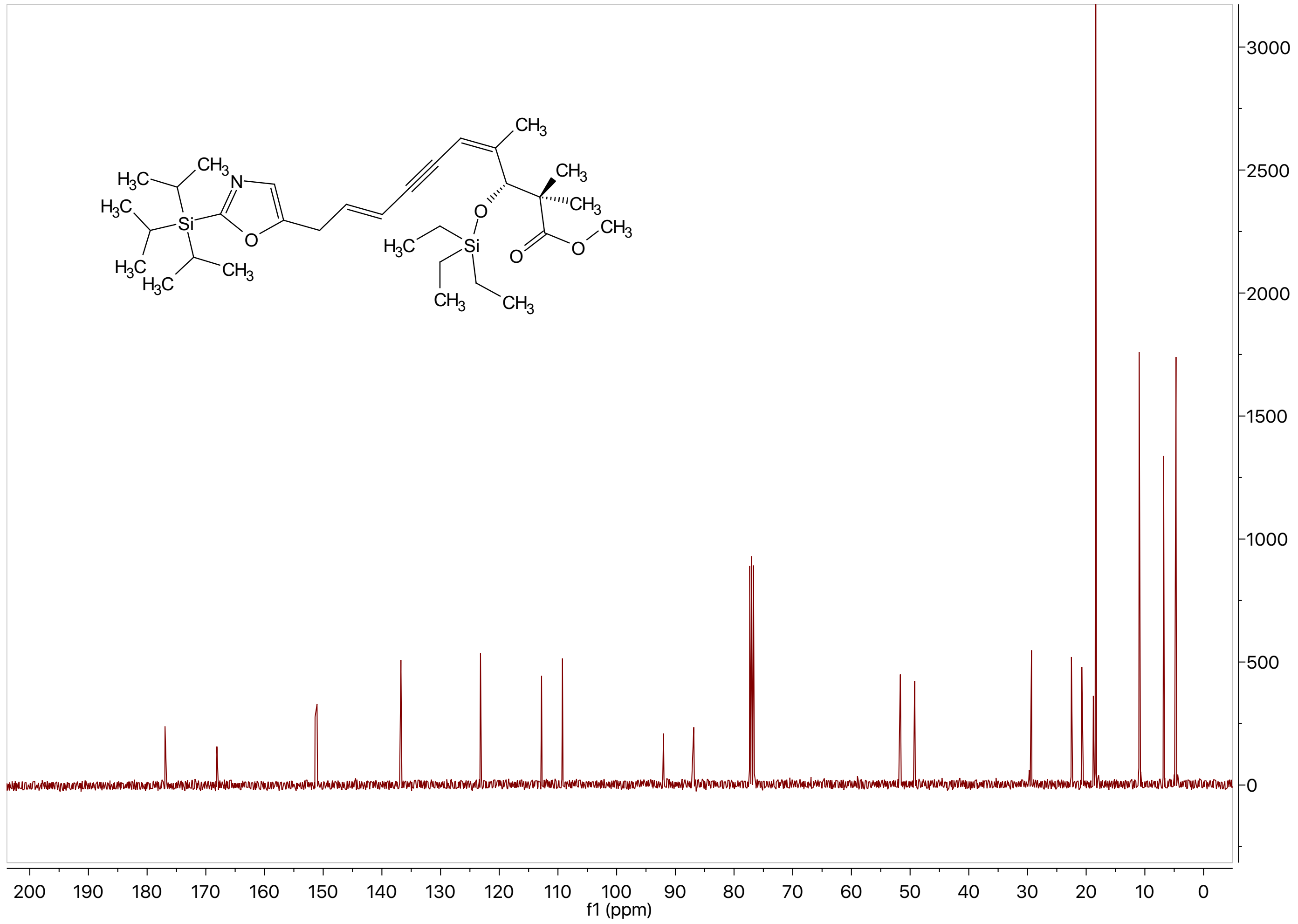

Methyl (R,4Z,8E)-3-hydroxy-2,2,4-trimethyl-10-(oxazol-5-yl)deca-4,8-dien-6-ynoate (25)

^{1}H NMR (500 MHz, CDCl$_3$)

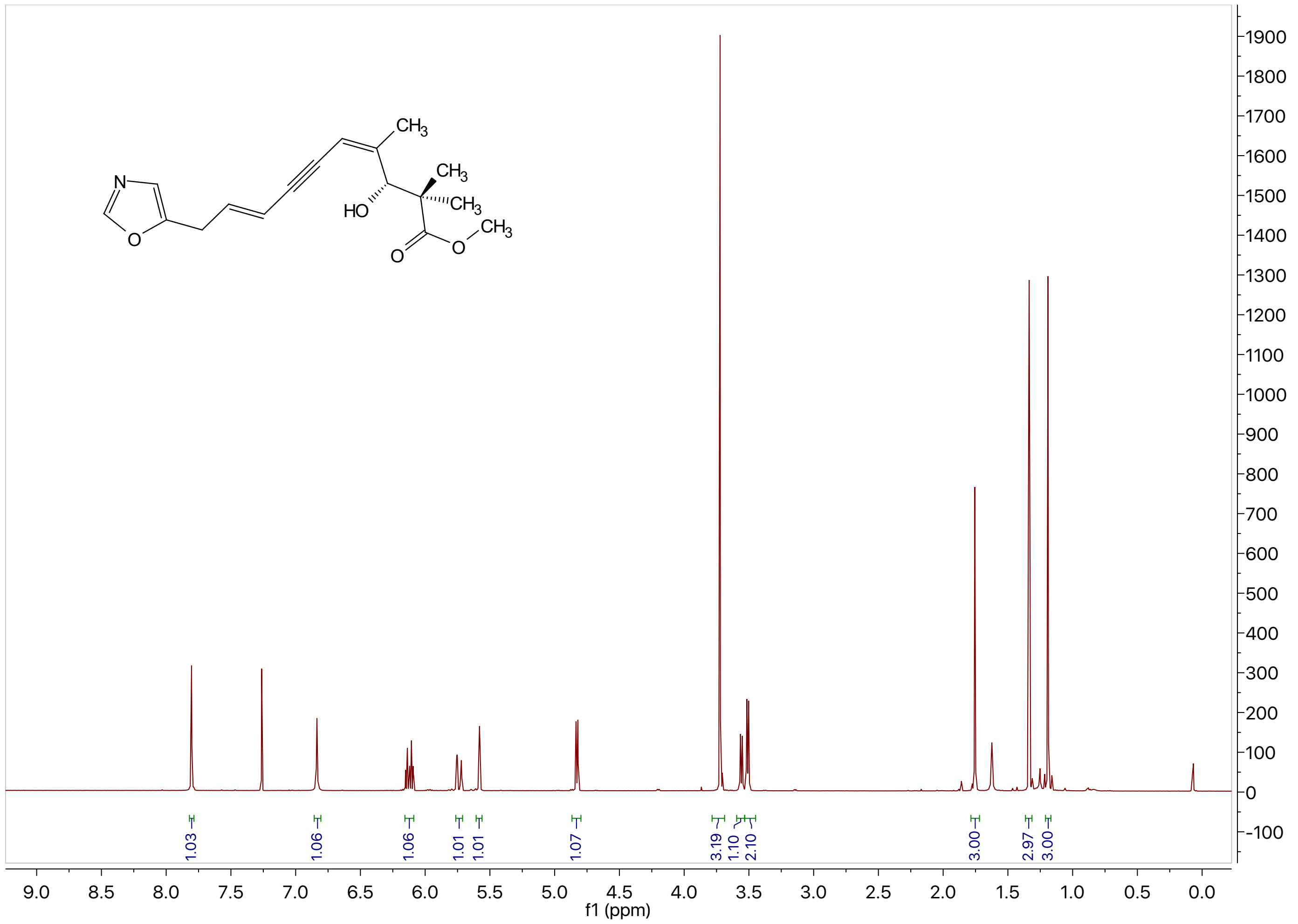

Methyl (R,4Z,8E)-3-hydroxy-2,2,4-trimethyl-10-(oxazol-5-yl)deca-4,8-dien-6-ynoate (25)

^{13}C NMR (125 MHz, $CDCl_3$)

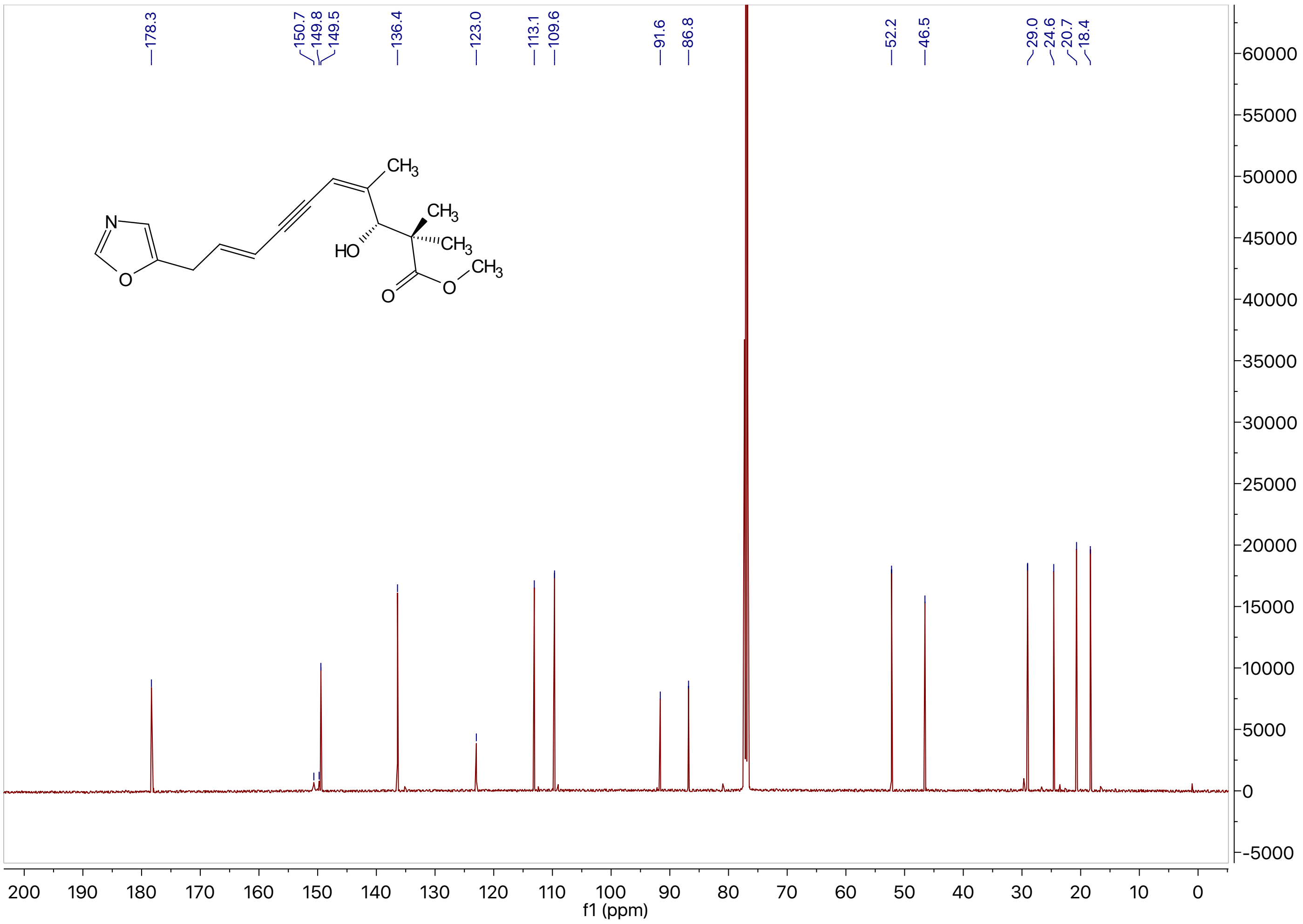

Methyl (R,4Z,6Z,8E)-3-hydroxy-2,2,4-trimethyl-10-(oxazol-5-yl)deca-4,6,8-trienoate (26)

^{1}H NMR (500 MHz, $CDCl_3$)

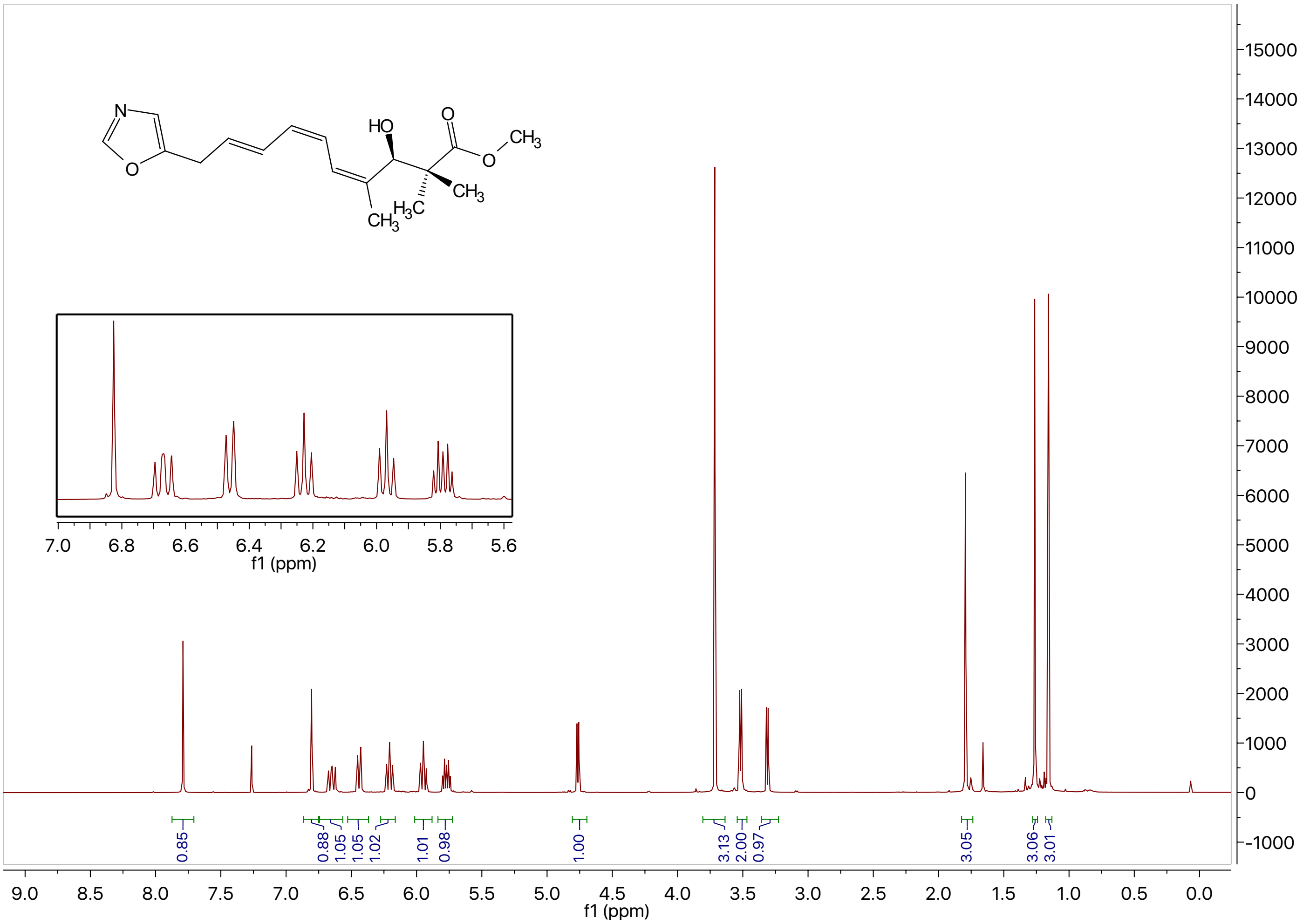

Methyl (R,4Z,6Z,8E)-3-hydroxy-2,2,4-trimethyl-10-(oxazol-5-yl)deca-4,6,8-trienoate (26)

^{13}C NMR (125 MHz, $CDCl_3$)

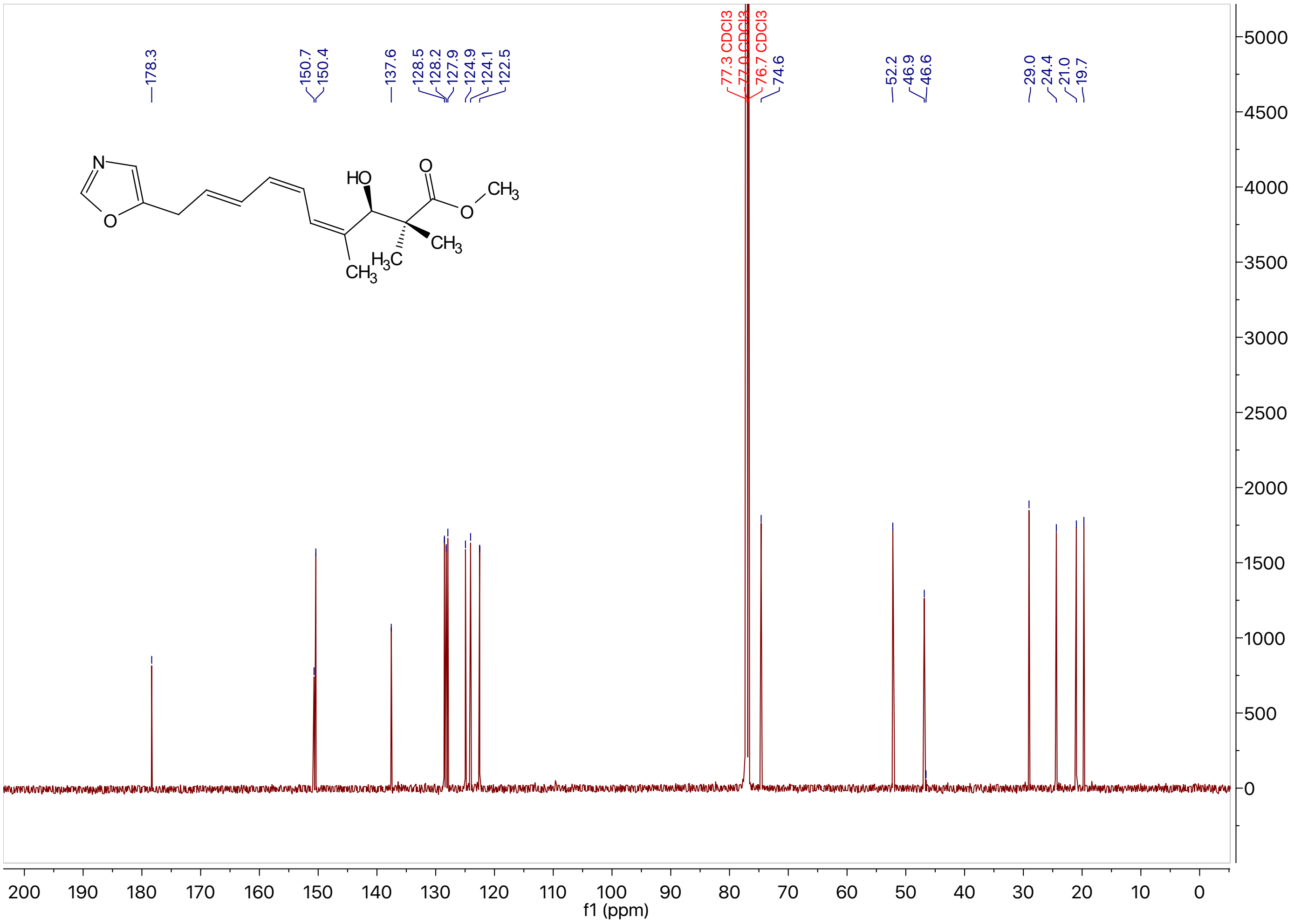

Perfluorophenyl (R,4Z,6Z,8E)-3-hydroxy-2,2,4-trimethyl-10-(oxazol-5-yl)deca-4,6,8-trienoate (27)

^{1}H NMR (500 MHz, $CDCl_3$)

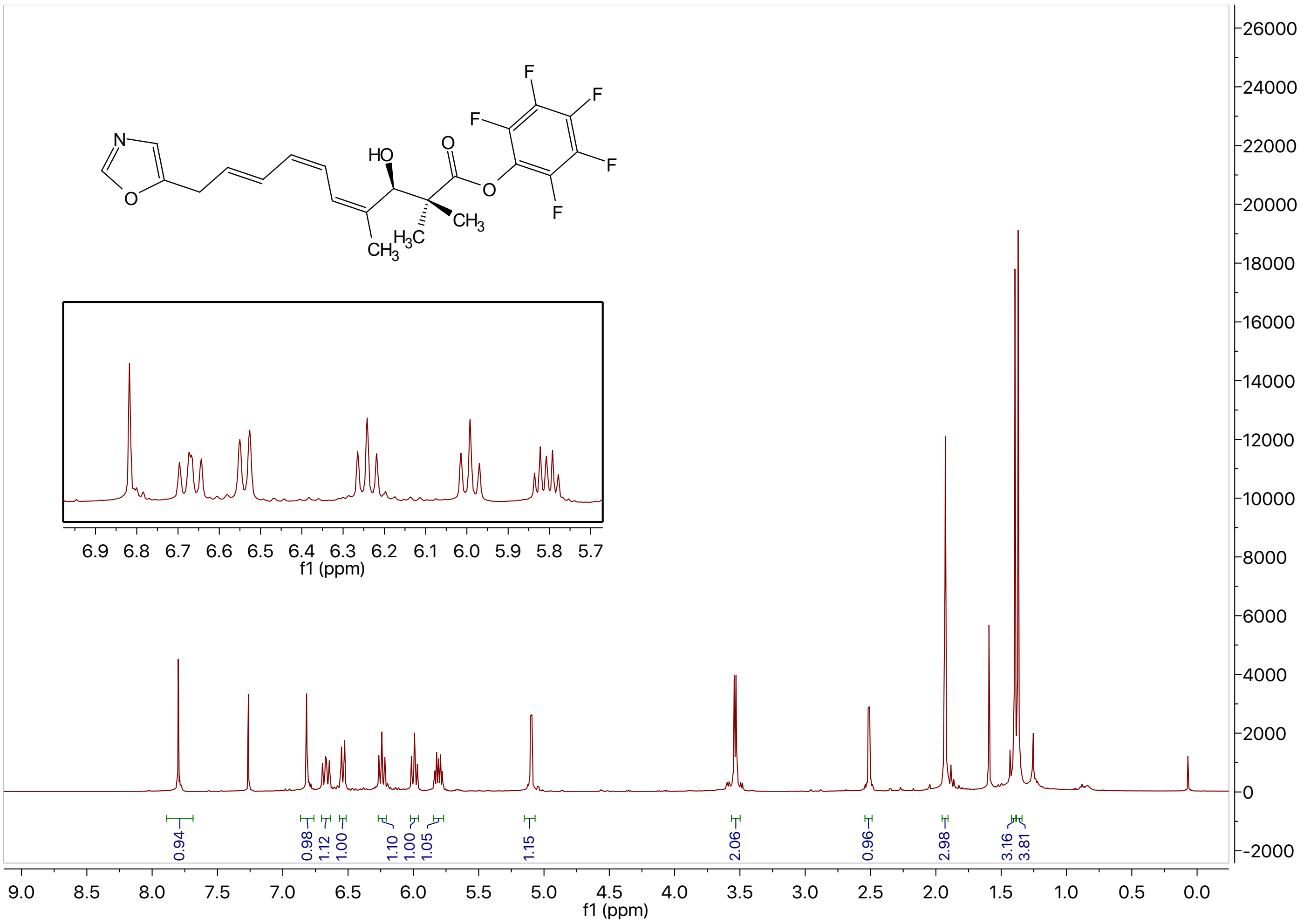

Perfluorophenyl (R,4Z,6Z,8E)-3-hydroxy-2,2,4-trimethyl-10-(oxazol-5-yl)deca-4,6,8-trienoate (27)

^{13}C NMR (125 MHz, $CDCl_3$)

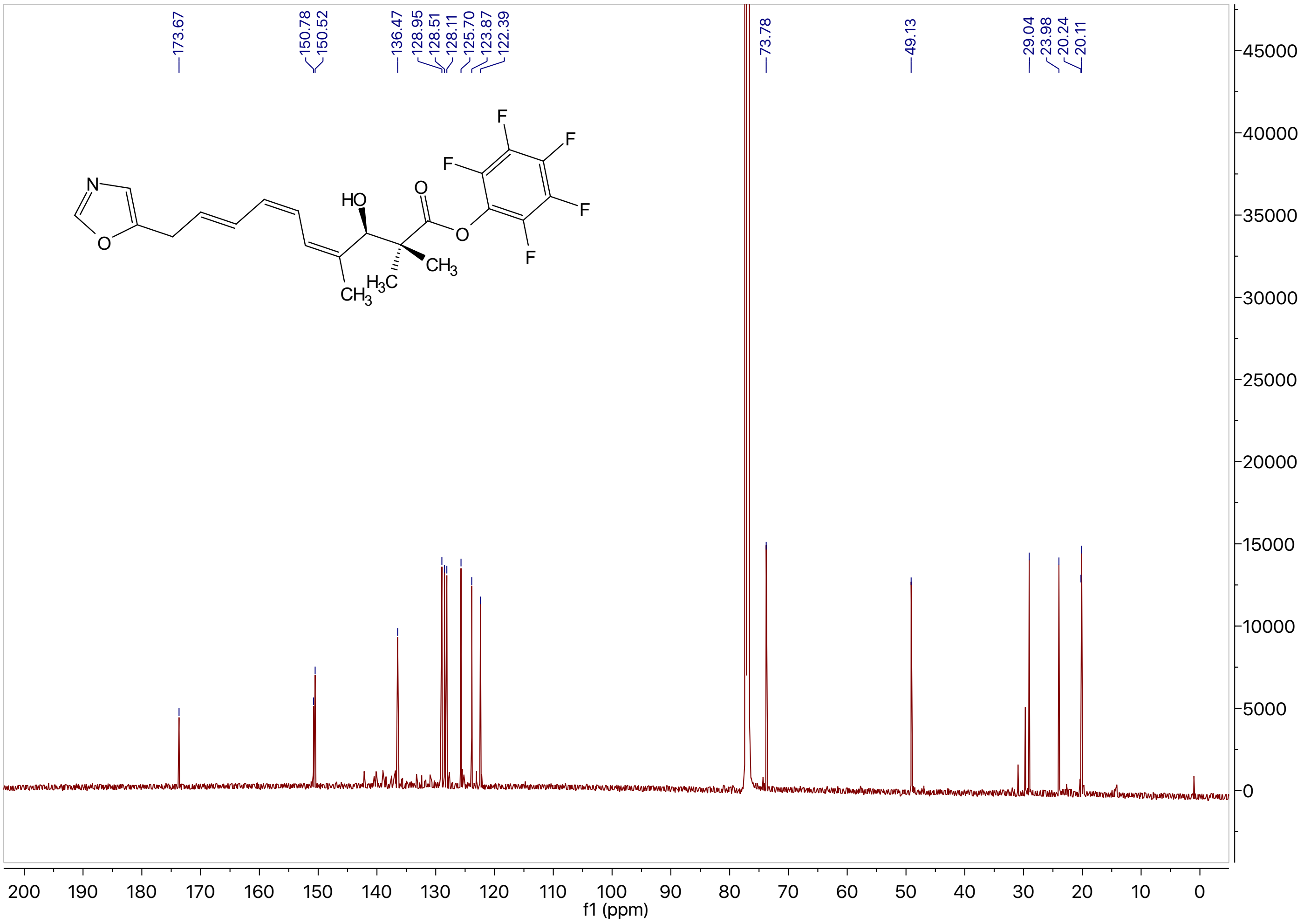

(R,4Z,6Z,8E)-3-Hydroxy-2,2,4-trimethyl-10-(oxazol-5-yl)deca-4,6,8-trienamide (1)

^1H NMR (500 MHz, $CDCl_3$)

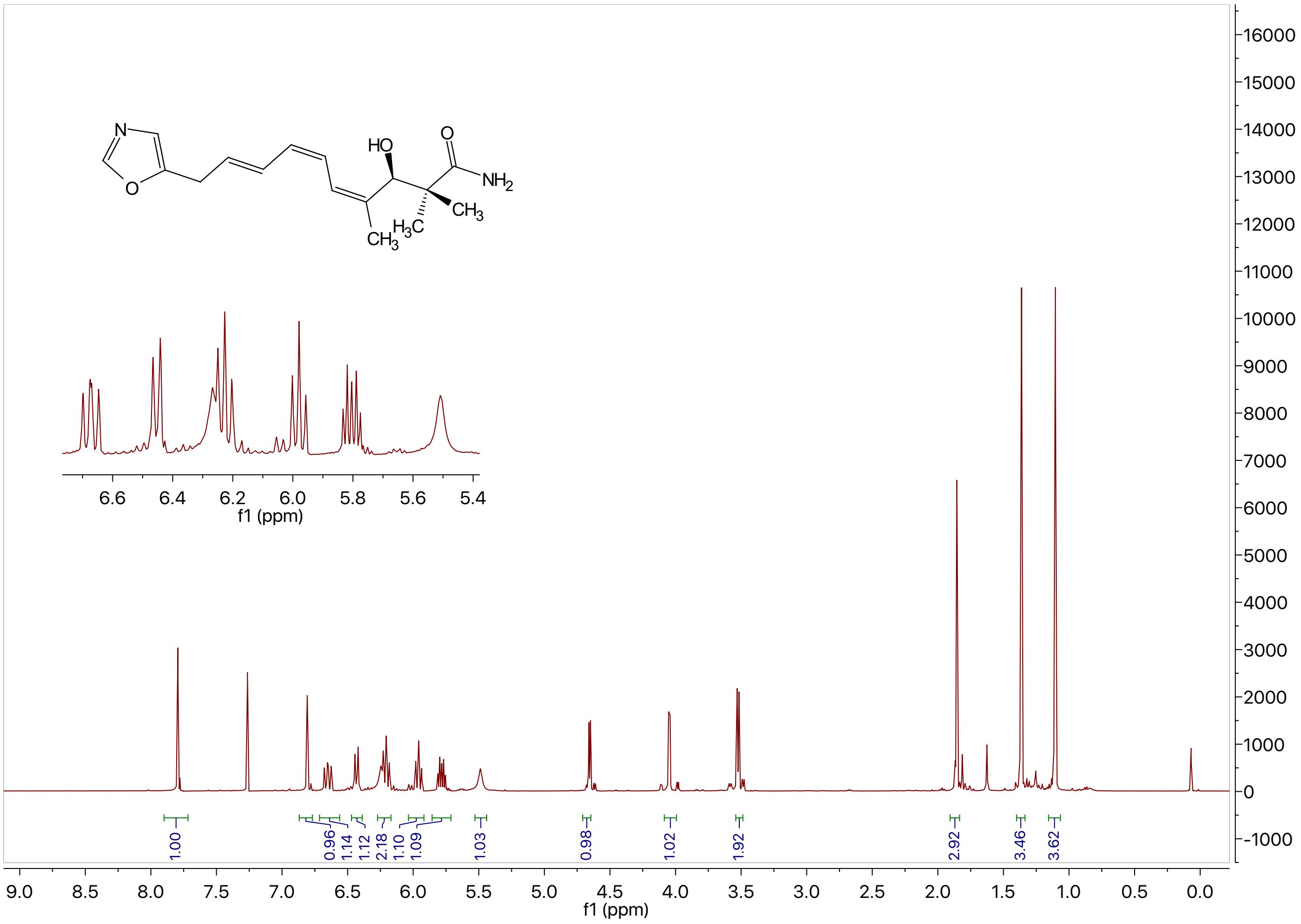

(R,4Z,6Z,8E)-3-Hydroxy-2,2,4-trimethyl-10-(oxazol-5-yl)deca-4,6,8-trienamide (1)

^{13}C NMR (125 MHz, $CDCl_3$)

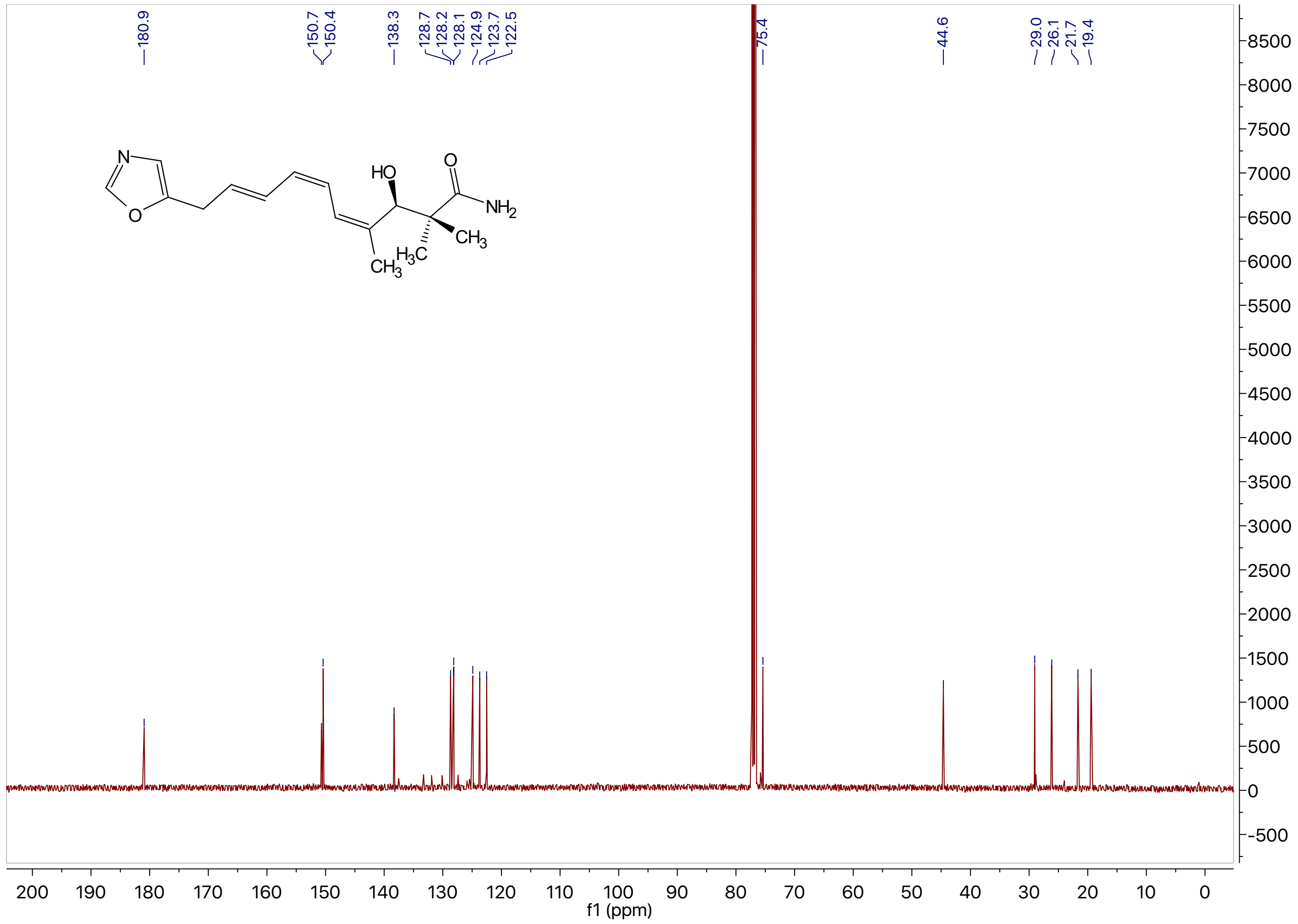

Methyl (S,Z)-5-iodo-2,2,4-trimethyl-3-(((S)-3,3,3-trifluoro-2-methoxy-2-phenylpropan- oyl)oxy) pent-4-enoate (SI-2)

^{1}H NMR (400 MHz, $CDCl_3$)

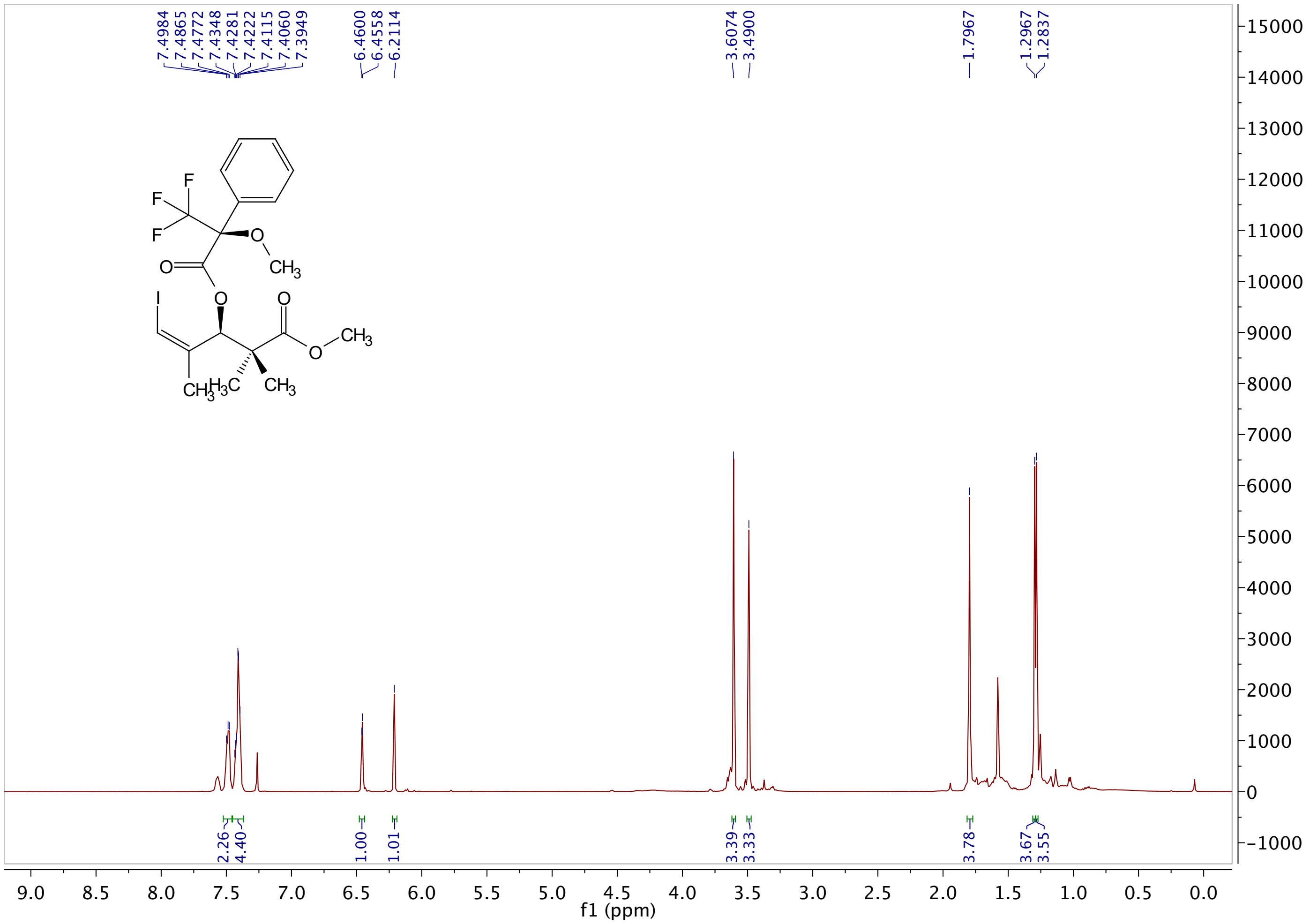

Methyl (S,Z)-5-iodo-2,2,4-trimethyl-3-(((R)-3,3,3-trifluoro-2-methoxy-2-phenylpropanoyl)oxy) pent-4-enoate (SI-3)

^{1}H NMR (400 MHz, $CDCl_3$)

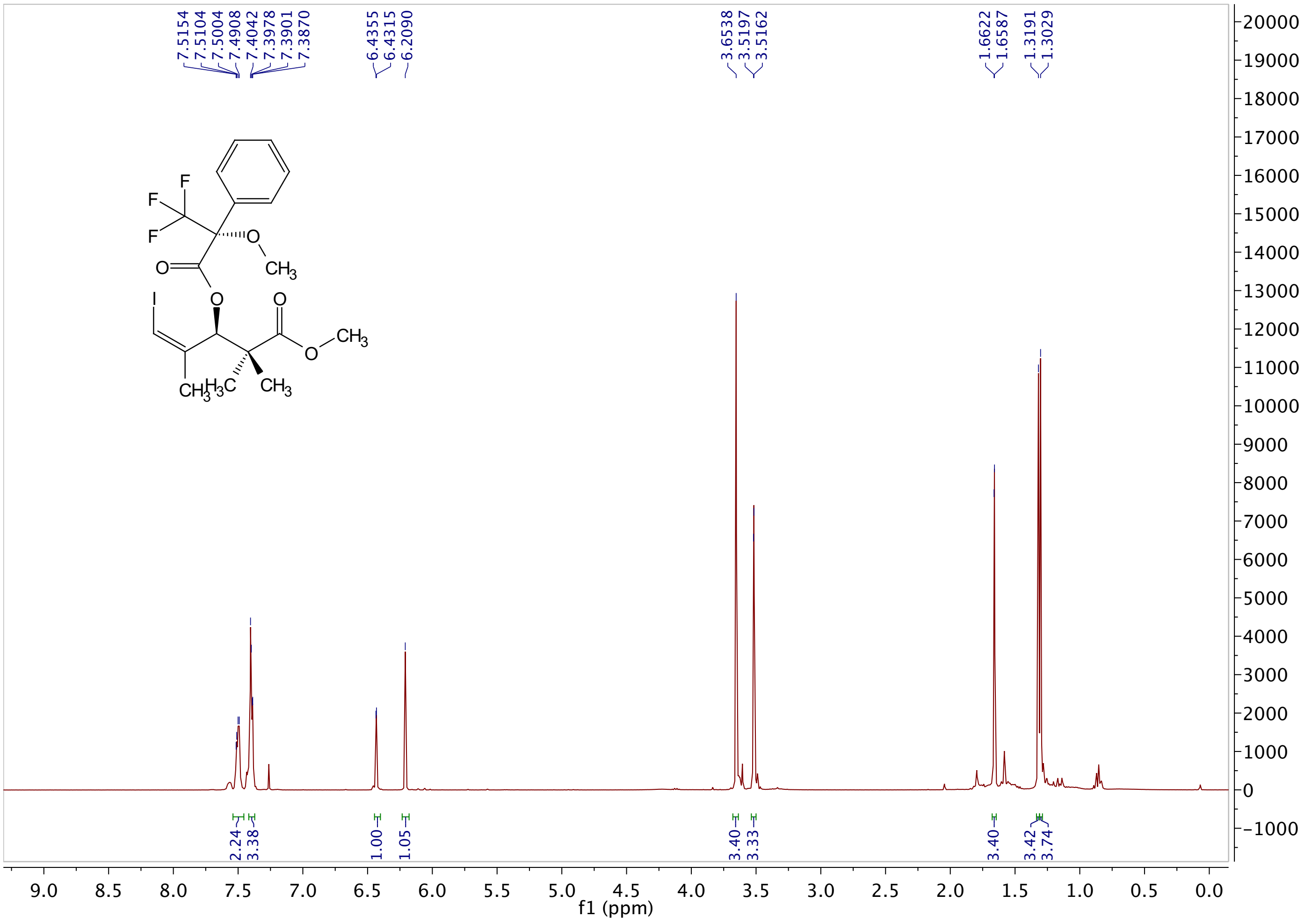

Methyl (S,E)-5-iodo-2,2,4-trimethyl-3-(((S)-3,3,3-trifluoro-2-methoxy-2-phenylpropanoyl)oxy) pent-4-enoate (SI-4)

^{1}H NMR (400 MHz, $CDCl_3$)

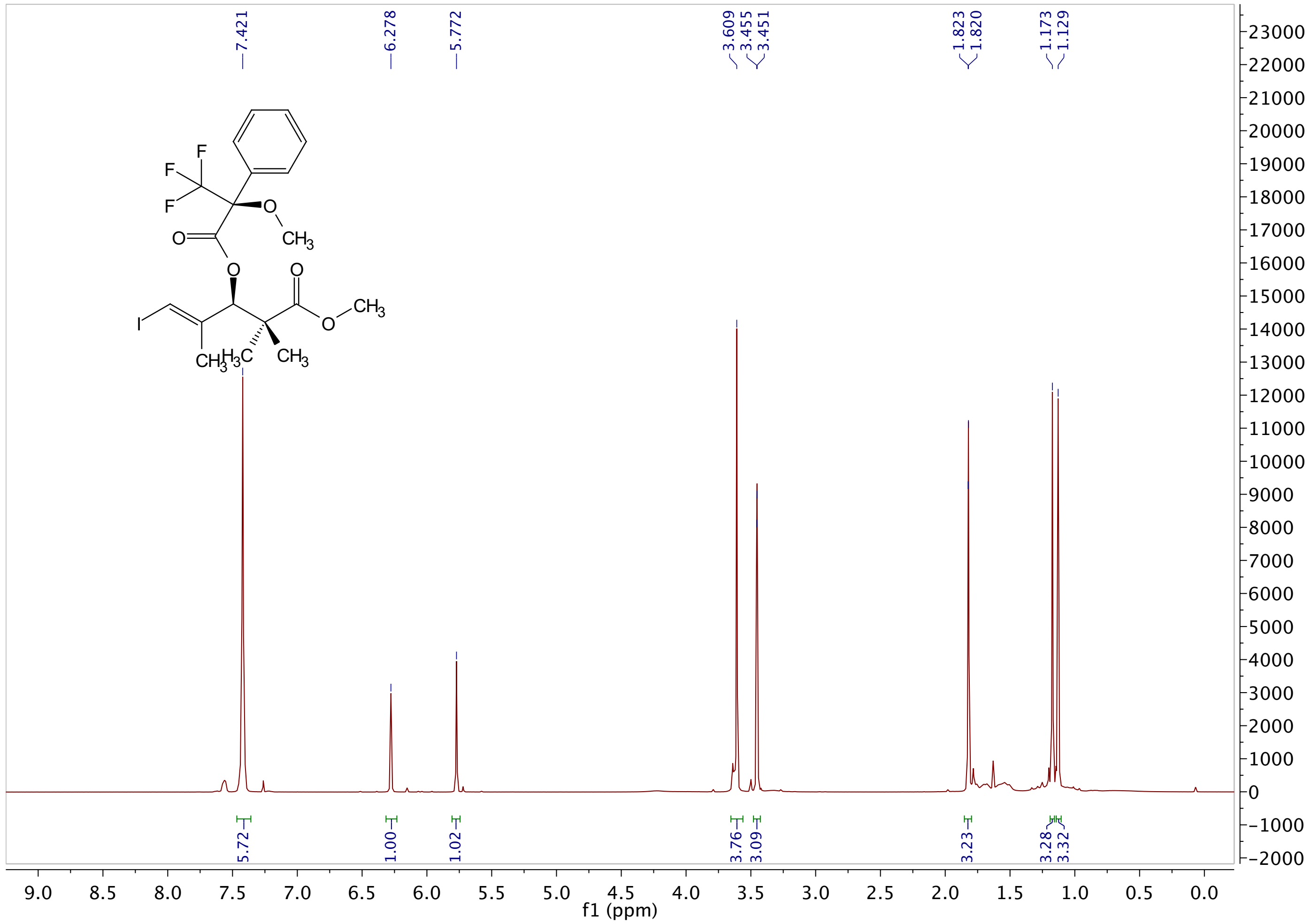

Methyl (S,E)-5-iodo-2,2,4-trimethyl-3-(((R)-3,3,3-trifluoro-2-methoxy-2-phenylpropanoyl) oxy) pent-4-enoate (SI-5)

^1H NMR (400 MHz, $CDCl_3$)

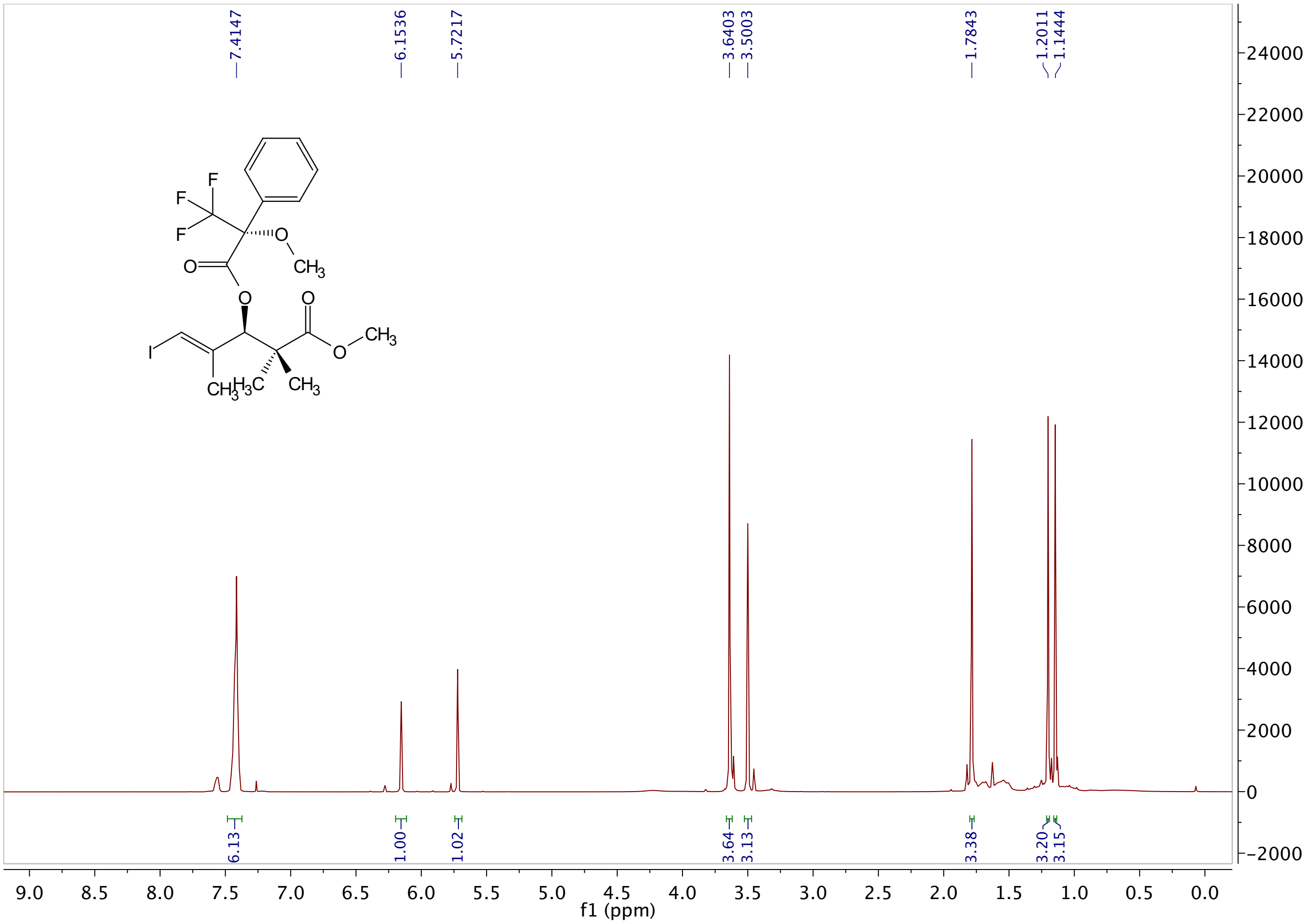

HPLC Graphs

Methyl (Z)-3-hydroxy-5-iodo-2,2,4-trimethylpent-4-enoate (Racemic):

IPA:Hexane (2:98); 0.4 mL/min; OD chiral column

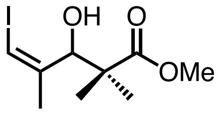

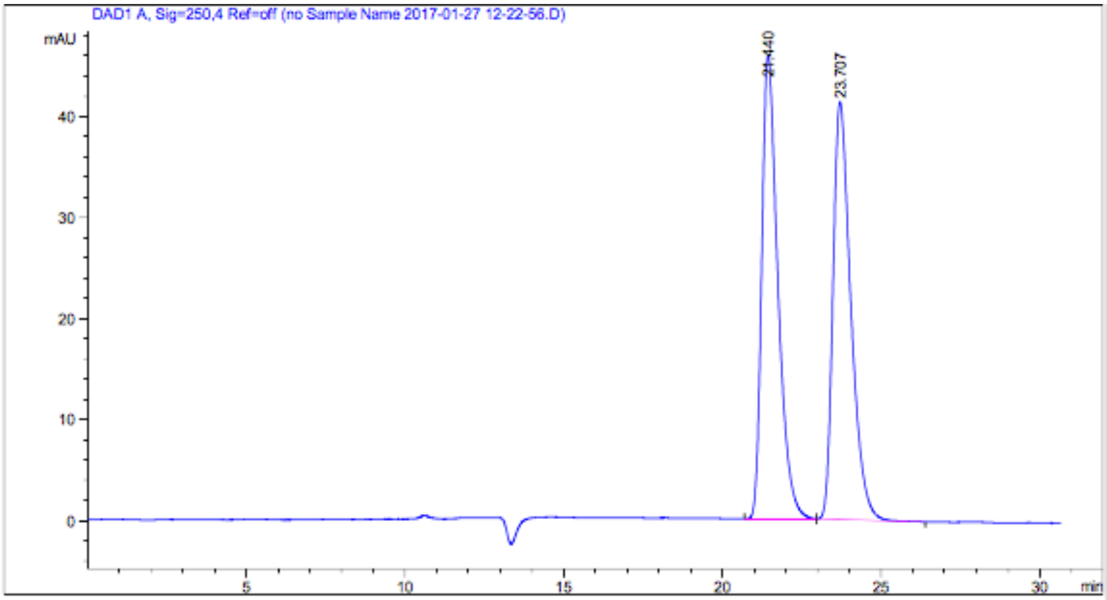


```
================================================================================
                          Fraction Information
================================================================================
Fraction collection off
================================================================================
No Fractions found.
================================================================================
=====================================================================
                        Area Percent Report
=====================================================================

Sorted By             :      Signal
Multiplier            :      1.0000
Dilution              :      1.0000
Use Multiplier & Dilution Factor with ISTDs


Signal 1: DAD1 A, Sig=250,4 Ref=off

Peak RetTime Type  Width     Area      Height     Area
  #   [min]        [min]   [mAU*s]     [mAU]        %
----|-------|----|-------|----------|----------|--------|
   1  21.440 BB    0.5316 1619.07532   45.89038  50.0434
   2  23.707 BB    0.5962 1616.26721   41.33178  49.9566
```

Methyl (S,Z)-3-hydroxy-5-iodo-2,2,4-trimethylpent-4-enoate ((Z)-5):

IPA:Hexane (2:98); 0.4 mL/min; OD chiral column

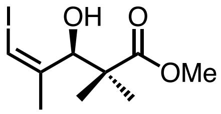

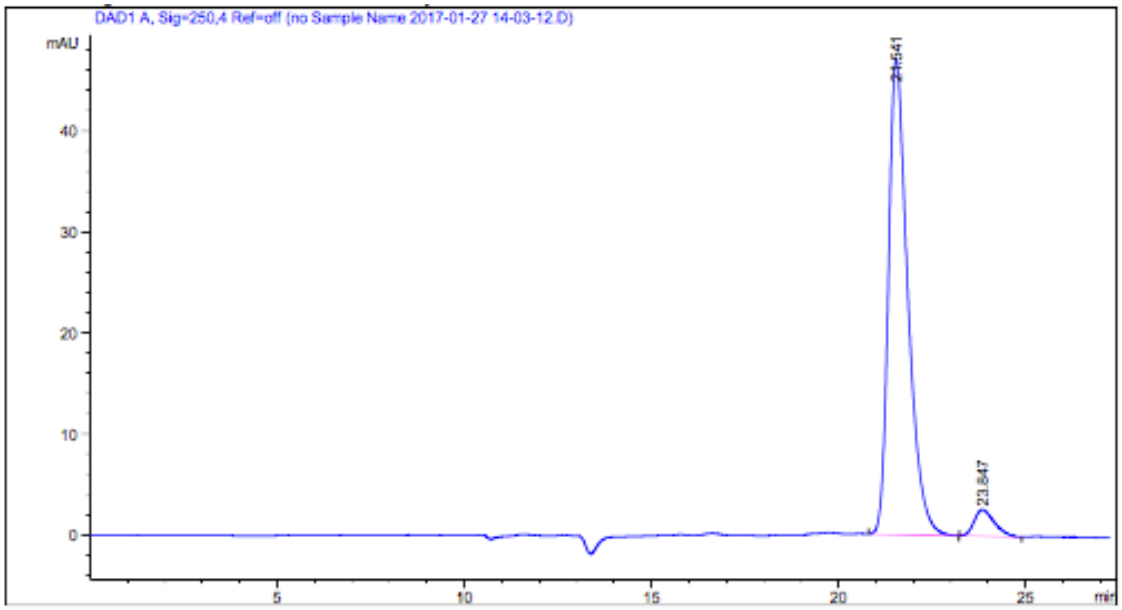

==

Fraction Information

==

Fraction collection off

==

No Fractions found.

==

===

Area Percent Report

===

Sorted By : Signal
Multiplier : 1.0000
Dilution : 1.0000
Use Multiplier & Dilution Factor with ISTDs

Signal 1: DAD1 A, Sig=250,4 Ref=off

Peak #	RetTime [min]	Type	Width [min]	Area [mAU*s]	Height [mAU]	Area %
1	21.541	BB	0.5368	1689.27148	47.05951	94.5003
2	23.847	BB	0.5305	98.31171	2.62737	5.4997

Methyl (S,E)-3-hydroxy-5-iodo-2,2,4-trimethylpent-4-enoate (Racemic):

IPA:Hexane (2:98); 1 mL/min; OD chiral column

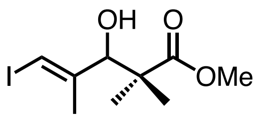

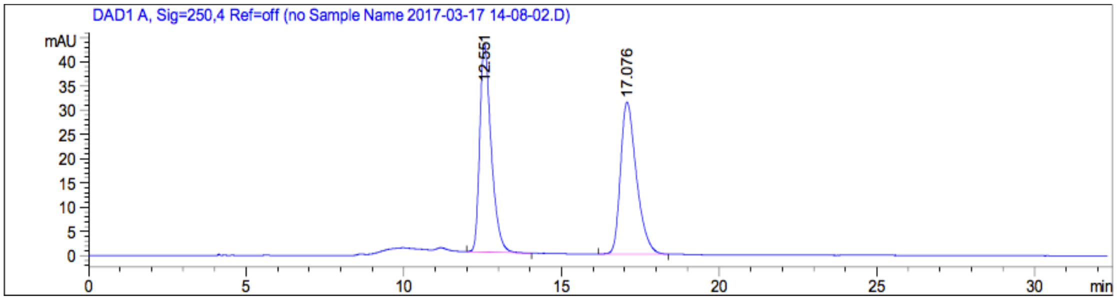

Sorted By : Signal
Multiplier : 1.0000
Dilution : 1.0000
Use Multiplier & Dilution Factor with ISTDs

Signal 1: DAD1 A, Sig=250,4 Ref=off

Peak #	RetTime [min]	Type	Width [min]	Area [mAU*s]	Height [mAU]	Area %
1	12.551	BB	0.3774	1083.54089	43.08933	49.5617
2	17.076	BB	0.5297	1102.70764	31.40315	50.4383

Totals : 2186.24854 74.49248

Methyl (S,E)-3-hydroxy-5-iodo-2,2,4-trimethylpent-4-enoate ((E)-5):

IPA:Hexane (2:98); 1 mL/min; OD chiral column

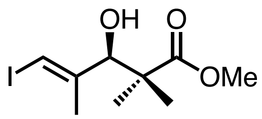

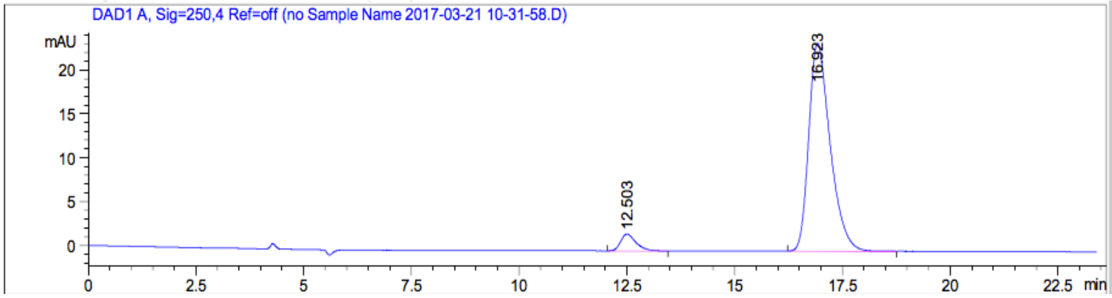


```
Sorted By             :      Signal
Multiplier            :      1.0000
Dilution              :      1.0000
Use Multiplier & Dilution Factor with ISTDs


Signal 1: DAD1 A, Sig=250,4 Ref=off
```

Peak #	RetTime [min]	Type	Width [min]	Area [mAU*s]	Height [mAU]	Area %
1	12.503	BB	0.3654	47.54028	1.92991	5.5064
2	16.923	BB	0.5215	815.83148	23.70828	94.4936

Totals : 863.37177 25.63819

References

[1] K. C. Nicolaou, C. A. Veale, S. E. Webber, H. Katerinopoulos, *J. Am. Chem. Soc.* **1985**, *107*, 7515-7518.

[2] D. Gao, G. A. O'Doherty, *Org. Lett.* **2010**, *12*, 3752-3755.

[3] R. A. Miller, R. M. Smith, S. Karady, R. A. Reamer, *Tetrahedron Lett.* **2002**, *43*, 935-938.

[4] J. G. Duboudin, B. Jousseaume, A. Bonakdar, A. Saux, *J. Organomet. Chem.* **1979**, *168*, 227-232.

[5] E. O. Onyango, J. Tsurumoto, N. Imai, K. Takahashi, J. Ishihara, S. Hatakeyama, *Angew. Chem. Int. Ed.* **2007**, *46*, 6703-6705.

[6] S. Kiyooka, Y. Kaneko, M. Komura, H. Matsuo, M. Nakano, *J. Org. Chem.* **1991**, *56*, 2276-2278.

[7] R. Baker, W. J. Cummings, J. F. Hayes, A. Kumar, *J. Chem. Soc., Chem. Commun.* **1986**, 1237-1239.

[8] B. Vaz, N. Fontan, M. Castineira, R. Alvarez, A. R. de Lera, *Org Biomol Chem* **2015**, *13*, 3024-3031.

[9] M. Yoshino, K. Eto, K. Takahashi, J. Ishihara, S. Hatakeyama, *Org. Biomol. Chem.* **2012**, *10*, 8164-8174.

[10] M. R. Webb, M. S. Addie, C. M. Crawforth, J. W. Dale, X. Franci, M. Pizzonero, C. Donald, R. J. K. Taylor, *Tetrahedron* **2008**, *64*, 4778-4791.

[11] S. Balcells, M. B. Haughey, J. C. L. Walker, L. Josa-Cullere, C. Towers, T. J. Donohoe, *Org. Lett.* **2018**, DOI: 10.1021/acs.orglett.1028b01370.

[12] K. J. Hale, S. Hatakeyama, F. Urabe, J. Ishihara, S. Manaviazar, M. Grabski, M. Maczka, *Org. Lett.* **2014**, *16*, 3536-3539.

[13] K. J. Hale, M. Grabski, S. Manaviazar, M. Maczka, *Org. Lett.* **2014**, *16*, 1164-1167.

[14] B. Wilhelm, S. Nora, S. Christiane, F. Martin, *Helv. Chim. Acta* **1987**, *70*, 1025-1040.

[15] A. S. Kende, K. Kawamura, R. J. DeVita, *J. Am. Chem. Soc.* **1990**, *112*, 4070-4072.

[16] Y. Tanaka, I. Kanaya, K. Shiomi, H. Tanaka, S. Ōmura, *J. Antibiot.* **1993**, *46*, 1214-1218.

[17] a) I. Ohtani, T. Kusumi, Y. Kashman, H. Kakisawa, *J. Am. Chem. Soc.* **1991**, *113*, 4092-4096; b) T. R. Hoye, H. Zhao, *J. Org. Chem.* **2002**, *67*, 4014-4016.
